# Activating dynamic atomic-configuration for single-site electrocatalyst in electrochemical CO_2_ reduction

**DOI:** 10.1038/s41467-023-40970-y

**Published:** 2023-08-28

**Authors:** Chia-Shuo Hsu, Jiali Wang, You-Chiuan Chu, Jui-Hsien Chen, Chia-Ying Chien, Kuo-Hsin Lin, Li Duan Tsai, Hsiao-Chien Chen, Yen-Fa Liao, Nozomu Hiraoka, Yuan-Chung Cheng, Hao Ming Chen

**Affiliations:** 1 Department of Chemistry, National Taiwan University, Taipei, 10617 Taiwan; 2 Material and Chemical Research Laboratories, Industrial Technology Research Institute, Chutung, Hsinchu 31040 Taiwan; 3 Center for Reliability Sciences and Technologies, Chang Gung University, Taoyuan, 333 Taiwan; 4 National Synchrotron Radiation Research Center, Hsinchu, 30076 Taiwan; 5 Japan Synchrotron Radiation Research Institute, 1-1-1 Kouto, Sayo, Hyogo, 689-5198 Japan; 6 Graduate Institute of Nanomedicine and Medical Engineering, College of Biomedical Engineering, Taipei Medical University, Taipei, 11031 Taiwan

**Keywords:** Electrocatalysis, Electrocatalysis

## Abstract

One challenge for realizing high-efficiency electrocatalysts for CO_2_ electroreduction is lacking in comprehensive understanding of potential-driven chemical state and dynamic atomic-configuration evolutions. Herein, by using a complementary combination of in situ/operando methods and employing copper single-atom electrocatalyst as a model system, we provide evidence on how the complex interplay among dynamic atomic-configuration, chemical state change and surface coulombic charging determines the resulting product profiles. We further demonstrate an informative indicator of atomic surface charge (*φ*_e_) for evaluating the CO_2_RR performance, and validate potential-driven dynamic low-coordinated Cu centers for performing significantly high selectivity and activity toward CO product over the well-known four N-coordinated counterparts. It indicates that the structural reconstruction only involved the dynamic breaking of Cu–N bond is partially reversible, whereas Cu–Cu bond formation is clearly irreversible. For all single-atom electrocatalysts (Cu, Fe and Co), the *φ*_e_ value for efficient CO production has been revealed closely correlated with the configuration transformation to generate dynamic low-coordinated configuration. A universal explication can be concluded that the dynamic low-coordinated configuration is the active form to efficiently catalyze CO_2_-to-CO conversion.

## Abstract (web summary)

Comprehensive understanding of Cu state is of high interest for CO_2_ electroconversion. Here, the authors reveal a dynamic low-coordinated Cu center as active site for CO_2_ to CO conversion and propose atomic surface charge as an indicator for evaluating CO_2_ electroreduction performance.

## Introduction

Nowadays, clean energy is crucial for human society to mitigate the damage on the environment, but the intermittent property of such renewable energy restricts a wide application^1^. An electrochemical conversion of carbon dioxide into chemical fuels is a promising approach to store the renewable energy sources^2^. However, a critical challenge toward efficient CO_2_ reduction reaction (CO_2_RR) is attributed to the complexity as a result of diverse reaction pathways. For instance, a two-electron pathway yields CO and HCOOH products, and a twelve-electron one can lead to C2 products, while CH_4_ is produced through an eight-electron route^3^. Therefore, numerous electrocatalysts have been investigated for CO_2_ reduction, and passionate focus has been put on single-atom catalysts owing to their prominent selectivity^4–7^. For instance, atomically dispersed Ni and Fe electrocatalysts are the most efficient catalysts to convert carbon dioxide into carbon monoxide^8,9^, among which Fe single-atom electrocatalyst could achieve a low overpotential of 340 mV at the current density of 94 mA/cm^2^ (see ref. ^8^). Subsequently, exploring the interplay between metal centers and their coordination environments, as demonstrated in the in situ studies of iron single-atom site, has revealed an imperative role of high valence state in selectivity enhancement. Another attractive system—atomically dispersed copper, attainable through an amalgamated Cu–Li method, has been demonstrated to possess unique electrocatalytic activity toward CO_2_RR^10^. However, the exact structure of the copper single-atom sites, their reactivity and, in particular, their dynamic atomic configuration during reaction remain unknown and have been heavily debated^10–14^. Several reactive configurations have been claimed in the literature, including copper coordinated by four nitrogen onto a graphene-like carbon support^11–13^, copper phthalocyanine complexes^15^ and small Cu clusters^10,14^. Note that there is still a disparity in resulting product profiles among similar four N-coordinated sites^11–13,16^. To be specific, single-atom copper encapsulated in N-doped porous carbon has been demonstrated to exhibit a high activity and selectivity toward acetone, which is mainly attributed to the environment of four pyrrolic N atoms that enables a lower energy of CO_2_ activation as well as C–C coupling^16^. In contrast, in some studies, carbon monoxide was revealed to be the only product from CO_2_ reduction through a similar four N-coordinated copper site^11–16^.

To further consider the dominating factors toward diverse products of CO_2_ reduction, it can be expected that production of CH_3_OH and CH_4_ requires an appropriate binding affinity of surface-bonded CO (*CO) to allow further reduction rather than subsequent desorption from the catalyst to generate CO^17–19^. In contrast to C1 product, it can be speculated that the C–C coupling has to occur on the catalytic surface to form the C–C bond^20^. It seems that, however, C–C coupling is unlikely to happen^19^ with the presence of only single-reactive sites in the abovementioned single-atom systems. Despite all these efforts, an in-depth understanding of the interplay between the dynamic configuration and resulting product profiles on single-atom sites is still missing. Furthermore, the selectivity toward various products is strongly correlated to the binding situations of intermediates, which directly relates the assignment of the catalytic activity to specific reactive sites. More importantly, during the potential-driven reaction (i.e., electrochemical CO_2_ reduction), the atomic structure of active sites may considerably change, resulting in unanticipated and adaptive active states. Addressing all these issues requires elaborated and systematic in situ/operando studies^18,21–26^. Herein, we demonstrate a comprehensive investigation of the dynamic restructuring on atomically dispersed Cu sites. By using in situ electrochemical transmission electron microscope (EC-TEM), a first demonstration of imaging dynamic changes of single-atom electrocatalysts during CO_2_ reduction is realized. The complementary application of operando spectroscopies further reveals a potential-driven restructuring on N-coordinated Cu single-atom catalyst (N–Cu SAC), in which the restructuring involves a Cu–N bond-breaking. Such structural reconfiguration can activate the as-prepared N-coordinated Cu SAC to generate an adaptive low-coordinated form that performs significantly high selectivity and activity toward CO product. The atomic surface charge (*φ*_e_) has been demonstrated closely correlated with the configuration transformation into dynamic low-coordinated one (−0.7 V for Cu SAC, −0.3 V for Fe, and −0.4 V for Co SAC), indicating that the atomic surface charge can act as an informative indicator for intrinsic selectivity and activity to the CO_2_RR product profile. A universal explication reveals that the dynamic low-coordinated configuration is the reactive form to catalyze CO_2_ electroreduction toward CO.

## Results

### Structural characterization and in situ EC-TEM analysis

**Fig. 1:**
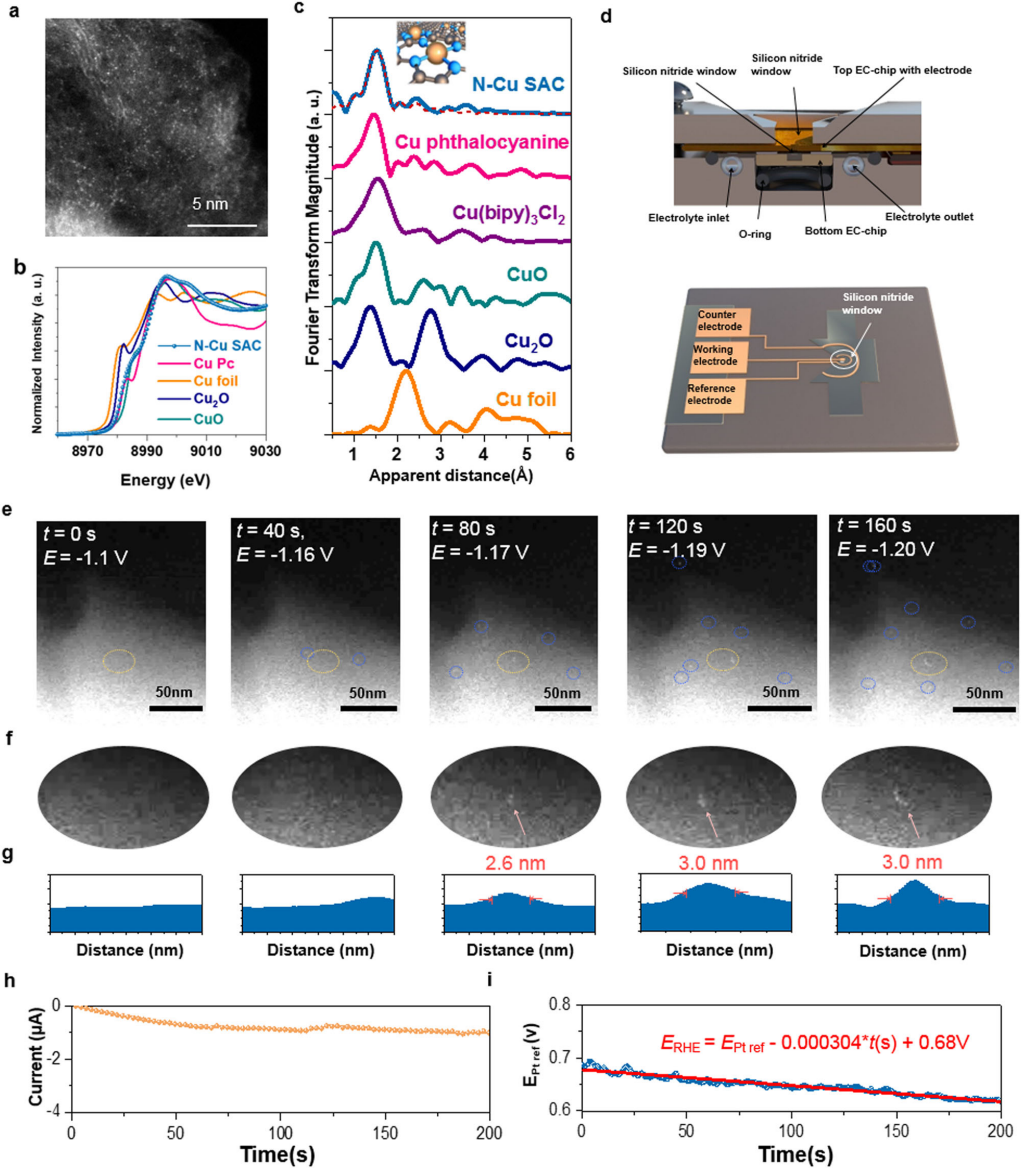
Structural characterization of the as-prepared N–Cu SAC during CO_2_RR. **a** Aberration-corrected HAADF-STEM image of N–Cu SAC. **b** Cu K-edge XANES spectra of N–Cu SAC (blue dotted line) with references of Cu foil (orange), Cu_2_O (blue), CuO (green), and CuPc (pink). **c** R-space EXAFS spectra of Cu K-edge for N–Cu SAC (with fitting result) and references. **d** Schematic of liquid electrochemical TEM and corresponding electrochemical chip. **e** Dark-field STEM images at truly calibrated potentials vs RHE with various duration from 0 to 160 s. **f** Magnified images of selected area in (**e**). **g** Line profiles of selected clusters. **h** Corresponding i–t curve during the TEM characterization. **i** Linear fitting curve for the changes in open-circuit potential vs Pt reference within 200 s as CO bubbling was started.

N–Cu SAC in the form of black powder was prepared by pyrolyzing a mixture of 1-alanine, melamine, and copper acetate monohydrate. Aberration-corrected high-angle annular dark-field scanning transmission electron microscopy (HAADF-STEM) image of N–Cu SAC shows many bright dots with a size of about 1 Å, which represents the existence of single metal atoms on the carbon support (Fig. 1a)^27^. No obvious particles or clusters but only graphene sheets with smooth surface can be observed in low magnified TEM image, HAADF-STEM image, and XRD pattern (Supplementary Figs. S1 and S2). Energy dispersive X-ray spectroscopy (EDX) mapping (Supplementary Fig. S3) manifests a homogeneous distribution of Cu, N and C elements. As for chemical state and local structure, X-ray absorption spectroscopy (XAS) was conducted for the as-prepared N–Cu SAC as well as references of CuPc, Cu foil, Cu_2_O, and CuO (Fig. 1b). The spectroscopic features of as-prepared N–Cu SAC are similar to those of Cu phthalocyanine complexes but significantly different from those of the metal and oxides references, signifying that neither metallic Cu nor metal oxides were formed in N–Cu SAC. In the near-edge region, the intensive feature, which is caused by a dipole transition from 1 *s* to the mixed state of 4 *s* and 4*p*, is indicative of the Cu(II) oxidation state in the as-prepared N–Cu SAC. Fourier-transformed k^3^-weighted extended X-ray absorption fine structure (EXAFS) spectra of the as-prepared N–Cu SAC and N-coordinated references of Cu(bipy)_3_Cl_2_ and Cu phthalocyanine complexes exhibit a main peak located at approximately 1.5 Å, which results from a single scattering by the nearest neighboring atom of N (Fig. 1c). Notably because no distinct peak is related to Cu–Cu scattering path, one can confirm that Cu(II)–N species are dispersed as a single-atom form in the as-prepared Cu SAC sample, which coincides well with the findings above. The fitted EXAFS data reveals that nitrogen coordinates to Cu with a coordination number of 3.8 ± 0.4 (Supplementary Table S1). Accordingly, we conclude that the as-synthesized N–Cu SAC is atomically dispersed with a fourfold N-coordinated configuration (4N–Cu SAC).

Although various operando techniques have been applied to elucidate the mechanistic understandings of electrocatalysts during CO_2_ reduction^9,28–31^, to date, direct visualization of the catalyst behaviors under a potential-driven condition has not been achieved. Liquid electrochemical TEM (EC-TEM)^32,33^ was realized to track the dynamic structural changes of the Cu SAC electrocatalyst during CO_2_ reduction (Fig. 1d–g and Supplementary Movies S1–6). The Cu SAC was drop-casted on the working electrode as shown in Fig. 1d, in which Pt electrodes were acting as working, counter and reference electrodes (Protochips). The applied potential was calibrated to reversible hydrogen electrode (RHE). Given that the primary product of CO_2_RR on SAC is CO, the CO poisoning effect on the open-circuit potential drift during reactions must be taken into account since CO is a strong poison for Pt surfaces (more details in Supplementary Materials). We applied the constant potential of −1.83 V vs Pt reference for realizing in situ TEM measurement under −1.1 V vs RHE. Based on the linear fitting for the changes in open-circuit potential (vs Pt reference) within 200 s (Fig. 1h, i), the correctly calibrated potentials vs an actual RHE with various durations were obtained (−1.1 V at 0 s, −1.16 V at 40 s, −1.17 V at 80 s, −1.19 V at 120 s and −1.20 V at 160 s). At beginning (−1.1 V vs RHE), only a smooth surface with the graphene-like feature is observable (Supplementary Movie S1). It has to be noticed that the single-atom feature is unlikely to be detected under such liquid condition owing to the poor mass-scattering contrast in the liquid electrolyte (Fig. 1e). Apparently, two spots (encircled in orange) with a diameter of around 2.6 nm (as depicted in line profiles of Fig. 1g) appear after 80 s of electrolysis, implying the occurrence of restructuring and the formation of clusters. With increasing duration (up to 120 s), more bright spots are revealed, and the line profile denotes a slight growth of size to reach about 3.0 nm. After electrolyzing for longer duration (Supplementary Figs. S4 and S5 and Supplementary Movie S2), in situ formed clusters are evidently discernable with almost unchanged diameter. Observed clusters which were caused by Pt electrode oxidation can be excluded because of absence of Pt on the electrode (Supplementary Fig. S6), which indicates that bright spots arise from the formation of Cu clusters rather than Pt re-deposition^34–36^. By contrast, when performing TEM measurement at small cathodic potential of −0.6 V vs RHE (Supplementary Movie S3) or without applying cathodic potentials (Supplementary Movie S4), one could not detect any bright spots on the surface throughout the process. This phenomenon evidently reveals a fact that a specific amount of cathodic potential is essential for driving such structural reconstruction. On the other hand, in the absence of liquid electrolyte, no bright spots on the surface are present (Supplementary Movie S5), further indicating that the formation of Cu clusters originates from the potential-driven restructuring rather than the electron damage. Notably, as indicated by the blue circles in Fig. 1e and Supplementary Fig. S5, these clusters seem to be able to migrate freely on the carbon support. Meanwhile, the current rapidly increases and reaches steady at around 1.0 μA in about 2 min (Fig. 1h), further suggesting that the cluster formation leads to an increase in the corresponding current. It is worthwhile to mention that even though a few studies have reported the formation of small clusters^10,14^, this is the first imaging evidence of such small clusters’ formation process during CO_2_RR, which also verifies a finding that small clusters can freely migrate on the graphene-like support because of both C–N and Cu–N bond-breaking (vide infra).

### In situ X-ray absorption/emission spectroscopic analyses on N-coordinated Cu SAC

**Fig. 2:**
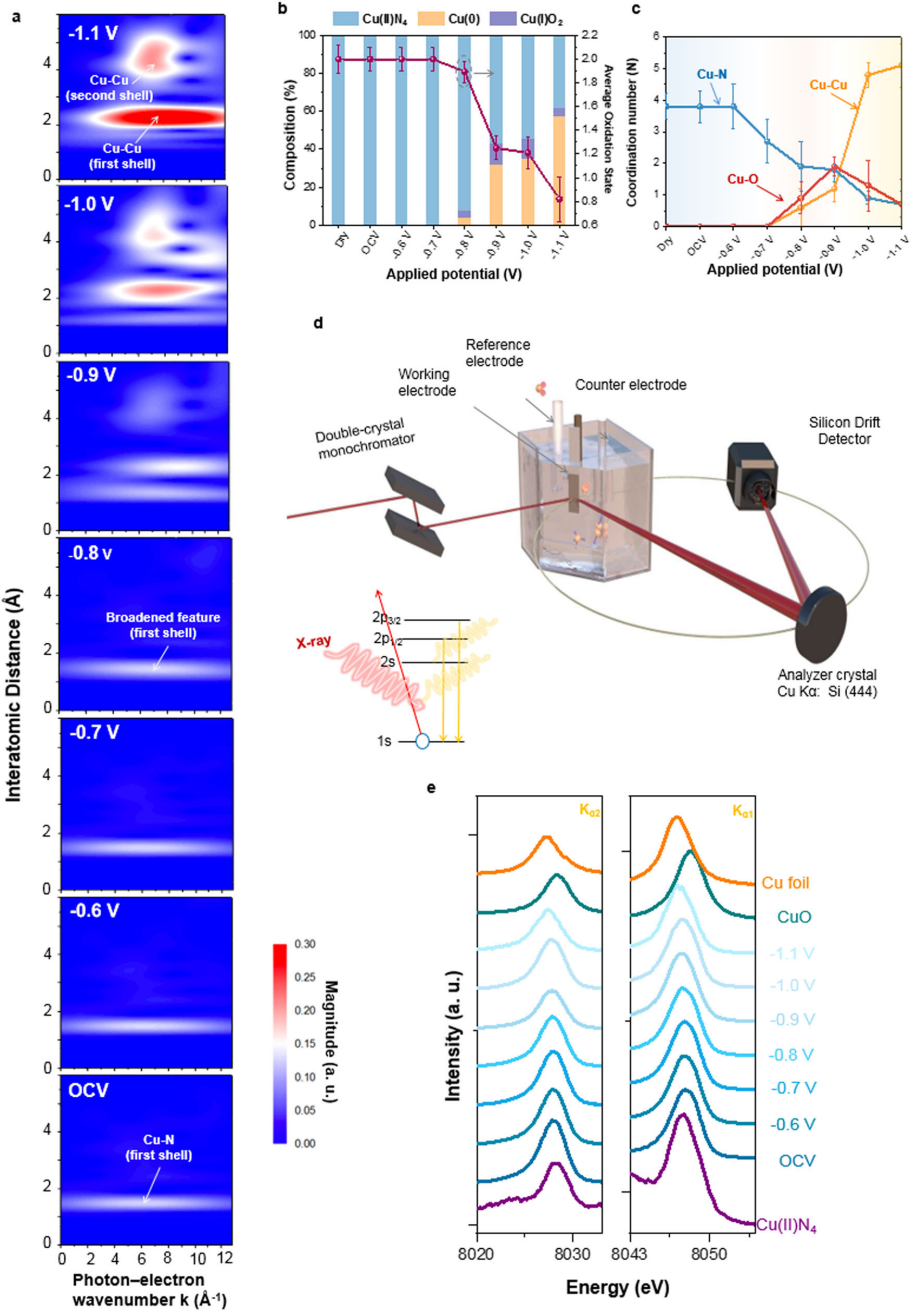
In situ X-ray spectroscopy analysis of N–Cu SAC during CO_2_RR. **a** Wavelet-transform diagram of in situ Cu K-edge EXAFS spectra of N–Cu SAC at various applied potentials in CO_2_-saturated 0.1 M KHCO_3_ solution during CO_2_ reduction. **b** Extracted oxidation state from in situ Cu K-edge XANES spectra of N–Cu SAC through linear combination of Cu foil, Cu_2_O, and Cu(bipy)_4_Cl as spectroscopic references in CO_2_-saturated 0.1 M KHCO_3_ solution. **c** Quantitative analysis of coordination environment extracted from EXAFS fitting at various potentials in CO_2_-saturated 0.1 M KHCO_3_ solution. Error bars represent the standard deviation of three independent measurements. **d** Schematic representation of in situ X-ray emission spectroscopy (XES) apparatus applied to a liquid electrochemical cell and energy diagram for the detection of Kα_1_ and Kα_2_ emission in transition-metal ion. **e** In situ XES spectra of Kα_1_ and Kα_2_ emission for N–Cu SAC and references collected at various potentials during CO_2_ reduction in CO_2_-saturated 0.1 M KHCO_3_ solution. All measurements were performed in 0.1 M KHCO_3_ by a typical three-electrode setup using glassy carbon as the working electrode, Hg/HgCl_2_ electrode and platinum plate acted as reference and counter electrodes, respectively. The applied potential was calibrated to a reversible hydrogen electrode (RHE).

Since no information regarding the chemical state of forming clusters can be revealed by operando EC-TEM, in situ XAS was performed to unveil the chemical state as well as local structures (Fig. 2a and Supplementary Figs. S7 and S8). From wavelet-transformed (WT) EXAFS spectra, one can see a single strong WT signal focusing at the interatomic distance of 1.6 Å, implying that Cu ions in the as-prepared N–Cu SAC are mainly coordinated with light atoms (i.e., N atoms) and feature with single-atom nature (Fig. 2a). Once the applied cathodic potential reached −0.8 V vs RHE, it was observed that the WT signal at 1.6 Å was broadened, indicating that the coordination environment around Cu sites started to change at this potential, which can be expected to originate from an interference caused by another scattering path. Upon −0.9 V, another intensive signal focusing at 2.3 Å and higher *k* values on 8.8 Å^−1^ appeared and can be attributed to the Cu–Cu scattering path, which corroborated the characteristic peaks at ~2.3 Å in the Fourier-transformed EXAFS spectra (Supplementary Fig. S8), suggesting the formation of Cu clusters. When the applied potential decreased to −1.0 and −1.1 V vs RHE, the remarkably intense peaks situated at 4–5 Å were present due to a second-shell and/or multiple scattering in Cu clusters, which resulted from successive aggregation of Cu atoms on the carbon support. Such formed Cu clusters can be further validated by in situ XRD measurement, in which diffraction peaks at 43° and 50°, respectively, assigned to Cu(111) and Cu(200) were clearly observed (Supplementary Fig. S9).

As for the chemical state of Cu sites, in situ Cu K-edge XANES spectra can provide useful features to reveal the dynamic changes in Cu species as a function of applied potentials (Supplementary Fig. S7). Specifically. once the applied potential decreased to −0.8 V vs RHE, a feature peak at ~8982 eV that corresponds to the CuO_x_ species remarkably enhanced, suggesting that Cu–O bond may generate at this potential. As the potential further decreased (e.g., −1.1 V vs RHE), the N–Cu SAC showed a similar edge feature to that of Cu foil at ~8980 eV, supporting the views that partial Cu single atoms aggregated into clusters with gradually formed metallic Cu species. To further clarify the generation of Cu–O moiety, as revealed by the advent of characteristic bands based on in situ Raman (Raman bands at ~525 cm^−1^ and ~630 cm^−1^) and surface-enhanced infrared absorption spectroscopy (SEIRAS) (absorption bands at ~1130 cm^−1^) (Supplementary Figs. S10 and S11), one may suggest the possible formation of Cu–O species on N–Cu SAC during CO_2_RR. It is postulated that the identified Cu–O species might potentially arise from surface-adsorbed species formed as a result of the complex interplay between the catalytic surface and the electrolyte. However, it is important to recognize that the observed spectroscopic features may not directly correspond to the reactive intermediate species involved in the CO_2_ reduction reaction. This limitation is attributed to the inherent temporal resolution constraints of Raman spectroscopy and SEIRAS. In order to overcome this limitation, a simultaneous consideration of Cu–N, Cu–O, and Cu–Cu contributions is implemented for quantitative analyses of in situ Cu K-edge XANES spectra, achieved through accurate linear combination (Fig. 2b and Supplementary Fig. S12). At the beginning stage, the composition of N–Cu SAC was made of almost ~100% Cu(II)N_4_, resulting in an average oxidation state of +2.0. No significant deviation from the original state was noticed until the applied potential reached −0.8 V vs RHE, in which Cu(0) and Cu(I)_2_O appeared and oxidation state started to decline. Remarkably, at −0.9 V vs RHE, ~43% of the composition evolved into metallic Cu(0) and Cu(I)_2_O with the oxidation state declining to +1.3. At the final stage of −1.1 V vs RHE, the resulting sample was characteristic of about 57% Cu(0), 5% Cu(I)_2_O, and 38% Cu(II)N_4_ with the oxidation state of 0.8. Notably, in spite of the high cathodic potential applied, a portion of Cu(I) still existed. This phenomenon is consistent with the results in several in situ studies regarding Cu chemical state, which have declared the presence of Cu(I) state under high cathodic potentials originated from the interplay with electrolyte^28,29,37–39^. By contrast, the linear combination analysis on Cu L-edge spectra obtained under an ultra-high vacuum environment showed that, after 1 h of CO_2_RR at −1.1 V vs RHE, the composition of the catalyst was composed of 11% Cu(I)_2_O, 14% Cu(II)N_4_ and 75% Cu(II)O with +1.9 oxidation state (Supplementary Fig. S13). The relatively higher oxidation state of Cu sites observed by L-edge XAS spectra can be ascribed to the appearance of rapidly oxidized species upon releasing the applied potentials. One should note that the linear combination analysis strongly relies on the reasonable identification of existing components in samples, and thus the pre-determination of dynamic Cu species on N–Cu SAC during CO_2_RR is a prerequisite for an accurate quantitative analysis. To reveal actually existing species/structures under working conditions, more cutting-edge characterizations are encouraged to provide comprehensive information, especially those energy- and time-resolved methods. Furthermore, although there may be slight variations in the results due to the selection of different reference samples for the linear combination analysis, this method still offers valuable insights into the dynamic transformations of catalyst species during reactions. It serves as evidence for identifying the evolving chemical states of electrocatalysts in a dynamic manner.

A quantitative EXAFS analysis with regard to the coordination environment was further performed (Supplementary Figs. S14 and 15, see details in Supplementary Materials). Note that, the integrated XANES, Raman and SEIRAS results discussed above indicated the possible existence of surface-adsorbed Cu–O species during the CO_2_RR in addition to Cu–N bond, thus the multipath fitting for the first shell with both Cu–N and Cu–O scattering paths taken into account may be essential. As compared with those of single-path fitting, the multipath fitting results matched well with experimental data (Supplementary Fig. S16). The fitting results were summarized in Fig. 2c and Supplementary Table S2, upon −0.7 V vs RHE was applied, the coordination number (CN) of Cu–N declined to 2.7, which suggested the formation of a low N-coordinated Cu single-atom site due to the Cu–N bond weakening/breaking during CO_2_RR. Significantly, such low N-coordinated configuration can be clarified to be strongly correlated with the selective CO pathway (vide infra). The analysis further demonstrated that no surface-adsorbed Cu–O species were observed in EXAFS spectra at −0.7 V vs RHE, which can be attributed to an unstable Cu–O configuration resulting from the subtle adsorption of oxygen-related groups from the electrolyte at the initial stage of CO_2_RR. As the cathodic potential was increased (<−0.8 V vs RHE), the coordination number of Cu–N showed a continuous decrease, while the coordination number of the Cu–O path increased and reached a maximum value of 1.9 at −0.9 V vs RHE, eventually dropping to 0.7 at −1.1 V vs RHE. Simultaneously, the coordination number of Cu–Cu significantly increased with increasing potentials, reaching 5.1 ± 0.7 at −1.1 V vs RHE. Consequently, the in situ XAS analyses suggest that the formation of the surface-adsorbed Cu–O moiety is initially favored by the strong adsorption of oxygen-related groups from the electrolyte once the atomic Cu sites detach from the initial N-coordinated environment. Subsequently, these Cu–O species undergo further aggregation into clusters before being reduced to metallic Cu(0), resulting in a substantial presence of the Cu(I) state.

Furthermore, an in situ X-ray emission spectroscopy (XES) of Cu Kα line was conducted to qualitatively unveil the dynamic variations during the cluster formation (Fig. 2d, e)^40–43^. Kα_1_ emission was taken as a typical indicator, prior to −0.8 V versus RHE, the main peak of N–Cu SAC at ~8047.6 eV, corresponding to that of Cu(II)N_4_ reference, can be ascribed to the Cu–N coordination. Intriguingly, with increasing applied potentials (<−0.8 V vs RHE), one can observe a considerable gradual-shifting of Kα_1_ emission peak towards lower energies (~8047.1 eV) where the Kα_1_ emission peak of Cu foil was located at, suggesting the dynamic restructuring to metallic Cu clusters on the N–Cu SAC during CO_2_RR. Based on all results discussed above, it can be inferred that, at the early stage, the potential-driven Cu sites are capped and stabilized by surface hydroxyl group prior to forming the metallic Cu clusters, which might be the major reason for causing the aforementioned structural transformation of forming metal oxide^44,45^.

### Intermediates-catalyst interaction revealed by in situ high-energy resolution fluorescence detected XAS (HERFD-XAS)

**Fig. 3:**
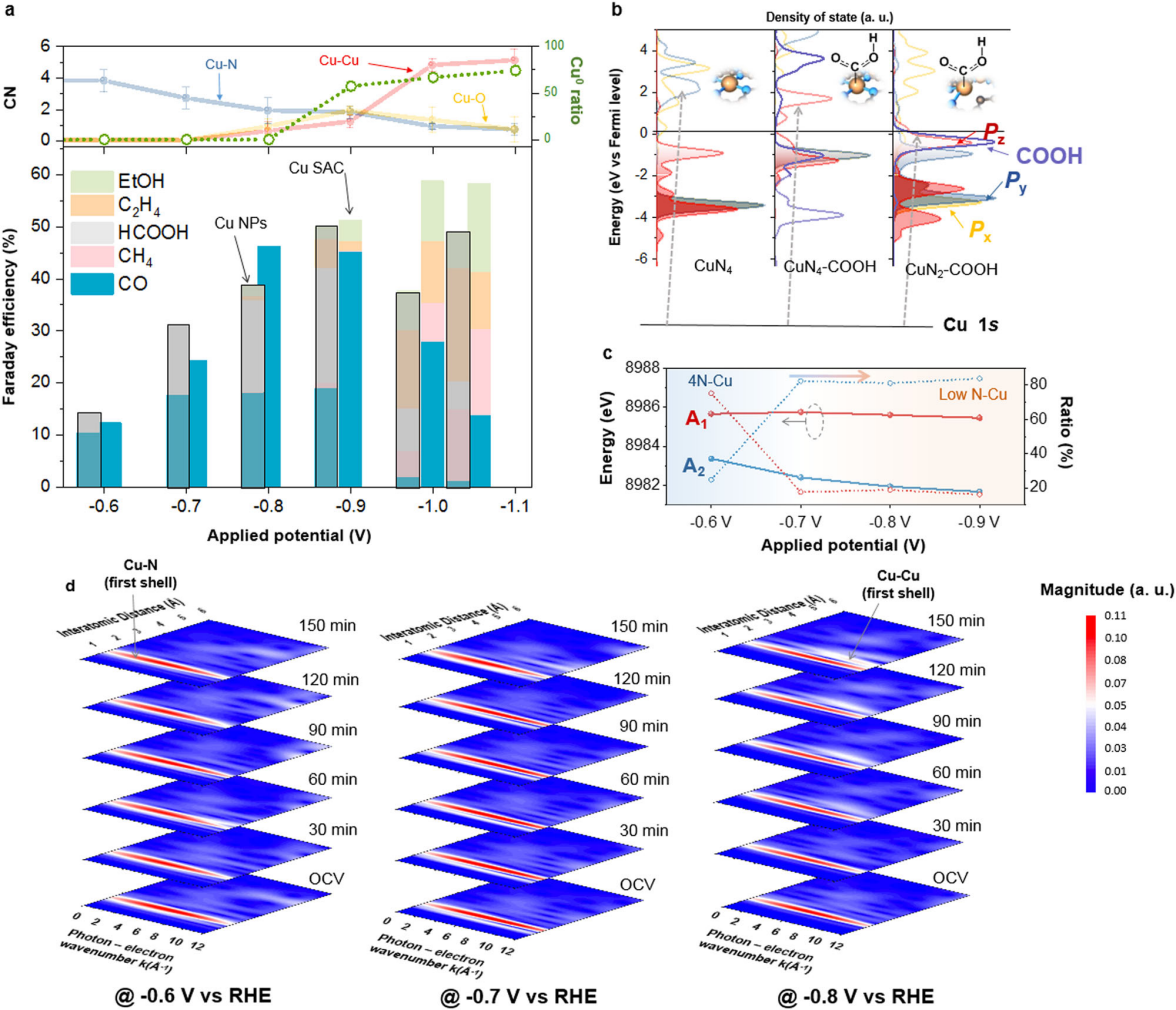
Identification of dynamic configuration-dependent product profile on N–Cu SAC. **a** Potential-dependent product profile on the N–Cu SAC for CO_2_RR and corresponding quantitative analysis extracted from EXAFS fitting at various potentials. Error bars represent the standard deviation of three independent measurements. **b** Calculated projected density of states (PDOS) of Cu *P* orbitals for 4N–Cu SAC, COOH*−4N–Cu SAC and COOH*−2N–Cu SAC. **c** Quantitative analysis of the differential spectra for Cu K-edge HERFD-XAS. **d** Wavelet-transform diagram of in situ Cu K-edge EXAFS spectra of N–Cu SAC at various applied potentials for 150 min in CO_2_-saturated 0.1 M KHCO_3_.

A close correlation between the dynamic restructuring behavior on N-coordinated Cu SAC with quantitative CO_2_RR product profile is further illustrated in Fig. 3a and Supplementary Fig. S17. In addition to the competitive product H_2_, the main product at small cathodic potential region (below −0.8 V vs RHE) is carbon monoxide (CO), which is in accordance with most previous studies^11,13,46–48^. Faradaic efficiency of CO product achieved a maximum value and surpassed 50% at −0.8 V vs RHE. Further increasing the applied potential (−0.9 to −1.0 V vs RHE), intriguingly, some C2 products, such as C_2_H_4_ and C_2_H_5_OH, can be obtained. At even higher potential of −1.1 V vs RHE, significant amount of CH_4_ was revealed to emerge instead of CO production. It has to be pointed out that, at the potential region of forming Cu clusters (i.e., −0.9 V vs RHE and larger cathodic potentials), Faradaic efficiencies toward C2 products significantly increased, which may be ascribed to a mixed-valence of Cu(I) and Cu(0) that substantially manifests a high selectivity for C–C coupling toward ethylene/ethanol^49^ as illustrated in top panel of Fig. 3a. Once applying a larger cathodic potential where a much more complicated restructuring occurred, the restructured Cu catalysts can promote the selective production of methane, which corroborates some reported literature^28,49^. Most interestingly, prior to generating C2 products (below −0.9 V vs RHE), the CO production was observed to be greatly enhanced in Cu SAC with increasing applied potentials. By contrast, in the same potential window, commercial Cu nanoparticles (Cu NPs with 40–60 nm size from Sigma-Aldrich) exhibited a poor CO activity. One can infer that the potential-driven restructuring, i.e., forming a low-coordinated configuration, substantially makes the primary contribution to the improved CO generation in Cu SAC. Findings from both linear sweep voltammetry and cyclic voltammetry also support that the potential-driven restructuring on N-coordinated Cu SAC can significantly boost the resulting current density (Supplementary Figs. S18 and S19). In addition, in situ electrochemical impedance spectroscopy (EIS) results also described distinct behaviors of as-prepared N–Cu SAC and corresponding post-test sample (Supplementary Fig. S20), further validating that the N–Cu SAC underwent structural evolutions from single atom to Cu clusters under CO_2_RR conditions, which are consistent with discussions stated above.

Specifically, the electron injection that enhances the heterogeneity of surface charge^50–52^ may further affect the interplay between the Cu centers and corresponding surface intermediates. It has to be emphasized that, especially for liquid-solid interfaces, the most challenging part is to reveal the intermediates-catalyst interaction and to monitor the reactive sites that are really adsorbing the reaction intermediates. Toward the interfacial interaction between frontier orbitals of metal center and the reactants, an operando HERFD-XAS was utilized by regulating a small incident angle of X-ray and collecting the Kβ_1,3_ emission on the catalytic side, in which the interfacial interaction near the surface can be clearly identified to suppress the intrinsic spectroscopic broadening of core-hole lifetime and instrumental resolution of the final state in conventional in situ XAS (Supplementary Figs. S21–S23)^53–56^. The feature A located between 8979 and 8987 eV was assigned to a transition from Cu 1 *s* to 4*p* state with a simultaneous shakedown transition of ligand electron back into the Cu 3*d* core hole, while the feature B located at approximately 8995 eV was attributed to the transition of 1 *s* to main Cu 4*p* state (Supplementary Fig. S21)^57,58^. To clearly confirm the spectroscopic changes, corresponding differential absorption spectra (Supplementary Fig. S22) and first derivatives spectra (Supplementary Fig. S23) of N-coordinated Cu SAC under various applied potentials exhibited a significantly spectroscopic change of absorbance increase at ~8985 eV at −0.6 V vs RHE (feature A_1_). As revealed by the calculated projected density of states (PDOS) in Fig. 3b (Supplementary Tables S3–S5, see more details in Supplementary Materials), the increase state can be attributed to a generation of additional *P*_z_ contribution, which accounted for the adsorbing of *COOH intermediate in axial direction (*z*-axial)^59,60^. These findings are consistent with the observations in Fig. 3a that the CO production was most likely triggered at −0.6 V vs RHE. At −0.7 V vs RHE and at a larger cathodic potential, an evident split was clarified in addition to feature A_1_, which could arise from the generation of interfacial interaction between low N-coordinated Cu SAC and intermediate because of Cu–N bond-breaking (feature A_2_). The low N-coordinated Cu-*COOH interaction can produce a remarkable increase of *P*_z_ state in an even lower-energy region (Supplementary Fig. S22), which was further validated by the extracted energies and relative ratio of features A_1_ and A_2_ as illustrated in Fig. 3c. Notably, with further increasing the cathodic potential, the feature A_2_ increase showed an evident shift toward low energy region. This phenomenon of energy decrease can be referred to an increased covalency between Cu and reactant^61,62^, suggesting that the Cu–N bond-breaking could offer a stronger covalent bonding with adsorbing reactant (i.e., *COOH). This suggestion was also supported by the PDOS calculation that there is a strong overlapping between the *P*_z_ state and that of *COOH intermediate (Fig. 3b). In addition, in the potential region from −0.7 to −0.8 V vs RHE, the reactive low N–Cu site with adsorbing intermediates can be validated to transform from 4N–Cu configuration to low N–Cu one that is revealed to be ~80% as illustrated in Fig. 3c. Consequently, one can conclude that the intermediates-catalyst interaction is clarified to occur at the potential-driven low N-coordinated configuration which can lead to a substantial interaction and stronger covalency with intermediate than that of the 4N-coordinated case.

### Irreversibility of N-coordinated bond-breaking

In addition to the structural evolution of atomic Cu sites, its reversibility remains unclear. To further investigate the reversibility of Cu SAC, a series of control in situ XAS experiments were employed (Fig. 3d and Supplementary Figs. S24 and S25). In both cases of −0.7 V and −0.8 V vs RHE, the spectroscopic features focusing on the interatomic distance of 1.6 Å gradually decreased, which indicated that the breaking of Cu–N bonds occurred at −0.7 V vs RHE. Another emerging intensive signal focusing at 8.8 Å^−1^ showed that the formation of Cu–Cu bond seemed to happen at −0.8 V vs RHE. It should be noted that the Cu–Cu bonding remained absent at both potentials of −0.6 and −0.7 V even if an extremely long-duration electrolysis more than 150 min was applied. This phenomenon evidently validated that such dynamic restructuring was potential-dependent, regardless of the duration of electrolysis. These findings are also supported by the fate of nitrogen in N–Cu SAC that has been verified by N K-edge XANES, high-resolution XPS and Cu L-edge XANES, in which the coordination of Cu tended to be a pyridinic-type N in as-prepared sample (Supplementary Figs. S26–S28). As compared with the post-catalyst (1-h electrolysis), both N XPS and K-edge XANES manifested a decline in the spectroscopic features related to Cu–N coordination, which may be referred to a Cu–N bond-breaking process (Supplementary Figs. S29 and S30). Observed edge shift from 930 to 929 eV in the Cu L-edge XANES for post-catalyst suggested a change from Cu(II)–N to Cu(II)–O (Supplementary Fig. S31), while the peak at 933 eV, ascribed to Cu(I)–N, remained unchanged after 1 h of electrolysis. Such suggestion can be further supported by the elemental analysis realized by XPS (Supplementary Fig. S32), where the amount of oxygen exhibited significantly increase after 1-h electrolysis, implying that the post-catalyst was an oxide-derived surface instead of metallic one. Furthermore, in situ analysis of Cu K-edge XAS was conducted to reveal that, after removing the applied potential, both the edge position in XANES and the spectroscopic feature in EXAFS showed significant difference from those of the as-prepared sample (Supplementary Fig. S33). It can be verified that the reconstructed Cu clusters are unable to transform back to its original configuration (i.e., N-coordinated single-atom form).

It has to be noted that spectroscopic features in XANES and EXAFS of N–Cu SAC at −0.7 V vs RHE showed evident changes as compared with those at initial state, which have been demonstrated to originate from the formation of low N-coordinated Cu configuration with reduced oxidation state (Supplementary Fig. S34). Once the potential was turned off, both XANES and EXAFS spectra almost reversed to the initial state, however, the spectroscopic features still exhibited some discrepancies relative to those of the initial counterpart. From linear combination analysis of XANES spectra and EXAFS fitting results, it validated that the Cu atoms on post-test catalyst were coordinated by 3N atoms and 1 O atom, in a contrast with the 4N-coordinated Cu configuration (Supplementary Figs. S35 and 36 and Supplementary Table S6). Thus, one can infer that the dynamic breaking of Cu–N bond was partially reversible when the applied potential was released, and the occurrence of Cu–Cu bond formation was clearly irreversible. It’s worthwhile to say that, despite a few studies suggested a reversible restructuring behavior in similar N-coordinated Cu SAC electrocatalysts during CO_2_ reduction^14,15^, the potential-driven restructuring revealed in the present study was evidently irreversible when the Cu cluster formed. This observation was further supported from the aspect of carbon support through in situ Raman spectroscopy. Note that, at the N-doped site, carbon with significant sp^3^ hybridization should contribute to the C–N bond formation^63–66^. As shown in Supplementary Figs. S37–S40, at the potential of −0.8 V vs RHE, a significant decrease in sp^3^ composition indicated the C–N bond-breaking driven by applied potential (as compared with the results of the N-doped graphene and reduced graphene oxide in Supplementary Fig. S37). By a sharp contrast, such significant decrease of sp^3^ ratio in the Fe and Co SACs was not observed, which suggested the well-retained C–N bonding during the CO_2_RR process (Supplementary Figs. S41 and S42), clearly explicating the single-atom nature of Fe and Co SACs without in situ formed clusters. These observations clearly explicate the phenomenon that the forming clusters can freely migrate on the carbon surface because of lacking significant bonding to the carbon support, and the potential-driven electron-injecting may be serving as the driving force for breaking the Cu–N and C–N bonds^61,62,67–69^. To verify this hypothesis, we constructed a model of N-coordinated Cu SAC and employed first-principle DFT calculations to investigate its electronic structures. Supplementary Fig. S43 depicted the orbital energies and calculated frontier molecular orbitals near the Cu center. Evidently, the system exhibited low-lying unoccupied orbitals that were antibonding between the Cu and N atoms (e.g., LUMO + 1 and LUMO + 2). The energies of these orbitals were within the window of varying electrode potentials, therefore we expect that electron injection into these unoccupied orbitals could occur upon cathodic charging conditions. Hence, driven by cathodic potentials, electrons injection into N-coordinated Cu SAC can enhance the heterogeneity of surface charge^50–52^ and further weaken the bonding between Cu centers and coordinated nitrogens, facilitating the restructuring in N-coordinated Cu sites.

### CO production and atomic surface charging

**Fig. 4:**
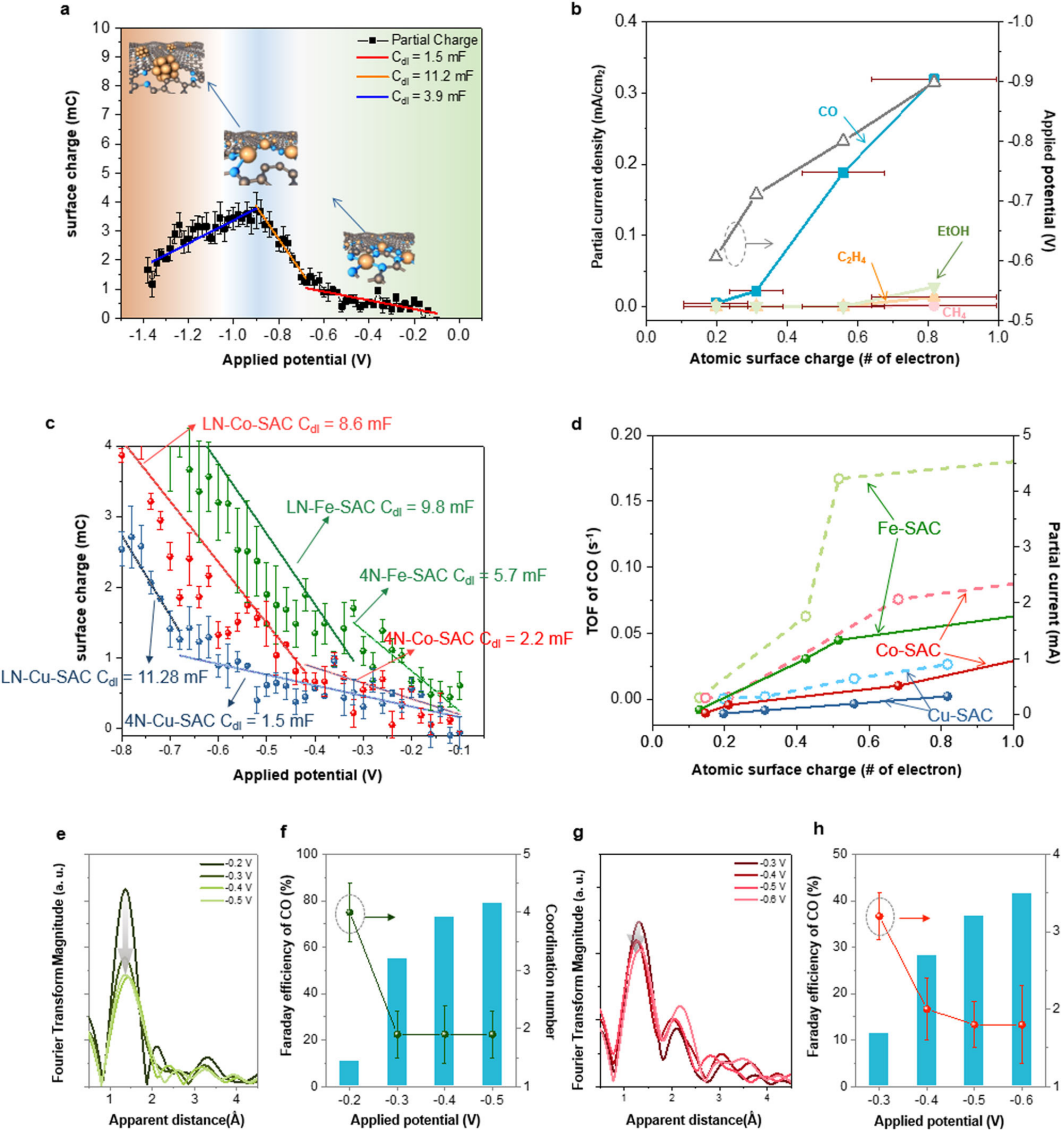
Surface charge analysis and mechanistic understanding. **a** Partial surface charge as a function of applied potentials from the pulse voltammetry of N–Cu SAC. **b** Partial current densities of CO_2_RR products and atomic surface charge as a function of applied potentials for N–Cu SAC. **c** Partial surface charge as a function of applied potentials from the pulse voltammetry of N–Cu SAC, Fe SAC, and Co SAC. LN- represents low N-coordinated metal site. **d** Turnover frequencies (TOF) of CO (dash line) and partial current of CO (solid line) as a function of atomic surface charge for N–Cu SAC, Fe SAC and Co SAC. **e** In situ Fe K-edge EXAFS spectra of 4N-Fe(II) SAC and **g** in situ Co K-edge EXAFS spectra of 4N-Co(II) SAC at various applied potentials during CO_2_ reduction. Potential-dependent CO_2_RR product profile and corresponding coordination number extracted from EXAFS fitting on the **f** 4N-Fe(II) SAC and **h** 4N-Co(II) SAC at various potentials. Error bars represent the standard deviation of three independent measurements.

Such potential-driven generation of dynamic low-coordinated configuration can account for a general observation that, in most studies of single-atom catalysts, the activities toward CO production were manifested to a significant enhancement at small cathodic potential regions regardless of the small increase in applied potential^9,11,12,46,70,71^ To account for the intrinsic activity toward CO production, electron storage in 4N-coordinated Cu SAC and low-coordinated one during CO_2_RR may play a paramount role in catalyzing CO generation. A pulse voltammetry was employed to quantify the charge stored in the catalyst at a given potential (Supplementary Fig. S44)^53^. By integrating the current response to the anodic voltage pulses (Supplementary Figs. S45 and S46), it provides access to the relationships between stored surface charge and applied potentials. To exclude the contributions from the carbon substrate, the surface charge associated exclusively to the metal atoms can be obtained through subtracting the total surface charge of SACs by that of the N–C substrate (Supplementary Fig. S47). As shown in Fig. 4a, apparently, the charge distribution on Cu SAC is characteristic of a three-region feature as a function of applied potentials, where slope changes at −0.7 and −0.9 V vs RHE are yielded with the double-layer capacitance (C_dl_) increasing by 9.7 mF and decreasing by 7.3 mF, respectively, indicating that the 4N-coordinated configuration gradually evolves to low-coordinated configuration and then Cu clusters with increasing the applied potentials. Quantitatively, the capacitance values can be employed to estimate atomic surface charge (*φ*_e_), in which *q*_*e*_ (elementary charge) is the electric charge carried by a single electron (see more details in Supplementary Materials).

$${{{{{\rm{atomic}}}}}}\; {{{{{\rm{surface}}}}}}\; {{{{{\rm{charge}}}}}}\,\left({\varphi }_{e}\right)=\,\frac{{{{{{\rm{surface}}}}}}\; {{{{{\rm{charge}}}}}}\,(\theta )}{{{{{{\rm{Cu}}}}}}\; {{{{{\rm{atoms}}}}}}\; {{{{{\rm{on}}}}}}\; {{{{{\rm{electrode}}}}}}\cdot {{{{{\rm{elementary}}}}}}\; {{{{{\rm{charge}}}}}}\,({q}_{e})}$$

The correlation of the partial current densities of various products versus the surface charge per atom (*φ*_e_) shown in Fig. 4b exhibits that, below −0.7 V vs RHE, the CO product is inactive with atomic surface charge of about 0.2–0.3 electron per Cu atom while the CO production increases linearly with atomic surface charge increasing at above −0.7 V vs RHE. Notably, as compared with that at −0.6 V vs RHE, the partial current density of CO production increases about 50 times at −0.8 V vs RHE with the increase of the atomic surface charge to ~0.5 electron per Cu atom, which happens to coincide well with the atomic-configuration transformation from 4N- to low N-coordinated Cu sites that can possess less electron-sharing with adjacent nitrogen and allow to transfer more electron charge than that of 4N-coordinated one. In the case of catalysts with forming a few Cu clusters at −0.9 V vs RHE, the atomic surface charge was approximately determined to be above 0.8 electron per Cu atom, which indicates the commence of executing the multi-electron transferring for C2 products.

To further evidence our proposition, we also synthesized three N-coordinated Cu SAC (3N–Cu SAC), and its surface charge and CO_2_RR selectivity have been evaluated (Supplementary Figs. S48–S51). The double-layer capacitance of 3N–Cu SAC remains at 4.1 mF at the potential ranging from −0.5 to −0.8 V, which implies the relatively stable structure of such low-coordinated Cu SAC. Significantly, the atomic surface charge per Cu atom on 3N–Cu SAC was determined to be 0.5, which is consistent with the value that explicates a 50-fold enhancement of CO activity on the low N-coordinated Cu sites in N–Cu SAC. Such results reveal that atomic surface charge might act as an informative indicator for governing the CO_2_RR product profile, which can be further supported by atomically dispersed Fe and Co SACs with 4N-coordinated configurations (Supplementary Figs. S52–S58). It can be clearly seen from Fig. 4c that, over the applied potential range (−0.1 to −0.8 V vs RHE), the charge distribution of Fe and Co SACs are characteristic of bilinear features with potentials. The double-layer capacitance changes from 5.7 to 9.8 mF for Fe SAC at −0.3 V vs RHE, while that of Co SAC alters from 2.2 to 8.6 mF at -0.4 V vs RHE. The slope changes occur at the same potential as the atomic-configuration transformation in these SACs, which can be further validated by in situ XANES and EXAFS analyses (Fig. 4e–h, Supplementary Figs. S59–68 and Supplementary Tables S7–S9). Apparently, CO FE consistently exhibits a strong correlation with the decline in coordination number of Fe/Co-N path (Supplementary Fig. S69). By contrast, one can further see that the poorly active Zn SAC did not behave in the same way as Fe, Co and Cu SACs, in which the coordination number of Zn-N and atomic surface charge almost remained unchanged over the whole CO_2_RR course, exhibiting the ultra-low performance for CO_2_-to-CO conversion (Supplementary Fig. S70). These results indicate the universality of the important role played by the dynamic low-coordinated configuration of the single-atom electrocatalysts for CO production during CO_2_RR (Supplementary Figs. S71–S73). It has to be noticed that, for Cu, Fe and Co SACs, their turnover frequencies (TOF) and partial current of CO are both strongly in accordance with the calculated atomic charge per atom (*φ*_e_) as shown in Fig. 4d. Specifically, all the SACs were inactive for the CO production with the atomic surface charge below ~0.3 electron per metal atom. Once the atomic surface charge exceeded ~0.3 electron per atom, we found that that both the TOF and partial current of CO on the SACs started to increase remarkably. Especially, when the atomic surface charge increased to ~0.5 electron per atom, both aspects exhibited the most remarkable enhancement. These results indicated that the potential-driven low N-coordinated configuration on SAC with corresponding surface charge could effectively trigger the selectivity and activity for CO_2_-to-CO conversion. However, we can also find that the absolute selectivity and activity of various SACs are mainly dominated by the nature of specific metals. For instance, atomically dispersed Fe catalyst generally shows much higher CO selectivity and activity at more positive potentials as compared with Cu SACs^11,46,72–74^. Based on these analyses, the atomic surface charge can act as an informative indicator for selectivity and activity toward the CO_2_RR product profile. The most important finding is a universal explication that low-coordinated configuration is verified to be the reactive form to catalyze CO_2_ electroreduction toward CO.

## Discussion

By tracking the dynamic configuration changes of atomically dispersed electrocatalyst, the mechanism behind the atomic-configuration-dependent CO_2_RR performance is illustrated in Supplementary Fig. S74. First, at −0.6 V vs RHE, the Cu sites that remain in the origin 4N-coordinated structure exhibit a low activity toward CO production since the intermediate adsorbing can be still observed by HERFD-XAS. Once the applied potential reaches to −0.7 V vs RHE, accompanying with injecting electron into the antibonding orbitals of the Cu–N bond, the potential-driven bond weakening/breaking can result in formation of the low-coordinated Cu configuration with boosting atomic surface charge, which greatly adapts the frontier orbitals to facilitate the covalency and reactant adsorbing, remarkably enhancing the generation toward CO. Impressively, the CO production observed in the low N-coordinated Cu configuration at −0.8 V vs RHE is almost a fifty times higher than that in the 4N-coordinated counterpart. As the applied potential reaches at −0.9 V vs RHE, accompanying with the further breaking of C–N bonds on carbon support, the generated Cu atoms can randomly move on the surface to form Cu clusters with surface hydroxyl stabilization. At −1.0 V vs RHE and even larger potentials, more Cu atoms detach from the support and aggregate to form small clusters with a critical size of approximate 3 nm in final stage. Notably, even at larger cathodic potentials, the size of Cu clusters is still steady without enlarging to form larger nanoparticles. This may be attributed to the capping effects as a consequence of increase in potential-driven local pH value for mitigating the cluster growth^44,45^. At this stage, the electrochemical behavior of the resultant catalyst during CO_2_ reduction is more similar to that of reported Cu nanoparticles/nanostructures^75^, in which the main product would be C2 product (i.e., C_2_H_4_ and C_2_H_5_OH) and methane in high cathodic potentials^46,75,76^. These results can be further validated by potassium thiocyanate (KSCN) poisoning experiments on the N–Cu SAC during CO_2_RR (Supplementary Fig. S75). It has to be noticed that the generated metallic Cu clusters is unable to reversibly transform back to N-coordinated Cu SAC. As demonstrated in Supplementary Fig. S31, the resulting metallic Cu would be slightly oxidized to form Cu oxide instead of N-coordinated Cu SAC because of the capping effect stated above.

The potential-driven dynamic restructuring was directly revealed on the atomically dispersed copper system, which further elucidates the configuration-dependent product profile for its electrochemical CO_2_ reduction. To be specific, the imaging of the dynamic changes in N-coordinated Cu single-atom electrocatalysts during CO_2_ reduction was firstly realized through liquid electrochemical TEM, and based on comprehensive analyses, a dynamic activation on atomic configuration was found to generate adaptive low-coordination form (the metal–N bond weakening/breaking involved during CO_2_RR) with efficient atomic surface charging for a significantly higher activity toward CO product over the well-known four N-coordinated configuration. It has been demonstrated that the atomic configuration of SACs can significantly affect the electronic structure of active sites and the adsorption of intermediates, which would finally change the energetics of the CO_2_RR pathways^77,78^. Since the debate regarding the role of dynamic Cu SAC in CO_2_RR process has been ongoing for years, we believe that the point of view raised in this study, dynamic low N-coordinated configuration with storing charges on atom surface to stimulate the CO_2_RR, would offer a universal explication for single-atom catalysts systems and provide a model of comprehensive in situ/operando investigations on the mechanistic understanding. Our findings underscore the significant potential of atomic surface charge as an informative indicator for establishing correlations with electrocatalytic activity^53,79^. Therefore, the alterations in atomic surface charge during electrocatalysis should garner considerable attention and undergo extensive investigations within the scientific community. By conducting additional validation studies on diverse electrocatalytic systems and advancing more precise measurement techniques for surface charge, we anticipate that atomic surface charge will emerge as a highly influential indicator for comprehending electrocatalytic processes and discovering efficient catalysts.

## Methods

### Synthesis of metal single-atom catalyst

In total, 3 g of melamine, 0.5625 g of l-alanine (C_3_H_7_NO_2_) and 0.05 g of metal acetate (Fe(CH_3_COO)_2_, Co(CH_3_COO)_2_, Cu(CH_3_COO)_2_·H_2_O) were first dissolved in 50 mL of water. The resulted mixture was heated overnight at 80 °C in an oil bath, and a solid precipitate was obtained. Subsequently, the fine-powdered mixture underwent a two-stage pyrolysis and carbonization process (first stage: from 25 to 600 °C at a ramping rate of 3 °C min^–1^, maintained at 600 °C for 2 h; second stage: from 600 to 900 °C at a ramping rate of 2 °C min^–1^, maintained at 900 °C for 1 h) in a tubular furnace in argon atmosphere. After cooling down to room temperature, the product was then leached at 80 °C in 1 M HNO_3_ for 24 h in order to remove metal particles and unstable species. Notably, during the acid leaching process, the structure of graphene could be broken and/or amorphization, which thus requires the second pyrolysis process (850 °C in Ar for 1 h) to recover the crystallinity of carbon for electrocatalysis. For comparison, N–C catalyst was prepared with the same method as metal SAC except that no metal precursor was added.

### Synthesis of Zn single-atom catalyst

The synthesis of Zn single-atom catalyst followed reported synthetic procedures in our recent work^8^. 1.314 g of 2-methylimidazole and 0.076 g of KI were dissolved in 20 mL of methanol as solution A, 1.190 g of Zn(NO_3_)_2_·6H_2_O was dissolved in the same volume of methanol as solution B. The solution B was then added into solution A drop by drop under stirring. The solution mixture was kept under stirring for 1 day under an N_2_ atmosphere, after that the precipitate was separated by ambient-pressure filtration and washed with methanol for three times. The precipitate was dried at room temperature under vacuum. The obtained white powder was placed in a corundum crucible and immersed by hexane, then transferred into a tube furnace with an N_2_ flow (100 mL min^−1^). The program of pyrolysis is: from 25 °C to 120 °C with a ramping rate of 1 °C min^−1^ for the evaporation of hexane; from 120 °C to 900 °C with a ramping rate of 5 °C min^−1^; kept at 900 °C for 3 h. After natural cooling, Zn single-atom catalyst was obtained as a black powder.

### Synthesis of 3N–Cu SAC

In total, 12.5 mmole of Cu(NO_3_)_2_ and 0.5 g melamine were first dissolved in 10 mL of DMSO as solution A. 0.51 g cyanuric acid was dissolved in 10 mL of DMSO as solution B. The solution B was added into solution A dropwise under stirring for 10 min. The green solution momentarily became blue as solution B was added, and white precipitate was formed subsequently. The solution would be filtered by 150 mL DI water and 100 mL ethanol. After drying at 60 °C for 12 h, the powdered precursor was pyrolyzed at 550 °C under N_2_ atmosphere for 4 h in a tube furnace at a ramp rate of 2.3 °C min^−1^.

### Characterization

The HR-TEM image and dark-field STEM image of the catalyst were collected by using transmission electron microscopy (TEM) of JEOL JEM-2100F microscope with a field-emission gun source. The high-angle annular dark-field scanning TEM (HAADF-STEM) images and STEM-EDX elemental mapping were collected on a high-resolution transmission electron microscopy (JEM-ARM200F working at 300 kV), equipped with a probe spherical aberration corrector. The crystalline structure analysis was carried out by X-ray diffraction (XRD, Bruker D2 Phaser) with Cu Kα radiation (*λ* = 1.54 Å). The detected angle ranged from 10° to 80° for 1 s and the interval step was 0.01°. The XPS analysis was performed with a PHI 5000 Versa Probe (ULVAC-PHI, Japan) system, using a monochromatic Al Kα X-ray source. In order to avoid surface potential build-up during the measurement, all spectra were acquired while the sample surface was neutralized by an e^−^ and an Ar^+^ beam with an acceleration voltage of 10 V. ICP-MASS result was obtained by an iCAP-RQ instrument (Thermo Fisher). For sample preparation, the 1 mg of the sample was dissolved in 2 wt% HNO_3_ by an ultrasonic treatment. Notably, the concentration was kept less than 50 ppb. The amount of Cu atom was estimated by the calibration curve of Cu standard.

For in situ TEM measurement, N–Cu SAC samples were first dispersed in ethanol, and the suspension was deposited directly onto an E-chip that was coated with three Pt electrodes by Protochip. A second E-chip window was then placed on the bottom of the first one in the Poseidon select holder, creating a thin liquid cavity sealed by O-rings from the high vacuum of the TEM column. The space between two SiNx membranes that are characteristic thickness was 0.5-${{{{{\rm{\mu }}}}}}{{{{{\rm{m}}}}}}$. Note that the electrolyte was injected into the observing window at a flow rate of 300 μL h^−1^, which prevents the product bubbles accumulating on the electrode during the CO_2_RR. In situ electron microscopy was performed with a JEOL JEM-2100F transmission electron microscope equipped with a 200 kV cold field-emission gun. The electron beam was turned off for most of the time except for image collection and the dose rate of electron beam was 0.3 e^−^/Å^2^ during collecting the image. The electrochemical characterization during the image collection was by GAMRY reference 600. The scanning rate of each image was 16 μs. The measurement in a typical three-electrode setup was performed in 0.1 M KHCO_3_ by Poseidon select holder, which used Pt electrodes as all the working, counter, and reference electrodes. All the applied potential was calibrated to reversible hydrogen electrode (RHE). Given that the primary product of CO_2_RR on SACs is CO, the CO poisoning effect on the open-circuit potential drift during reactions must be taken into account since CO is a strong poison for Pt surfaces. To correctly calibrate Pt quasi-reference electrode to its RHE, we used “HydroFlex Standard Hydrogen Reference Electrode” as reference electrode for the calibration process (Supplementary Fig. S76 and 77, see details in Supplementary Materials).

The XAS data were collected in total-fluorescence mode, which recorded at beamline 12B of Taiwan beamline at Spring-8 of NSRRC. The electron storage ring was operated at 8.0 GeV with a constant current of ~400 mA. The incident beam energy was monochromatized by a Si (111) double-crystal monochromator. The scan range was kept in an energy range of 8950–9850 eV for Cu K-edge. The data collected were normalized to the incoming incident photon flux and processed with the Athena software from the IFEFFIT package. E_0_ values of 8979.0 eV were used to calibrate all data with respect to the first inflection point of the absorption K-edge of iron foil. EXAFS analysis was conducted using Fourier transform on k^3^-weighted EXAFS oscillations to evaluate the contribution of each bond pair to Fourier transform peak. X-ray absorption spectra of N K-edge and Cu L-edge were collected through total electron yield at beamline 20 A of National Synchrotron Radiation Research Center (NSRRC) in Taiwan. The electron storage ring was operated at 1.5 GeV with a constant current of ~360 mA. The incident beam energy was monochromatized by grating. The scan range was kept in an energy range of 389−428 eV for N K-edge and 918–972 eV for Cu L-edge. For in situ XAS experiments, measurement in a typical three-electrode setup using glassy carbon as the working electrode, Hg/HgCl_2_ electrode and platinum plate acted as reference and counter electrodes, respectively, was performed in 0.1 M KHCO_3_ by a specially designed Teflon container with a window sealed by Kapton tape. All the applied potential was calibrated to reversible hydrogen electrode (RHE). The incident beam energy was monochromatized by a Si (111) double-crystal monochromator with the energy resolution of 1 eV. X-ray was allowed to transmit through the tape and electrolyte, so that the signal of X-ray absorption spectroscopy could be collected in total-fluorescence-yield mode by Lytle detector in BL-12B of Taiwan beamline in Spring-8.

In situ XES measurement in a typical three-electrode setup using glassy carbon as the working electrode, Hg/HgCl_2_ electrode and platinum plate acted as reference and counter electrodes, respectively, was performed in 0.1 M KHCO_3_ by a specially designed Teflon container with a window sealed by Kapton tape. All the applied potential was calibrated to reversible hydrogen electrode (RHE). The incident beam energy that was at constant energy of 9000 eV was monochromatized by a Si (111) double-crystal monochromator and was allowed to transmit through the tape and electrolyte at 10° of incident angle, and the fluorescence was split by the analyzer crystal Si(444), collecting signals by silicon drift detector (XR-100CR Si-PIN X-ray detector) in the scan range of 8020-8070 eV in BL-12XU at SPring-8.

In situ HERFD measurement in a typical three-electrode setup a using glassy carbon as the working electrode, Hg/HgCl_2_ electrode and platinum plate acted as reference and counter electrodes, respectively, was performed in 0.1 M KHCO_3_ by a specially designed Teflon container with a window sealed by Kapton tape. All the applied potential was calibrated to reversible hydrogen electrode (RHE). The incident beam energy was monochromatized by a Si (111) double-crystal monochromator and four-bounce channel cut Si(440) (HRM) was also inserted for enhancing the energy resolution to reach 0.3 eV. The scan range was kept in an energy range of 8960-9000 eV for Cu K-edge. X-ray was allowed to transmit through the tape and electrolyte at 10° of incident angle, and the fluorescence was split by the analyzer crystal Si(444), so that the signal would be collected by silicon drift detector (XR-100CR Si-PIN X-ray detector) in partial-fluorescence-yield mode in BL-12XU at SPring-8.

In situ Raman measurement in a typical three-electrode setup using glassy carbon as the working electrode, Hg/HgCl_2_ electrode and platinum plate acted as reference and counter electrodes, respectively was performed in 0.1 M KHCO_3_ by a specially designed container. All the applied potential was calibrated to reversible hydrogen electrode (RHE). In situ Raman spectra were recorded by UniNano UNIDRON―employing a diode laser at 532 nm. A ×50 objective lens was used to focus the laser on the sample, in which the size of laser spot is 1 μm. The Raman spectrum measurement was performed for an exposure time of 2 s and an accumulation number of five times by illuminating 2.5 mW laser power.

### Electrochemical measurement

All electrochemical characterization was conducted by BioLogic VSP in a standard three-electrode configuration cell using glassy carbon as the working electrode, Hg/HgCl_2_ electrode and platinum plate acted as reference and counter electrodes, respectively. In the case of working electrode preparation, the electrocatalysts were dispersed in ethanol (1 mg/mL) with an addition of 10 μL 5% Nafion solution, which was then dropped (20 μL) onto glassy carbon. Electrochemical experiments and impedance spectra were performed in 1 M CO_2_-saturated KHCO_3_ solution as electrolyte. The potential was calibrated with ohmic loss (*R*_*u*_) with respect to reversible hydrogen electrode (RHE), *E*_RHE_ = *E*_ref_ + 0.05916×*pH*-*i*×*R*_*u*_. Pulse voltammetry (PV) were performed at a low potential (*E*_*l*_ = 0 V) for 30 s, then switched at a higher potential (*E*_*h*_) for 30 s before returning to *E*_*l*_ for 30 s. This cycle was repeated while increasing *E*_*h*_ from −0.1 to −1.4 V in 100 mV/step and keep *E*_*l*_ unchanged. The current was collected every 0.005 s for the cathodic sections. The charge was calculated by integrating the current pulse over time accounting for the background current signal, which refer to each *E*_*h*_ applied. ICP-MS measured the metal loading of various single-atom catalysts. The loading amount of metal was calculated to be 0.83, 0.62, 0.38, 0.45, and 0.42 mmol/mg for N–Cu SAC, 3N–Cu SAC, Co SAC, Fe SAC, and Zn SAC, respectively. Thus, the charge per atom can be estimated at each potential.

For single-atom catalysts, each metal site was regarded as a catalytic site. Then, the apparent turnover frequency (TOF) of CO formation was calculated according to *TOF*_CO_ = *j*_CO_/(2*e*×*m*×*S*×*d*), where *j*_CO_ is the partial current density of CO; *e* is the charge of an electron (1.6 × 10^−19^ C); *m* is the loading of the metal (unit: mg cm^−2^); *S* is the specific surface area of the catalyst (m^2^ g^−1^) and *d* is the estimated atomic density on the metal surface (cm^−2^).

### CO_2_ reduction product analysis

A homemade two-chamber H-type cell made of polymethylmethacrylate was used. Three-electrode configuration cell used glassy carbon as the working electrode, Hg/HgCl_2_ electrode and platinum plate acted as reference and counter electrodes, respectively. Working and reference electrodes were fixed in one chamber and the counter electrode was fixed in the other chamber. The two chambers were separated by an anion exchange membrane (Fumasep FAB-PK-130) to avoid any possible contamination from the Pt electrode. Product distribution analysis was conducted after 10 C of charge collected under constant potential. Gas products were analyzed by gas chromatography (Agilent 7890 A, Agilent Technologies) with a thermal conductivity detector (TCD) and a flame ionization detector (FID) in series. The TCD was for CO detection, and the FID was for CH_4_ and C_2_H_4_ detection. As for those products in liquid phase, gas chromatography-mass spectrometry (GC-MS, 5977 A) was adopted to detect ethanol. All the calibration curves of CO_2_ reduction products are showed in Supplementary Fig. S78. Formate were analyzed by NMR (Bruker AVIII HD−400 MHz NMR).

### Computational details

The Gaussian 09 program was employed in all calculations presented in this study. The molecular geometry of each CO_2_ reduction intermediate was optimized at the B3LYP- D3(BJ) level, and we confirmed that there was no imaginary frequency for all optimized geometries by using frequency analysis calculations. We utilized B3LYP hybrid function as the main DFT function and D3 version of Grimme’s dispersion with Becke-Johnson damping for dispersion correction, because DFT methods with empirical dispersion correction have been shown to provide a better description of the metal-ligand and π-conjugated interactions. For all the calculations, the 6-31 G(d,p) basis set was used for the H, C, N, and O atoms, and the effective core potential LANL2DZ basis set was used for Cu atom to investigate the optimized geometries and potential energy surfaces.

### Supplementary information

Supplementary Information

Description of Additional Supplementary Files

Supplementary Movie 1

Supplementary Movie 2

Supplementary Movie 3

Supplementary Movie 4

Supplementary Movie 5

Supplementary Movie 6

## Data Availability

The data supporting the conclusions of this study are present in the paper and the Supplementary Information. The raw datasets used for the presented analysis within the current study are available from the corresponding authors upon request.
