# Supplementary material for: Activating dynamic atomic-configuration for single-site electrocatalyst in electrochemical CO2 reduction
Source: Nat Commun. 2023 Aug 28;14:5245. doi: 10.1038/s41467-023-40970-y (PMC10462635; doi:10.1038/s41467-023-40970-y)
Supplement: Supplementary file 1 — Supplementary Information [file 41467_2023_40970_MOESM1_ESM.pdf]

Activating dynamic atomic-configuration for single-site electrocatalyst in electrochemical CO_2 reduction

Chia-Shuo Hsu,[1,7] Jiali Wang,[1,7] You-Chiuan Chu,[1] Jui-Hsien Chen,[1] Chia-Ying Chien,[1] Kuo-Hsin Lin,[2] Li Duan Tsai,[2] Hsiao-Chien Chen,[3] Yen-Fa Liao,[4] Nozomu Hiraoka,[5] Yuan-Chung Cheng[1]* and Hao Ming Chen[1,4,6]*

[1]*Department of Chemistry, National Taiwan University, Taipei 10617, Taiwan*

[2]*Material and Chemical Research Laboratories, Industrial Technology Research Institute, Chutung, Hsinchu, 31040, Taiwan*

[3]*Center for Reliability Sciences and Technologies, Chang Gung University, Taoyuan 333, Taiwan*

[4]*National Synchrotron Radiation Research Center, Hsinchu 30076, Taiwan*

[5]*Japan Synchrotron Radiation Research Institute, 1-1-1 Kouto, Sayo, Hyogo 689-5198, Japan*

[6]*Graduate Institute of Nanomedicine and Medical Engineering, College of Biomedical Engineering, Taipei Medical University, Taipei 11031, Taiwan*

[7]*These authors contributed equally*

Email: yuanchung@ntu.edu.tw (Y.C.C.); haomingchen@ntu.edu.tw (H.M.C.)

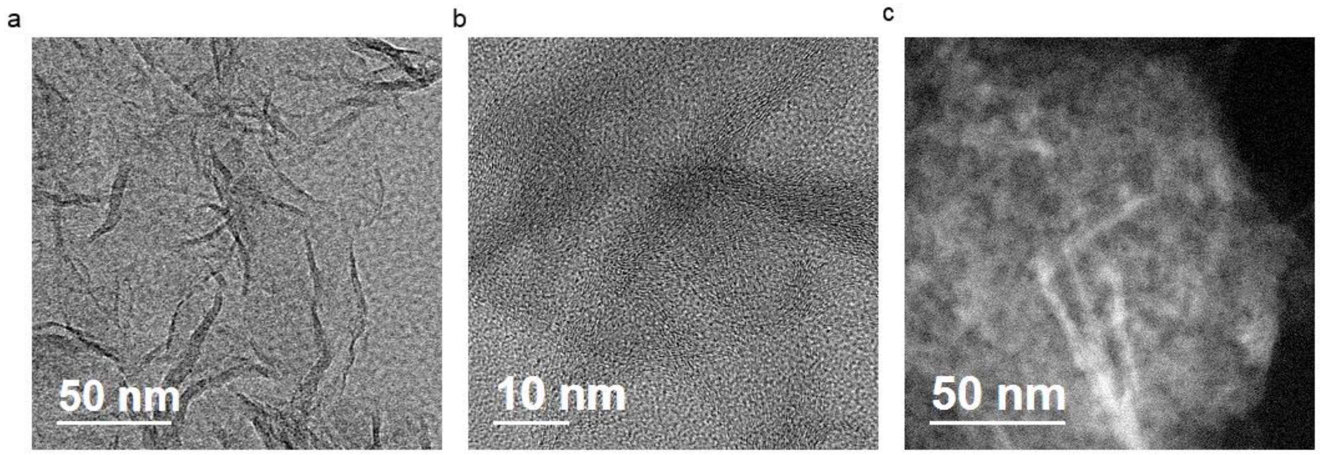

Figure S1. Structural characterization of N-Cu SAC. (a, b) HR-TEM images of N-Cu SAC. (c) Dark-field STEM image of N-Cu SAC.

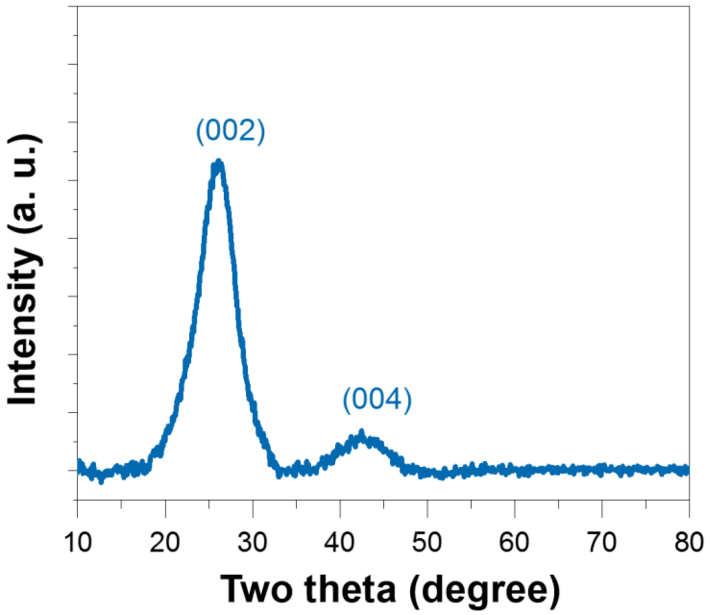

Figure S2. XRD analysis of N-Cu SAC. XRD pattern of N-Cu SAC, showing solely two characteristic peaks at 26.2° and 44.0°, which are attributed to the (002) and (004) planes of carbon, respectively, suggesting that no metallic Cu nanoparticles were formed throughout the process.

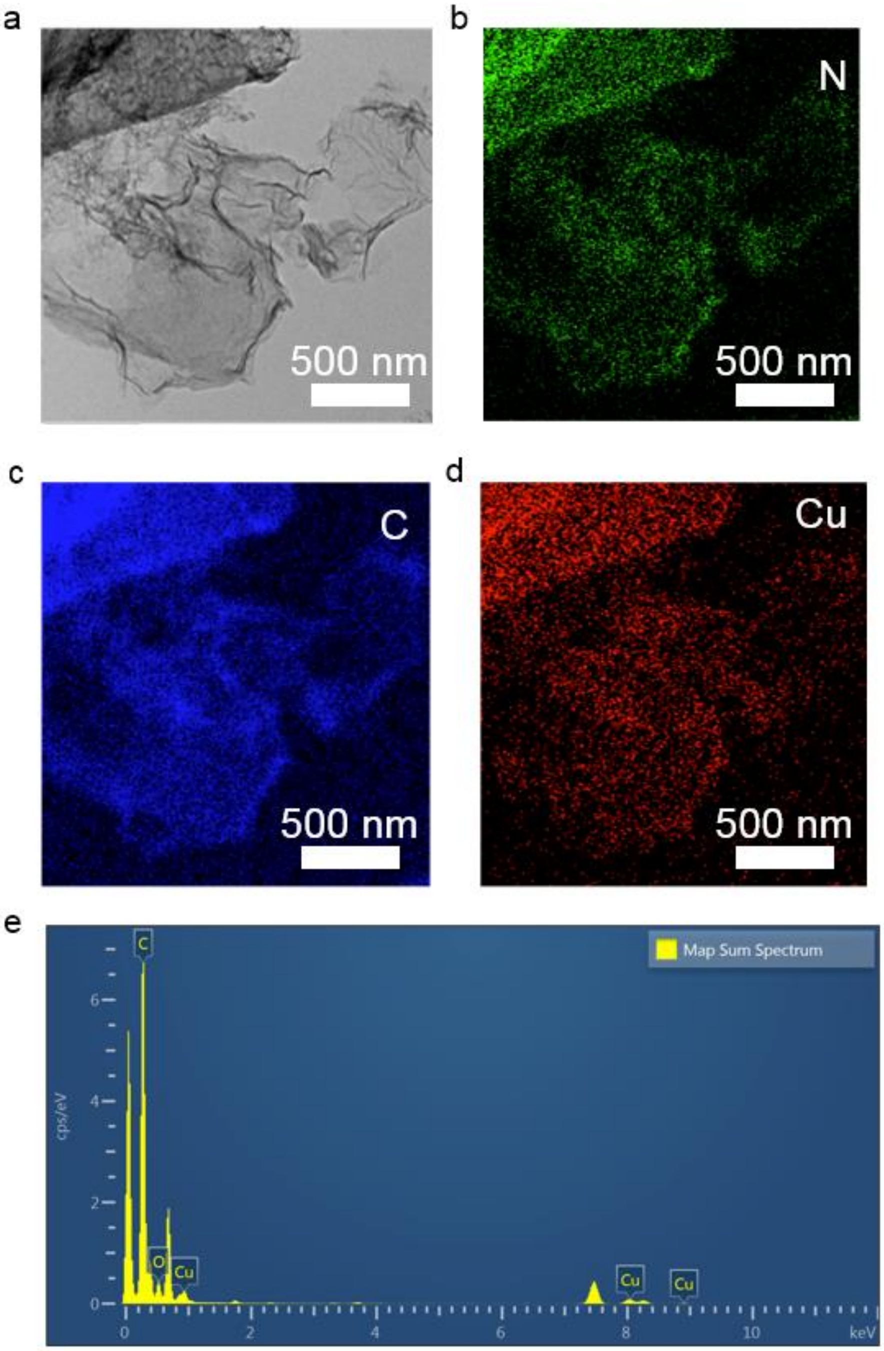

Figure S3. Morphology characterization and elemental analysis of N-Cu SAC. (a) STEM image, (b-d) corresponding EDX mapping images and (e) EDX spectrum of N-Cu SAC.

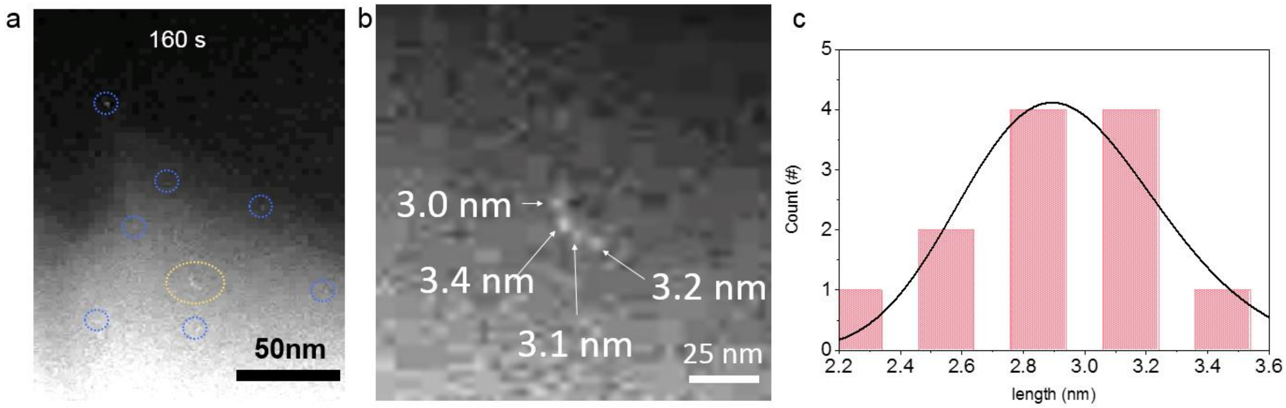

Figure S4. Structural characterization of N-Cu SAC during CO_2RR. (a) Dark field STEM image at applied potential of -1.1 V vs RHE for 160 s. (b) Dark-field STEM image of the selected region of (a). (c) The size distribution of formed particles in (a).

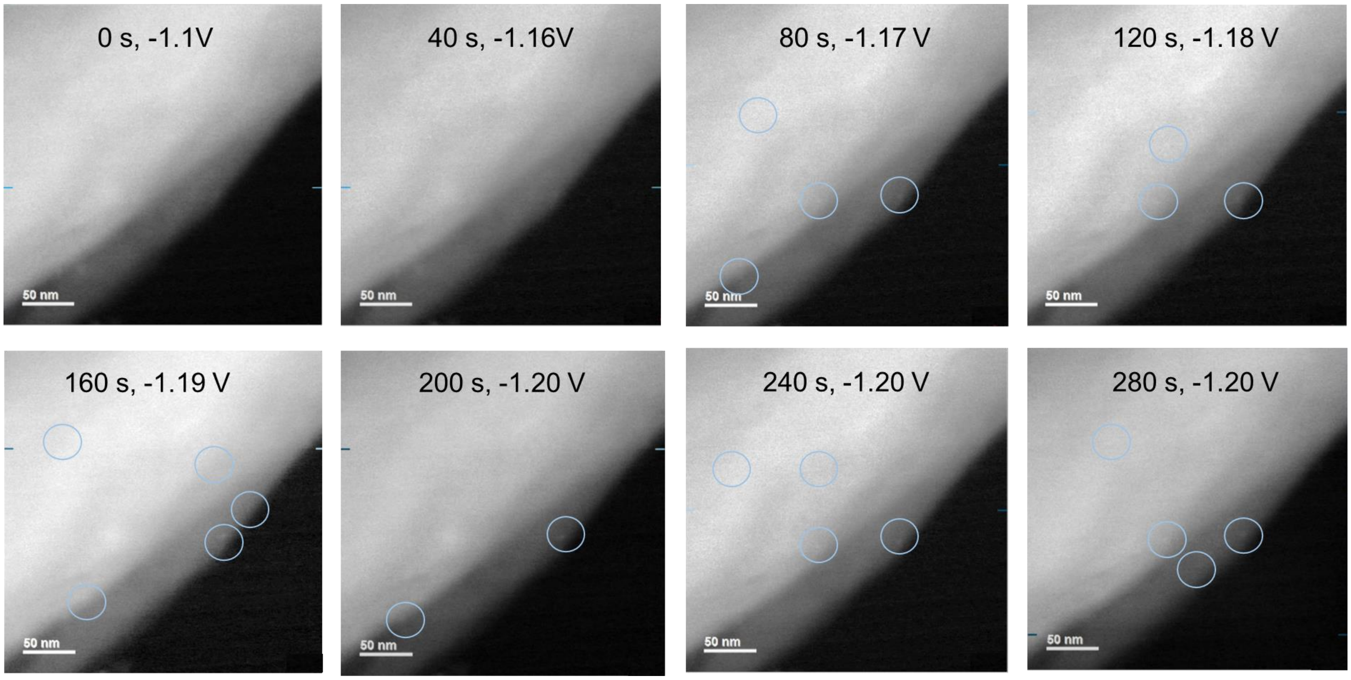

Figure S5. Liquid electrochemical TEM analysis of N-Cu SAC. Dark field STEM images at truly calibrated potentials vs RHE with various duration from 0 to 280 s.

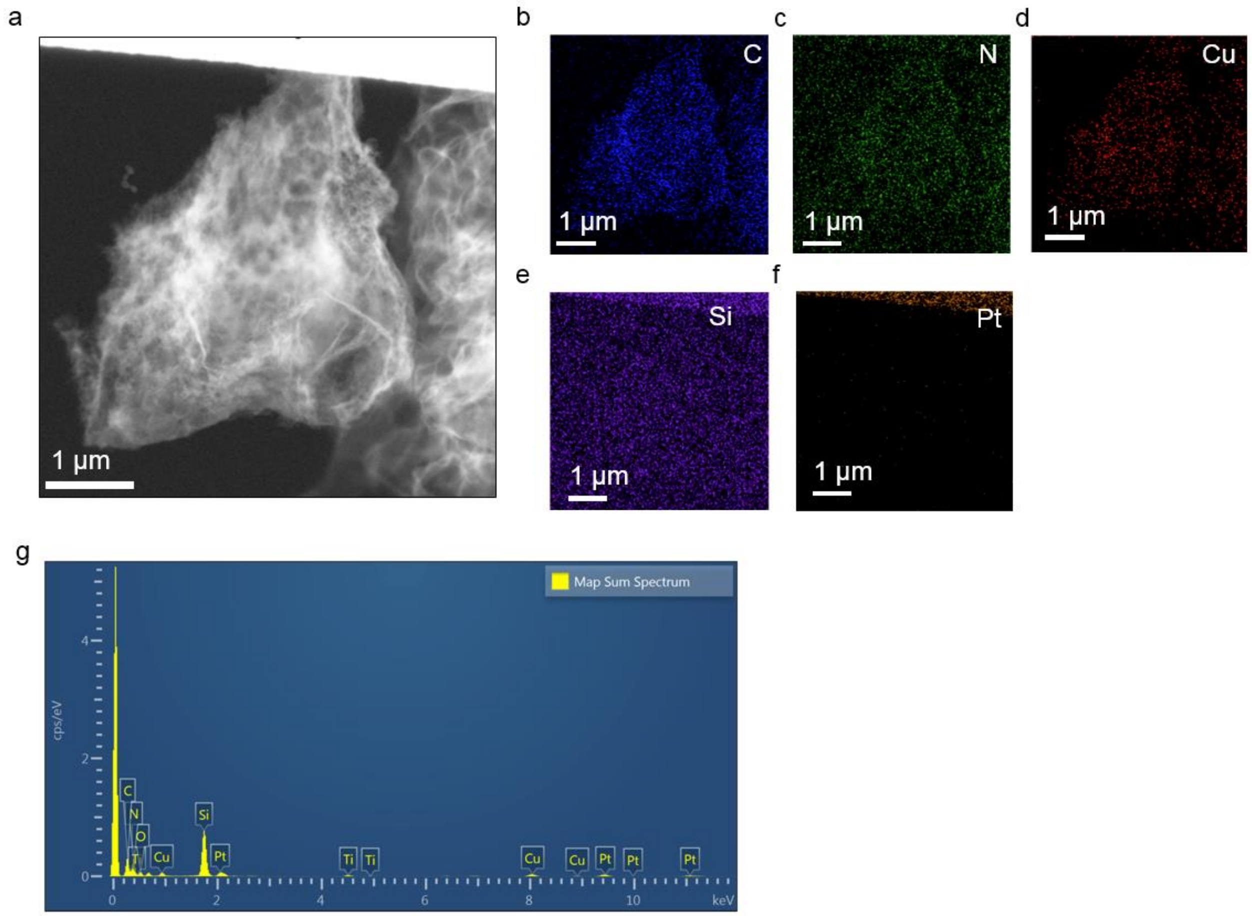

Figure S6. Physical characterization of N-Cu SAC during CO_2RR. (a) STEM image, (b-f) corresponding EDX mapping images and (g) EDX spectrum of N-Cu SAC on Pt working electrode.

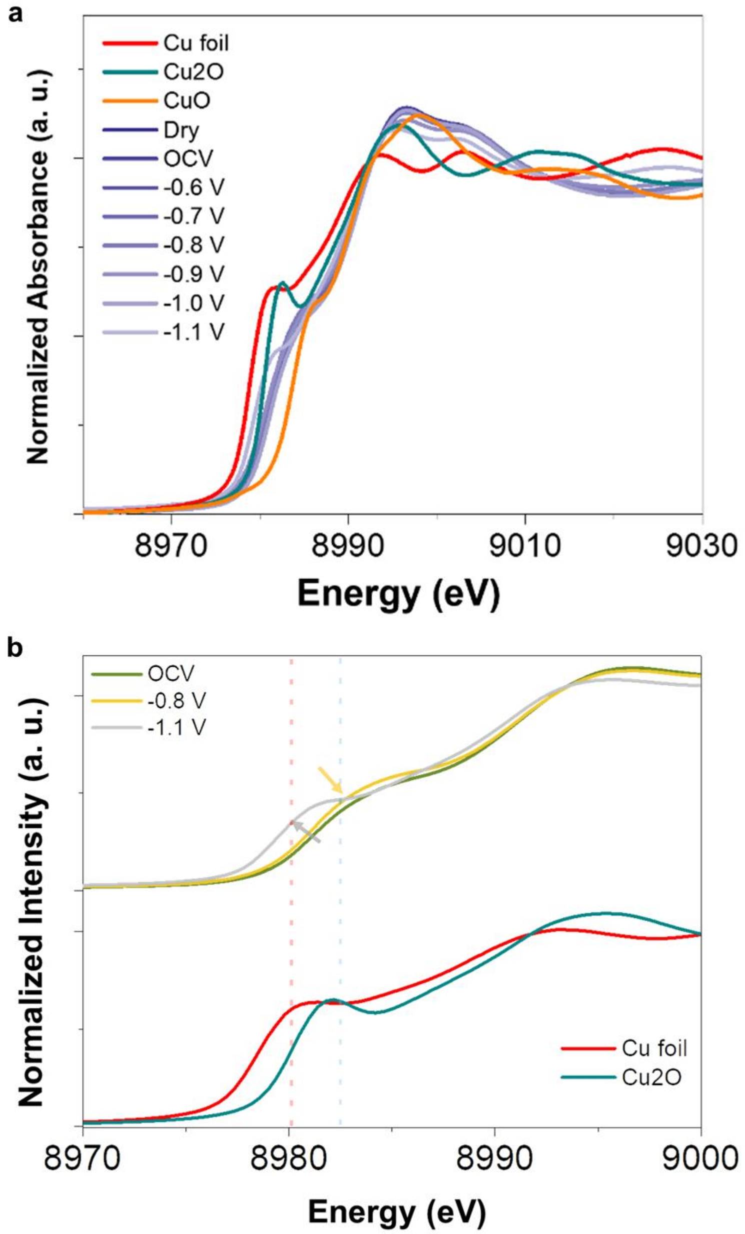

Figure S7. In situ XANES analysis of N-Cu SAC during CO_2RR. (a) In situ Cu K-edge XANES spectra of N-Cu SAC and references at various applied potentials in CO_2-saturated 0.1M $KHCO_3$ solution. (b) Magnified in situ Cu K-edge XANES spectra of N-Cu SAC at OCV, -0.8 V and -1.1 V, along with those of Cu foil and Cu_2O references.

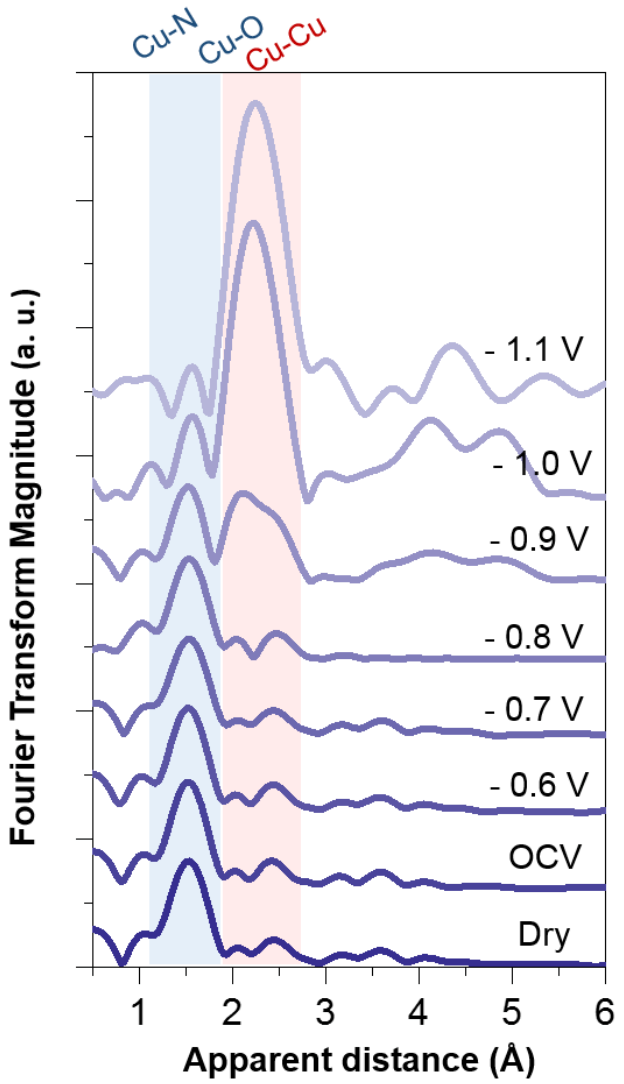

Figure S8. In situ EXAFS analysis of N-Cu SAC during CO_2RR. In situ R-space Cu K-edge EXAFS spectra of N-Cu SAC at various applied potentials in CO_2-saturated 0.1M $KHCO_3$.

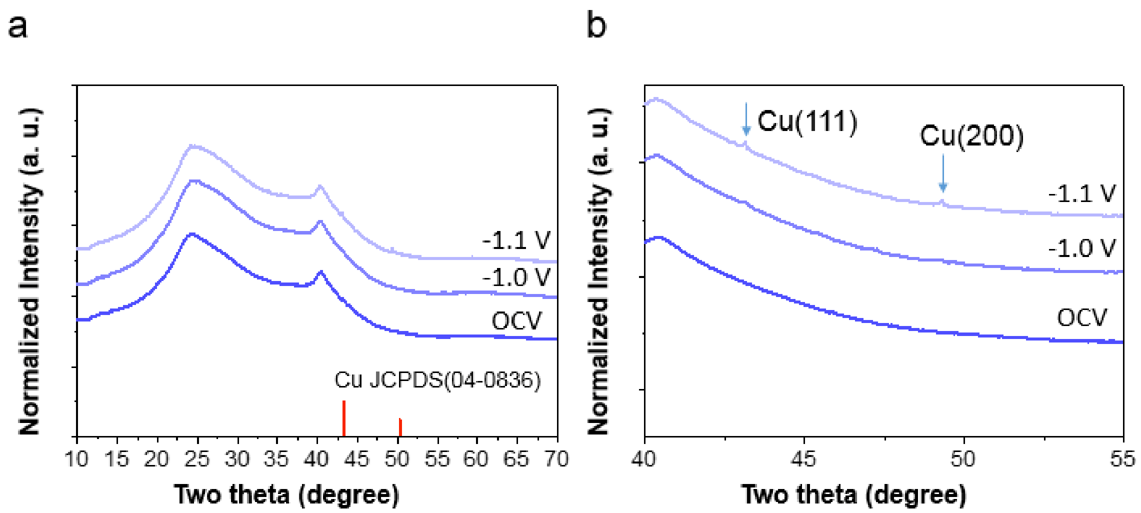

Figure S9. In situ XRD analysis of N-Cu SAC during CO_2RR. (a) In situ XRD patterns of N-Cu SAC measured at OCV, -1.0 V and -1.1 V vs RHE under the CO_2RR conditions. (b) The partially enlarged XRD patterns with 2θ between 40° and 55°.

Note: At OCV, the XRD pattern of N-Cu SAC only displays three broad peaks at 22°, 44° and 75°, which corresponds to carbon (200), (400) and (112) diffraction peaks, respectively, indicating that N-Cu SAC might remain its single atom nature without any crystalline structure formed at this potential. Once the N-Cu SAC was subjected to CO_2RR at -1.0 V vs RHE, the obtained in situ XRD patterns show two weak diffraction peaks at 43° and 50°, which can be assigned to Cu (111) and Cu (200), respectively (JCPDS 04-0836). At -1.1 V, one can observe that the intensity of those XRD peaks assigned to Cu phases become strengthened, strongly validating the structural evolution from Cu single atom to clusters in N-Cu SAC during the CO_2RR process.

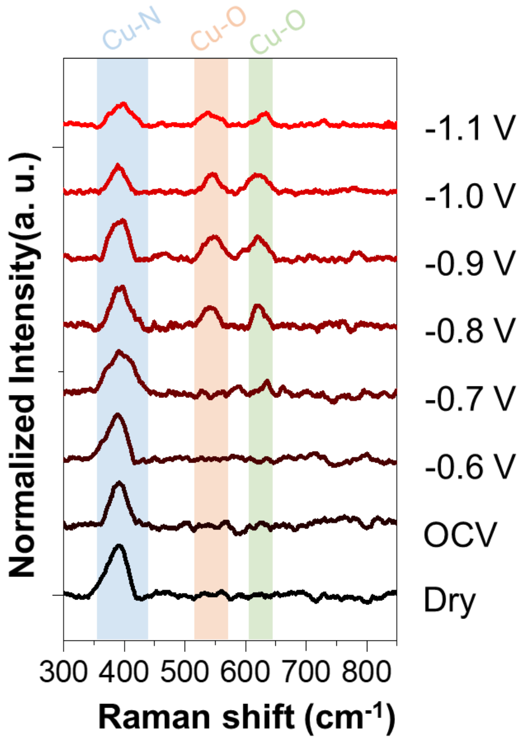

Figure S10. In situ Raman analysis of N-Cu SAC during CO_2RR. In situ Raman spectra of N-Cu SAC at various applied potentials in CO_2-saturated 0.1M $KHCO_3$ solution.

Note: In situ Raman analysis on the N-Cu SAC during CO_2RR was further performed to reveal the possible presence of Cu-O species under working conditions. The Raman bands at ~400 cm^{-1} are related to the pyrrolic nitrogen coordinated with copper single atom.[1] Upon -0.8 V versus RHE, new Raman bands at ~525 cm^{-1} and 630 cm^{-1} can be observed, which have been assigned to Cu-O bond caused by the adoption of surface hydroxyl group from the electrolyte of CO_2RR, suggesting the formation of Cu-O species on Cu sites during CO_2RR. These findings are consistent with previously reported Raman bands assigned to Cu-O bond (515~534 cm^{-1} and 620~635 cm^{-1}).[2-10] Moreover, at more negative potentials ranging from -0.7 to -1.1 V, it is noted that the intensity of Cu-N peaks gradually decreased with the most intensive Cu-O peak at -0.9 V. These results imply that the Cu-N bond breaks to generate the low coordinated N-Cu SAC, and the Cu-O bond forms before Cu cluster formation on the N-Cu SAC during CO_2RR.

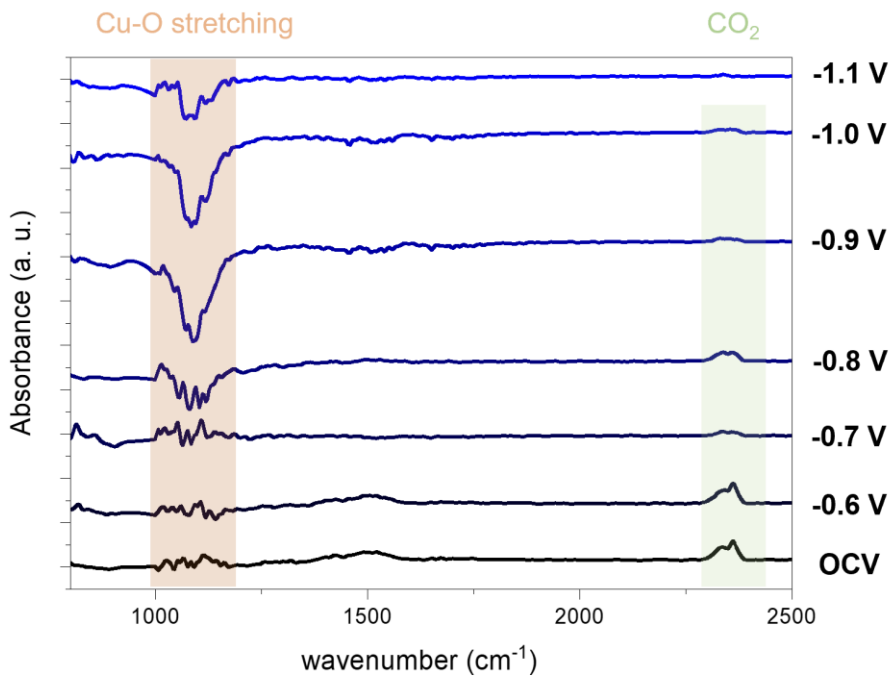

Figure S11. In situ SEIRAS analysis of N-Cu SAC during CO_2RR. In situ SEIRAS spectra of N-Cu SAC at various applied potentials in CO_2-saturated 0.1M $KHCO_3$ solution.

Note: To further reveal the possible presence of Cu-O bond under working CO_2RR conditions, we have carried out in situ SEIRAS on the N-Cu SAC during CO_2RR. The typical absorption bands observed at 2300 cm^{-1} correspond to the asymmetric stretching mode of CO_2.[11] Impressively, a new peak at 1130 cm^{-1}, assigned to the Cu-O stretching,[12] begins to appear at -0.8 V. Moreover, the 1130 cm^{-1} absorption band exhibits the highest intensity at -0.9 V and then shows a gradual intensity decrease with further increasing the cathodic potential, which is consistent with our in situ Raman results, further suggesting the presence of Cu-O bond in the N-Cu SAC during CO_2RR.

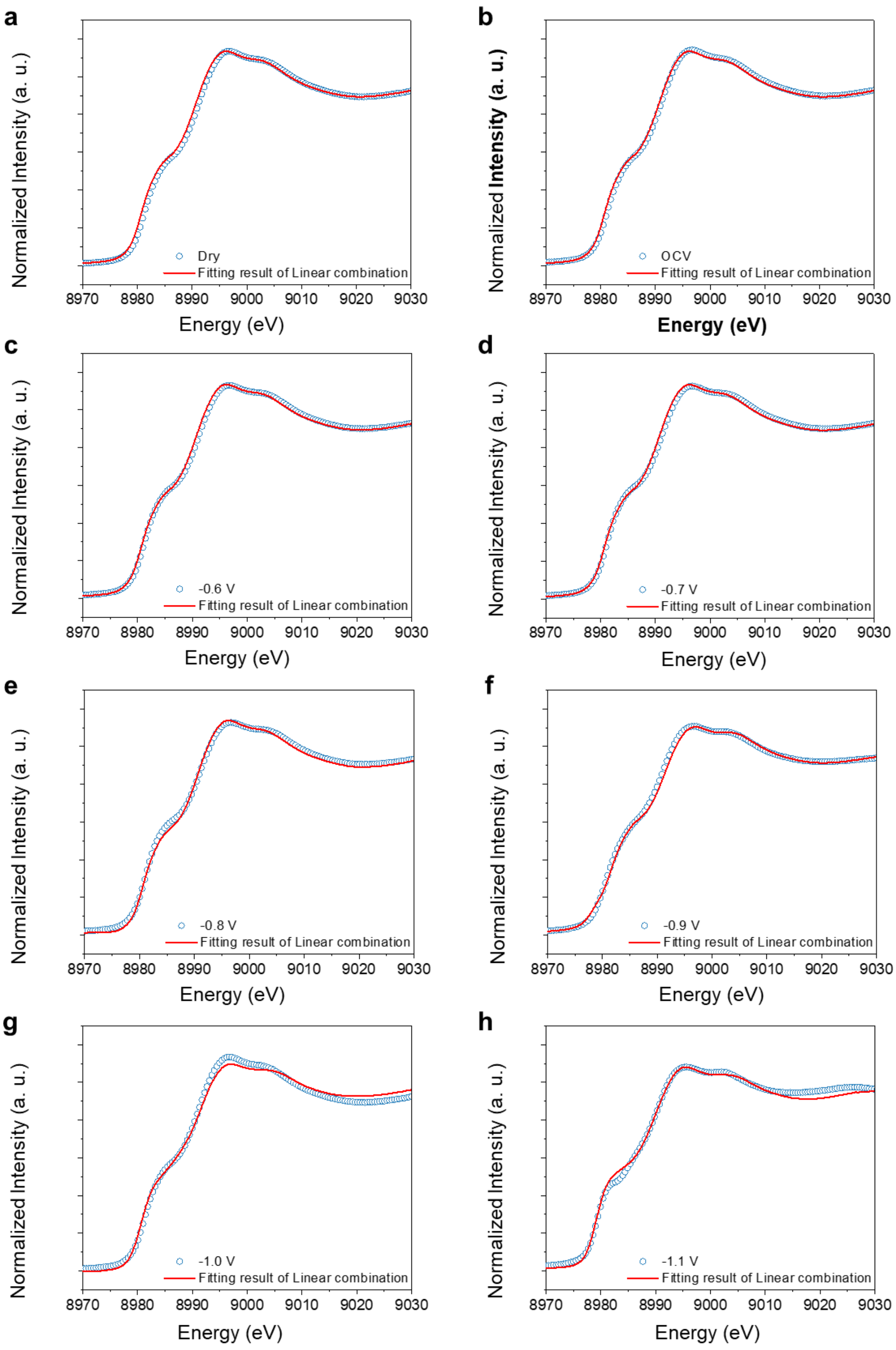

Figure S12. Linear combination analysis of in situ K-edge XANES spectra. Linear combination of in situ Cu K-edge XANES spectra of N-Cu SAC at (a) dry state, (b) OCV, (c) -0.6 V, (d) -0.7 V, (e) -0.8 V, (f) -0.9 V, (g) -1.0 V and (h) -1.1 V vs RHE.

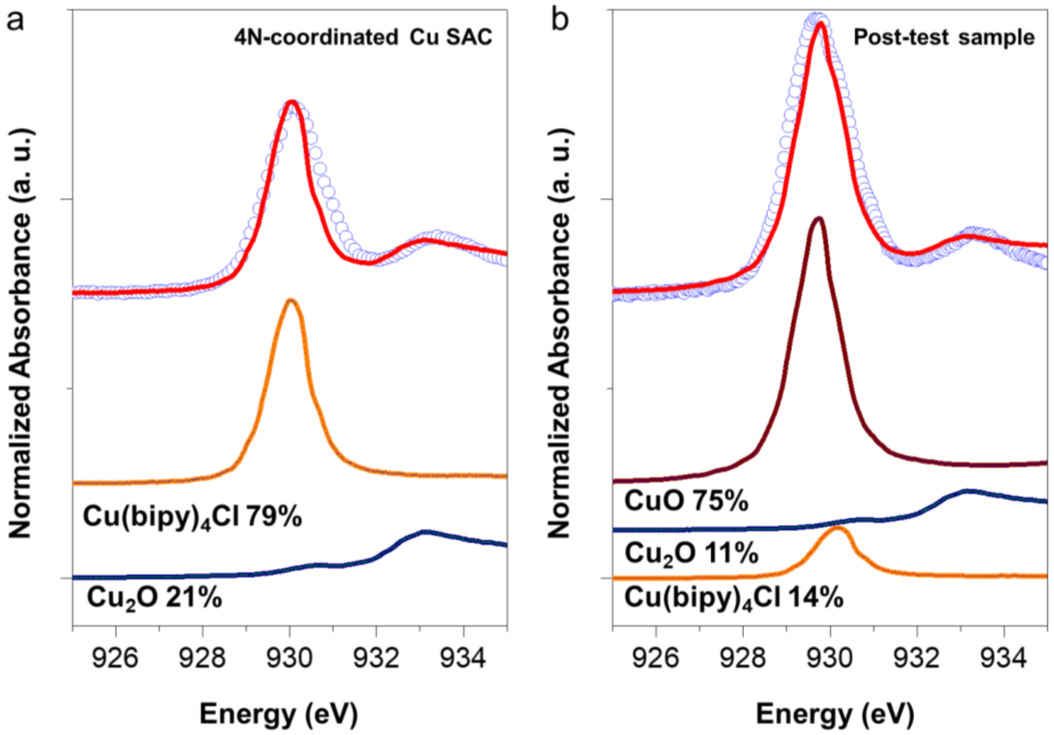

Figure S13. Linear combination analysis of L-edge XANES spectra. Linear combination fitting of Cu *L*-edge XANES spectra of (a) N-Cu SAC and (b) corresponding post-test sample (after the CO_2RR at -1.1 V vs RHE for 1 h).

Note: The linear combination analysis on Cu *L*-edge spectra shows that the as-prepared N-Cu SAC consists of 79% Cu(II)N_4 and 21% Cu(I)$_2$O, thus the initial oxidation state is determined to be 1.8. After the CO_2RR at -1.1 V vs RHE for 1 h, the composition of post-test catalyst is composed of 11% Cu(I)$_2$O, 14% Cu(II)N_4 and 75% Cu(II)O with +1.9 oxidation state. Both *K*- and *L*- edge XAS results reveal the similar oxidation state of initial N-Cu SAC. Notably, compared with in situ *K*-edge XAS results, the relatively higher oxidation state of Cu sites was observed by *L*-edge XAS spectra, which can be ascribed to the appearance of rapidly oxidized species upon releasing the applied potentials.

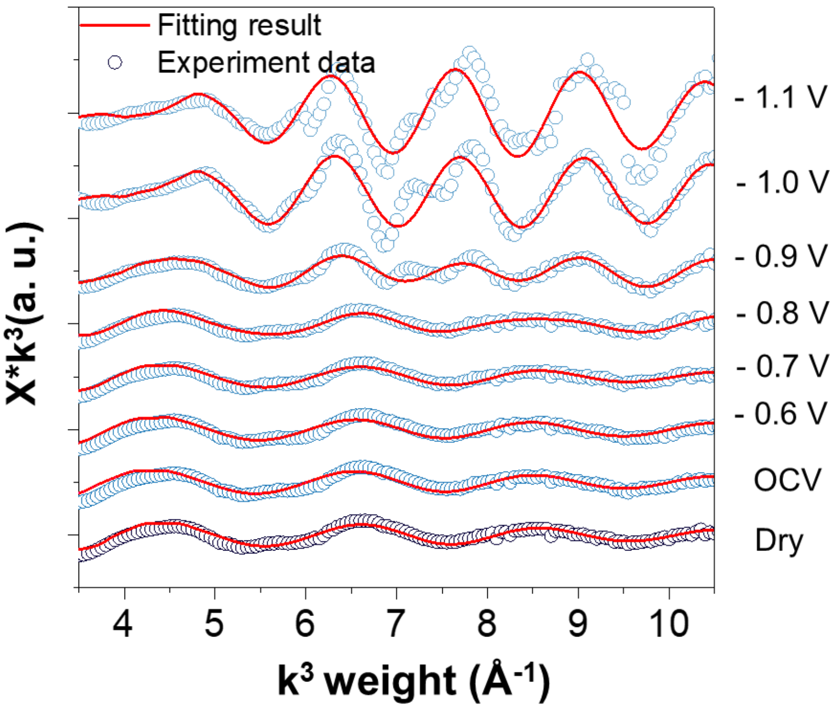

Figure S14. EXAFS fitting curves in k-space. Fitting results of in situ k-space Cu K-edge EXAFS spectra of N-Cu SAC at various applied potentials in 0.1 M CO_2-saturated $KHCO_3$. Fitting structural parameters are gathered in Table S2.

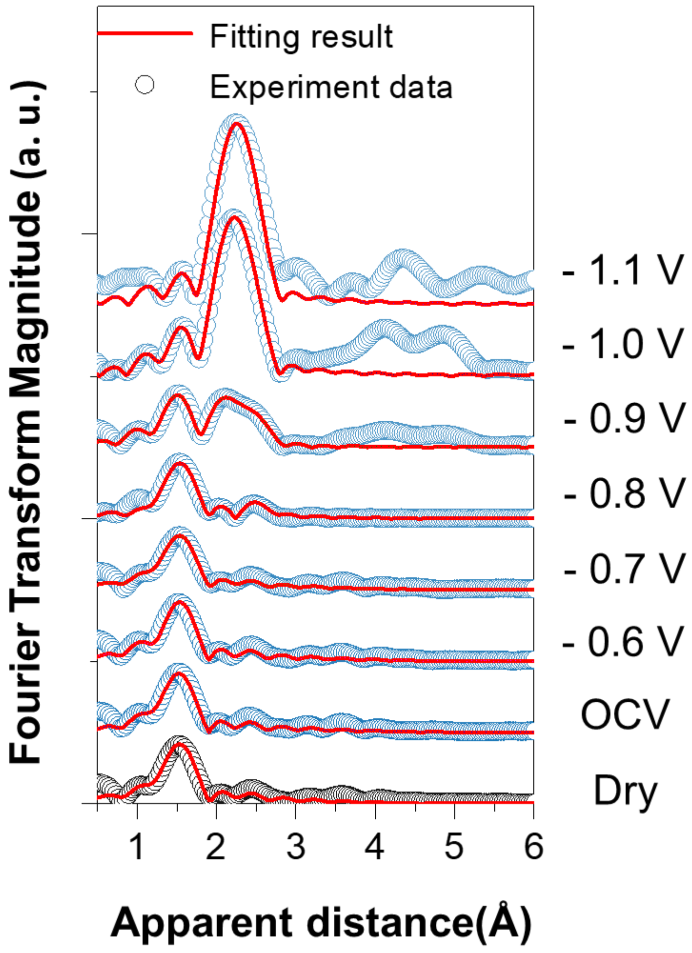

Figure S15. EXAFS fitting curves in R-space. Fitting results of in situ R-space Cu K-edge EXAFS spectra of N-Cu SAC at various applied potentials in 0.1 M CO_2-saturated $KHCO_3$. Fitting structural parameters are gathered in Table S2.

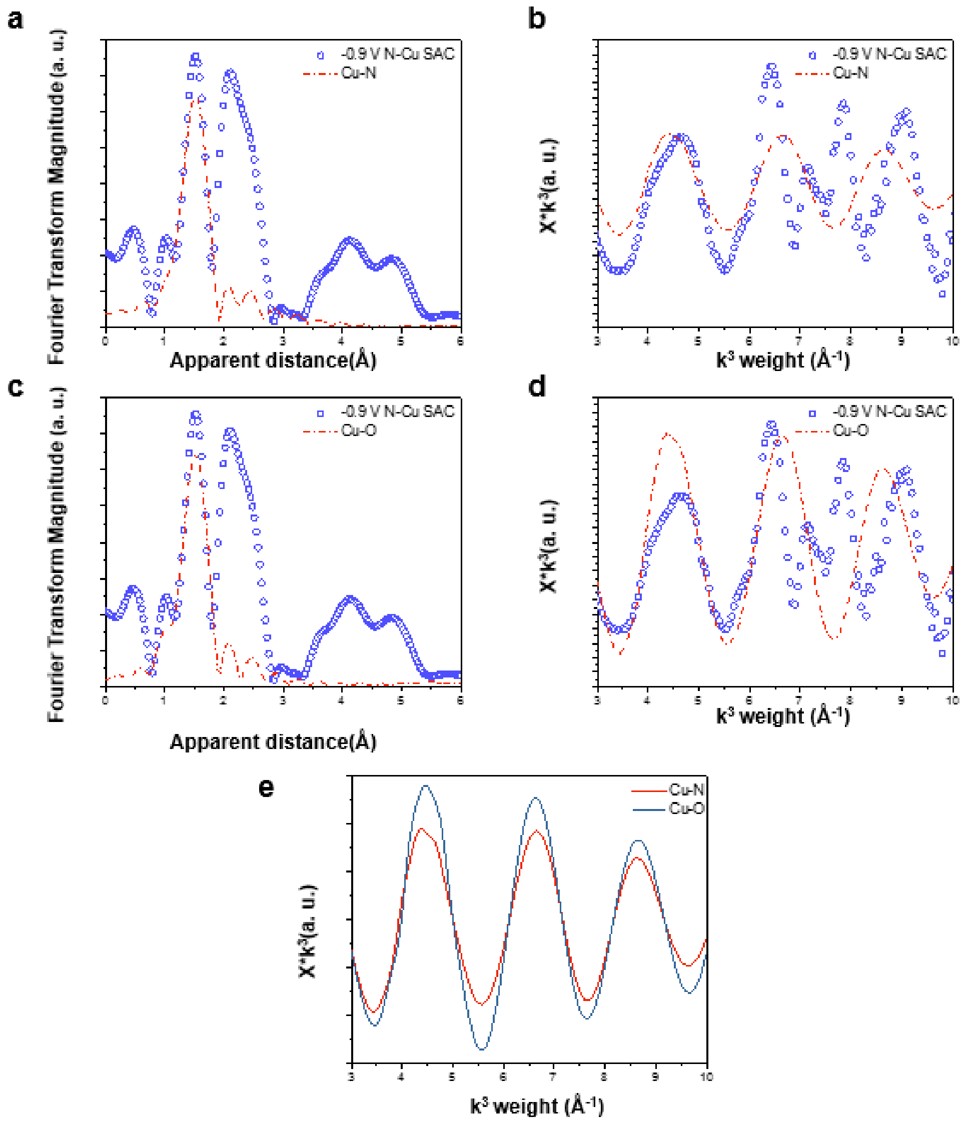

Figure 16. Comparison of EXAFS fitting results of N-Cu SAC at -0.9 V with different single path fitting. (a) EXAFS r-space spectra were Fourier-transformed in a *k*-range from 3 to 10 $Å^{-1}$ from k^3-weighted *k*-space spectra. (b) Corresponding *k*-space spectra of N-Cu SAC. Note: blue circle represents experimental data, red line reveals the corresponding fitting data with single path only, Cu-N, for the first shell. (c) EXAFS r-space spectra were Fourier-transformed in a k-range from 3 to 10 $Å^{-1}$ from k^3-weighted k-space spectra. (d) Corresponding *k*-space spectra of N-Cu SAC. Note: blue circle represents experimental data, red line reveals the corresponding fitting data with single path only, Cu-O, for the first shell. (e) Simulated *k*-space EXAFS spectra of Cu-N and Cu-O with identical coordination number (4.0) and interatomic distance (2.0).

Note: It can be clearly seen that individual Cu-O or Cu-N path cannot well fit the experimental spectra for both r- and k-space. Significantly, by comprehensive consideration of both Cu-N and Cu-O contributions,

the fitting results well match with experimental data. Thus, we can claim that the multipath fitting for the first shell is highly necessary.

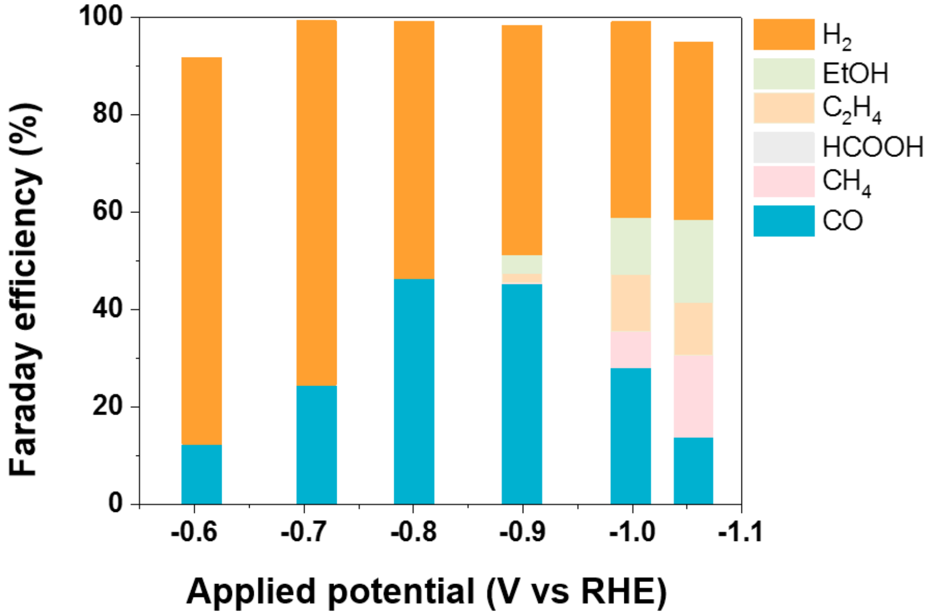

Figure S17. Electrochemical CO_2RR evaluation. Potential-dependent product profile on the N-Cu SAC for CO_2RR.

Note: The N-Cu SAC achieved a FE of almost 100% over the entire electrode potential range when all products including C1, C2 and H_2 products were considered. Furthermore, one can clearly observe that H_2 FE on the N-Cu SAC dominated above 80% at a small overpotential (-0.6 V vs RHE). As the applied potentials went more negative (-0.7 and -0.8 V vs RHE), H_2 FE was observed to decrease with the remarkably increased FE of CO products, indicating an improved selectivity towards CO_2-to-CO conversion. At the potentials more negative than -0.8 V, both H_2 and CO FE decreased with increasing the potentials, while the FE of C_2H_5OH, C_2H_4 and CH_4 products exhibited an increased trend.

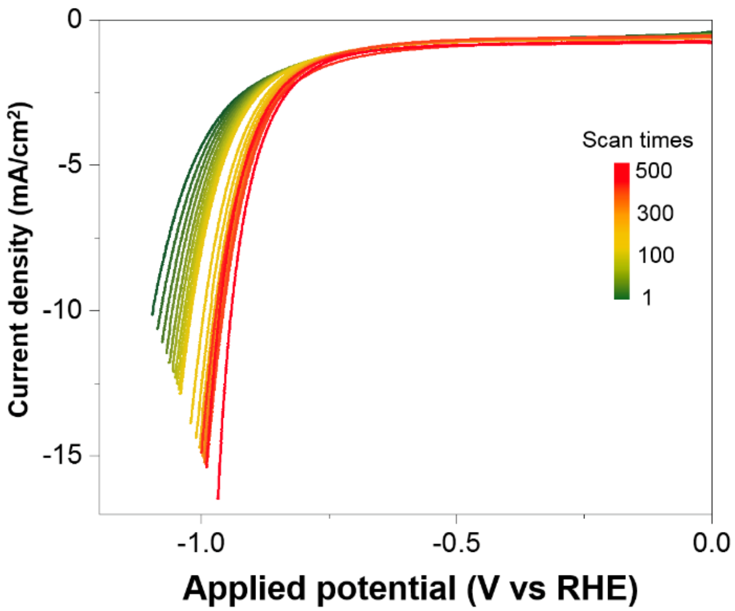

Figure S18. Repetitive liner sweeps voltammetry curves. The liner sweeps voltammetry curves of N-Cu SAC in 0.1 M CO_2-saturated $KHCO_3$ with 500 cycles.

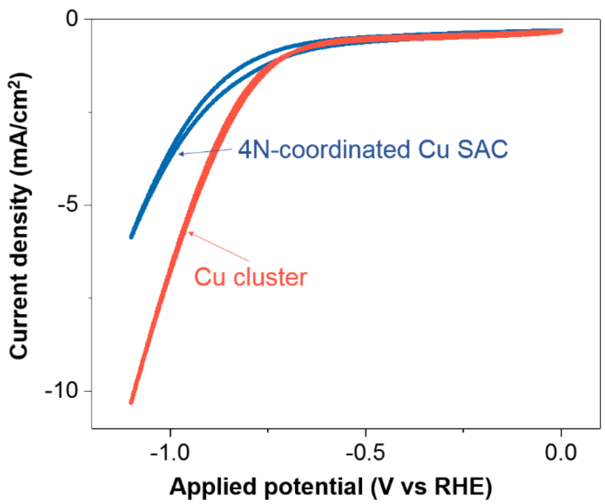

Figure S19. Cyclic voltammetry curves. Cyclic voltammetry curves of N-Cu SAC and post-reaction Cu clusters at scan rate of 100 mV/s in CO_2-saturated 0.1M $KHCO_3$ solution.

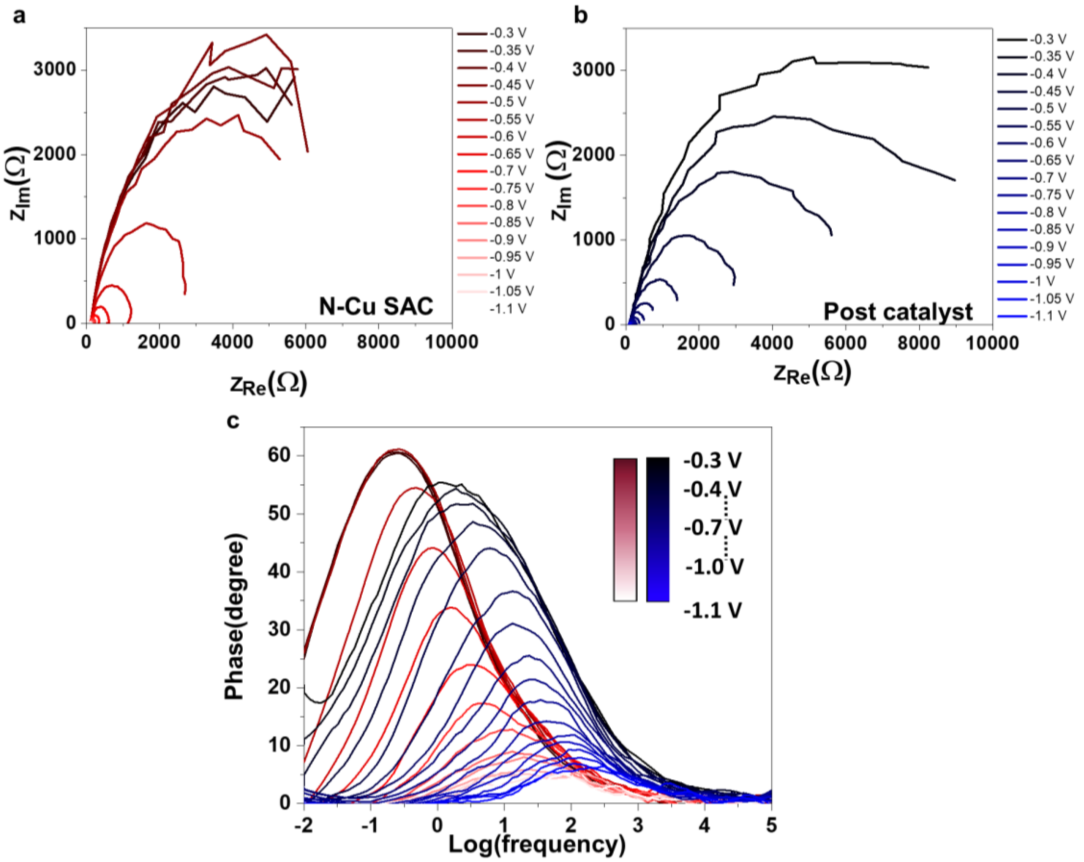

Figure S20. In situ electrochemical impedance spectroscopy (EIS) measurements. Potential-dependent Nyquist plots for N-Cu-SAC (a) and post-test catalyst (b). (c) Phase angle versus log(frequency) plots of EIS data recorded at various potentials from -0.3 to -1.1 V vs RHE for both two catalysts.

Note: Figure S20a and b exhibit the Nyquist plots of N-Cu SAC and post-test (with in situ formed Cu clusters after a chronoamperometry of -1.1 V vs RHE for 1 h) samples with an increase in applied external bias. The two Nyquist plots show the distinct semicircles at various applied potentials, which indicates that the N-Cu SAC after the CO_2RR (post-test sample) might have quite different structural features relative to the N-Cu SAC. It can be further seen from Figure S20c that, with increasing potentials from -0.3 to -1.1 V vs RHE, the phase peak in N-Cu SAC gradually shifted from 10^{-1} to 10^{2} Hz, while it moved from 10^{0} to 10^{2} Hz in the post-testing catalyst. The observed distinct frequencies further imply the different structures in these two catalysts. Notably, at -1.1 V vs RHE, it was found that the frequencies of phase peaks in these two samples were quite similar. Such results strongly validate that the N-Cu SAC underwent structural evolutions from single atom to Cu clusters under the CO_2RR conditions, which are consistent with other electrochemical and physical characterizations.

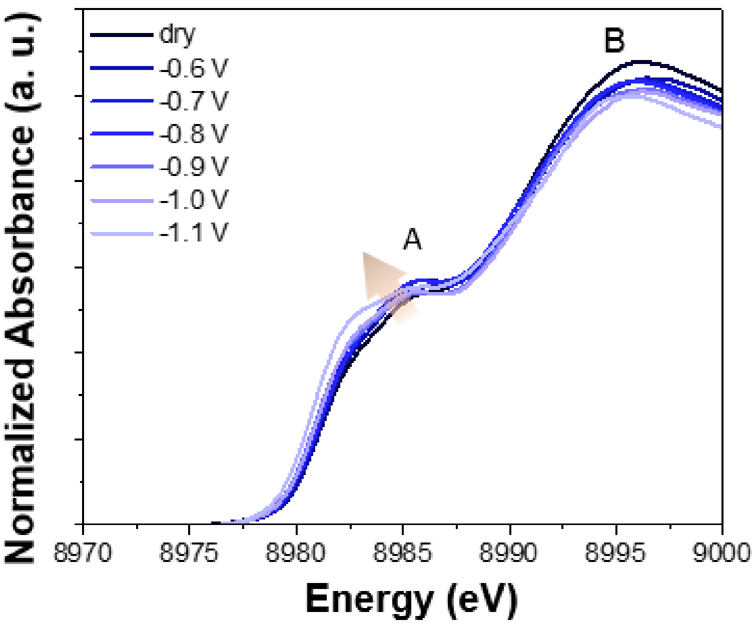

Figure S21. In situ HERFD-XAS analysis. In situ Cu K-edge high-energy resolution fluorescence detected XAS (HERFD-XAS) spectra for N-Cu SAC at various potentials in 0.1 M CO_2-saturated $KHCO_3$.

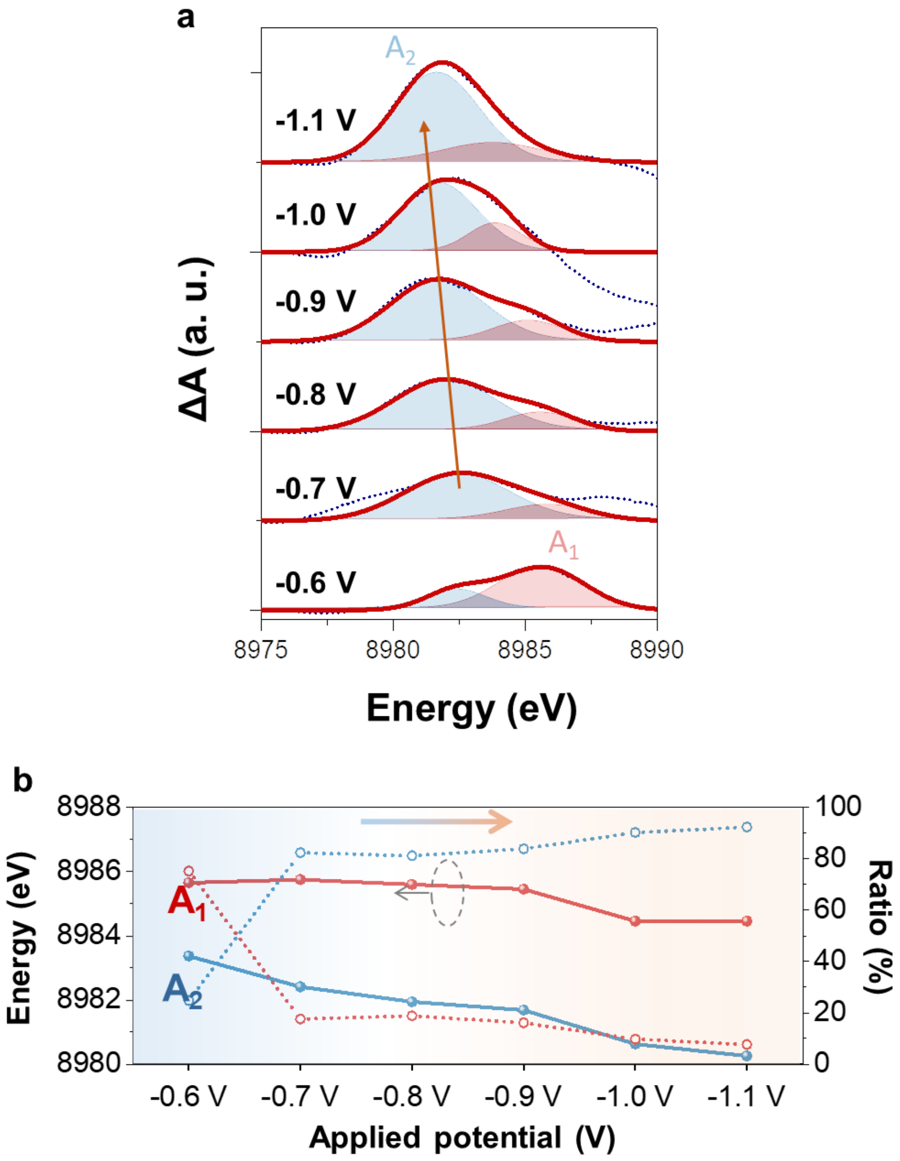

Figure S22. In situ HERFD-XANES fitting results. (a) Differential spectra of Cu K-edge high-energy resolution fluorescence detected XAS (HERFD-XAS) spectra for N-Cu SAC using the spectrum of dry sample as the reference at various applied potentials during CO_2 reduction. (b) Quantitative analysis of the differential spectra for Cu K-edge HERFD-XAS at potentials ranging from -0.6 to -1.1 V vs RHE.

Note: As compared with those at less cathodic potentials, the feature A2 at -1.0 V and -1.1 V vs RHE displays a much more remarkable increase in intensity, and moreover, it shows an obvious shift toward low energy region. Such significant changes imply the greatly strengthened interactions and covalency between neighboring Cu sites (originating from in situ formed Cu clusters) and intermediate because of

Cu-N bond breaking, which should be responsible for the favorable C-C coupling process and C2 production.

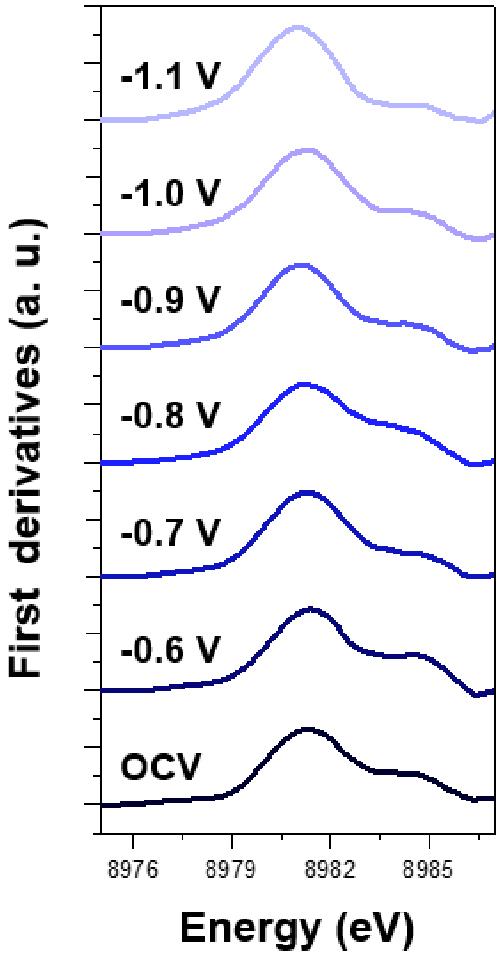

Figure S23. First derivatives of in situ HERFD-XANES spectra. First derivatives of Cu K-edge high-energy resolution fluorescence detected XAS (HERFD-XAS) spectra for N-Cu SAC at various applied potentials during CO_2 reduction.

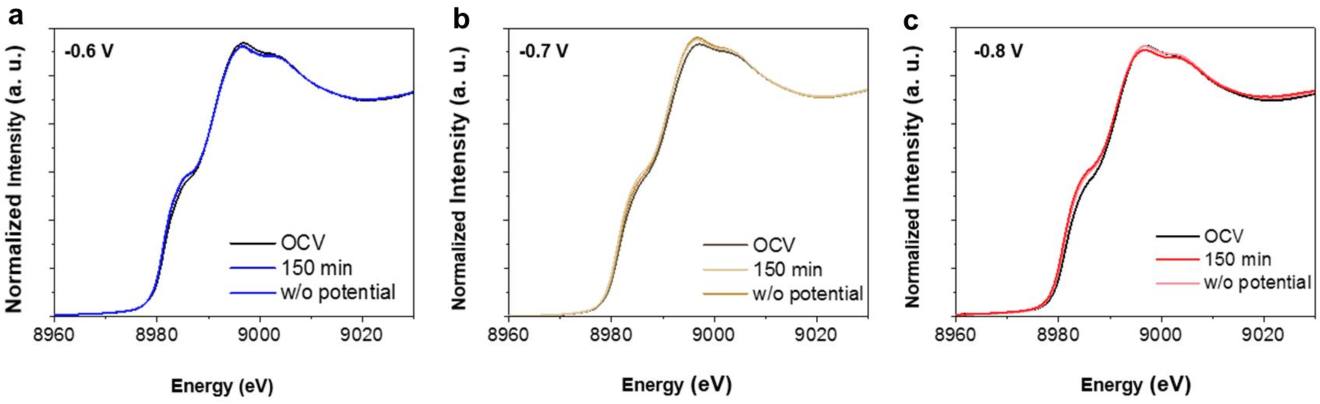

Figure 24. In situ XANES spectra under different conditions. In situ Cu K-edge XANES spectra of N-Cu SAC under -0.6 V vs RHE (a), -0.7 V vs RHE (b) and -0.8 V vs RHE (c) in 0.1 M CO_2-saturated $KHCO_3$, showing that spectroscopic features of XANES under cases of -0.6 V vs RHE and -0.7 V vs RHE were almost identical throughout 150 minutes' electrolysis, whereas a slight shift in absorption edge was evidently observed at the case of -0.8 V vs RHE.

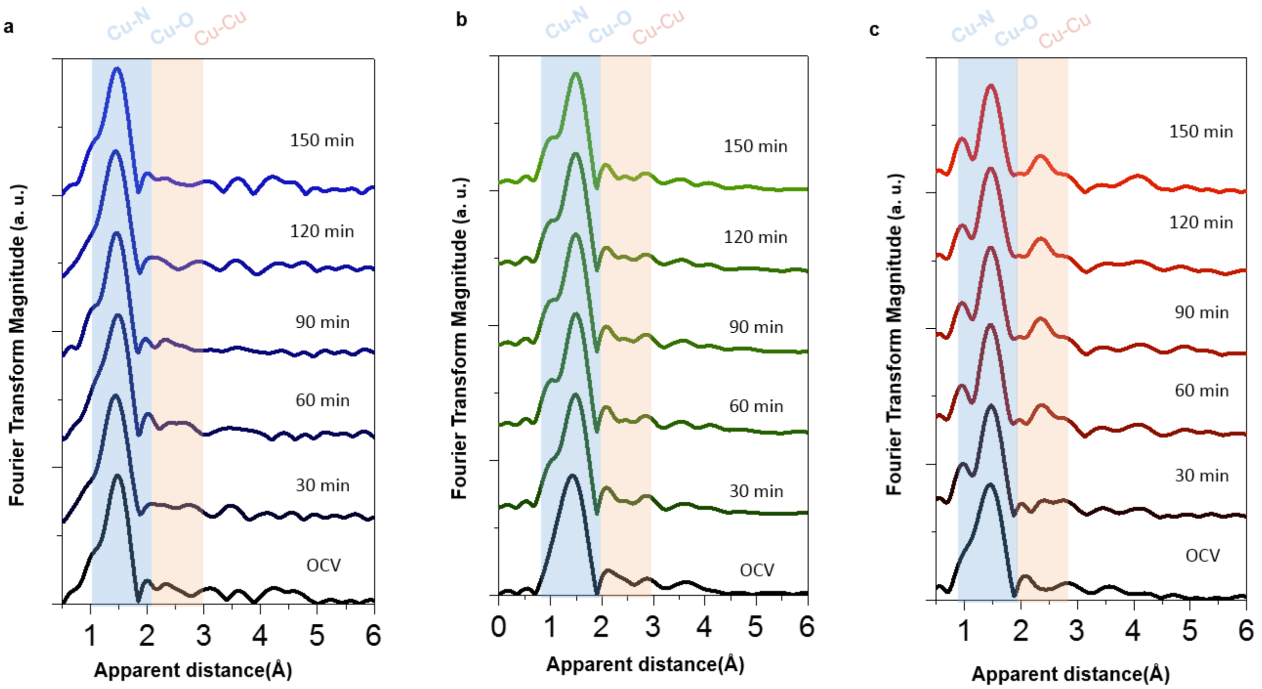

Figure 25. In situ R-space EXAFS spectra under different conditions. In situ R-space Cu K-edge EXAFS spectra of N-Cu SAC under -0.6 V vs RHE (a) -0.7 V vs RHE (b) and -0.8 V vs RHE (c) in 0.1 M CO_2-saturated $KHCO_3$, showing that the first-shell intensity shrinks (i.e., Cu-N) at -0.7 V vs RHE cases, whereas the emergence of the scattering shell related to Cu-Cu path was only found at -0.8 V vs RHE condition.

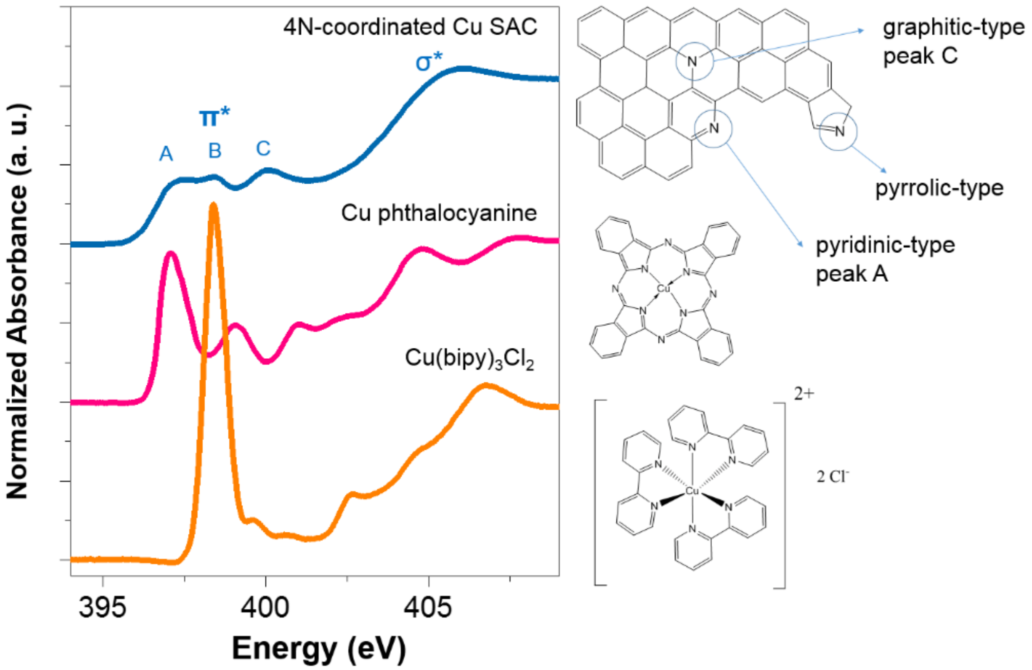

Figure S26. N K-edge XANES spectra. Cu phthalocyanine and Cu (bipy)$_3$Cl$_2$ which used as references were also showed. The scheme illustrates the nitrogen types in Cu SAC and references, showing that three peaks are found in the π^* region in which the peak A (~397.4 eV) and C (~399.8 eV) can be assigned to pyridinic-type and graphitic-type, respectively.[13]

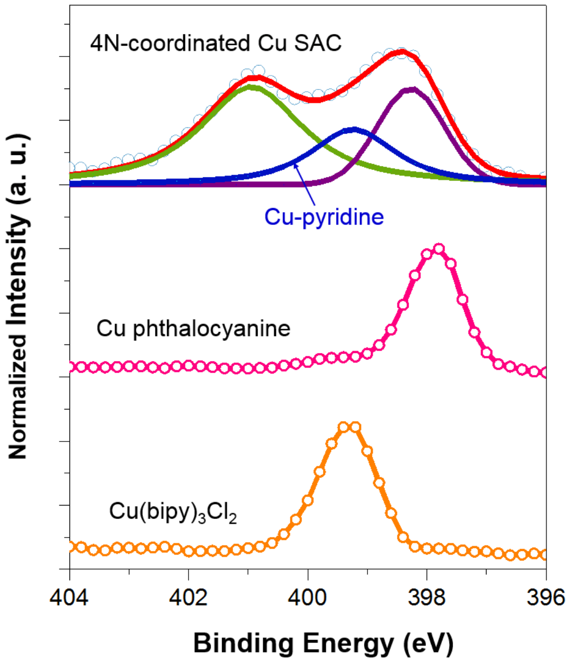

Figure S27. N 1s XPS spectra. N 1s XPS spectra of N-Cu SAC and relevant references, showing that the peaks located at 398.4 eV and 401.1 eV are assigned to the pyridinic-type and graphitic-type of nitrogen in graphene structure, respectively.[14-15]

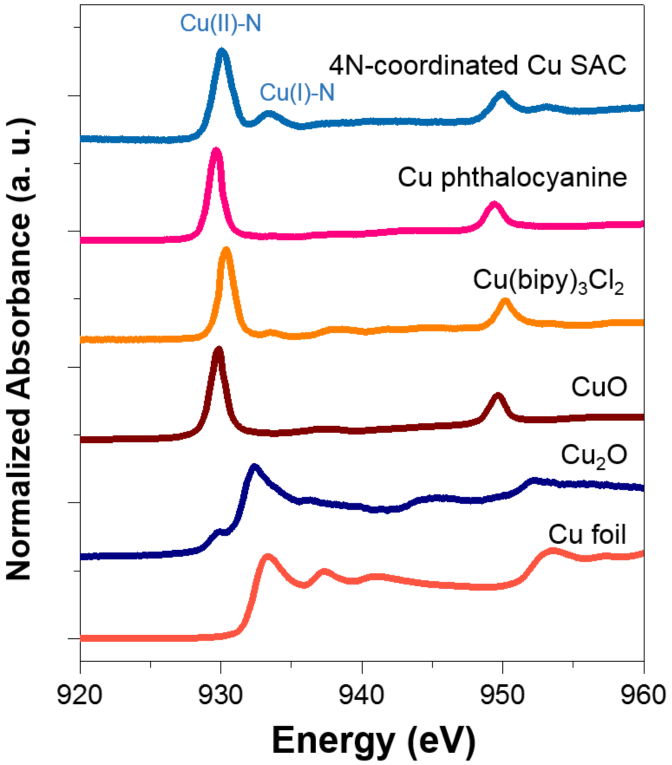

Figure S28. Cu *L*-edge XANES spectra of N-Cu SAC and references. Two intensive peaks of 932.5 eV and 935.0 eV are discerned in Cu *L*-edge XANES spectra and can be assigned to the Cu (II) and Cu(I) state with coordinated N, respectively.[16] The former one is situated between CuO and Cu_2O references and in line with that of the $Cu(bipy)_2Cl_2$, indicating that Cu(II) site is coordinated by pyridinic-type N with the presence of minor amount of N-coordinated Cu(I) state.

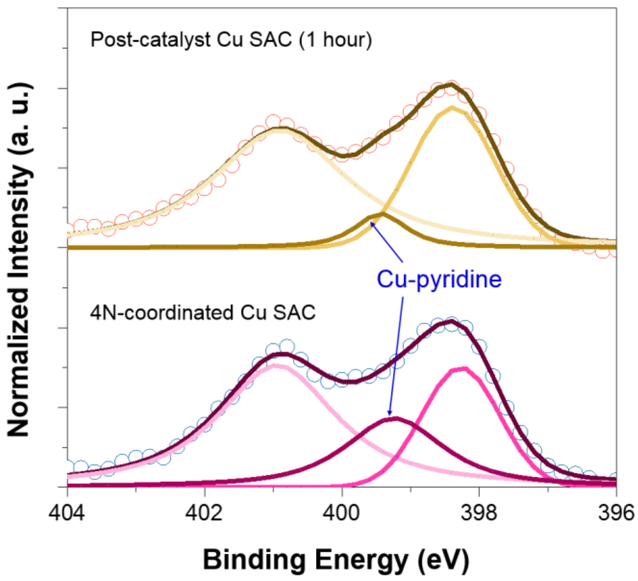

Figure S29. Comparisons between N 1s XPS spectra. N 1s XPS spectra of as-prepared N-Cu SAC and corresponding post-reaction Cu SAC after electrocatalysis at -1.1 V for one hour.

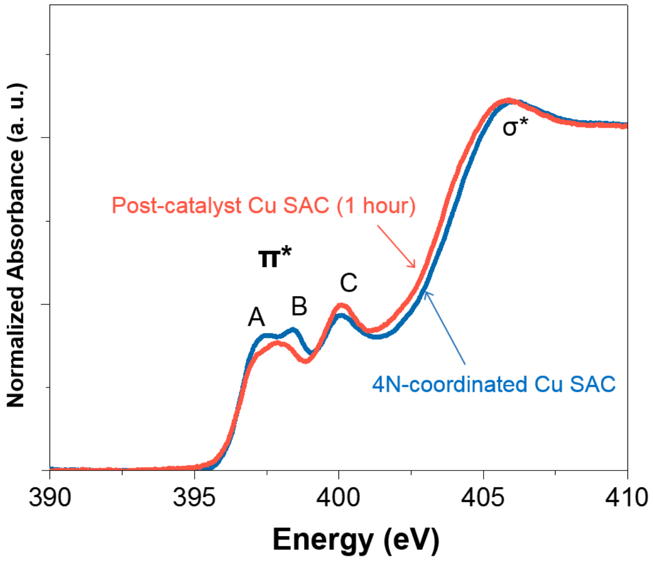

Figure S30. Comparisons between N K-edge XANES spectra. N K-edge XANES spectra of as-prepared N-Cu SAC and corresponding post-reaction Cu SAC after electrocatalysis at -1.1 V for one hour.

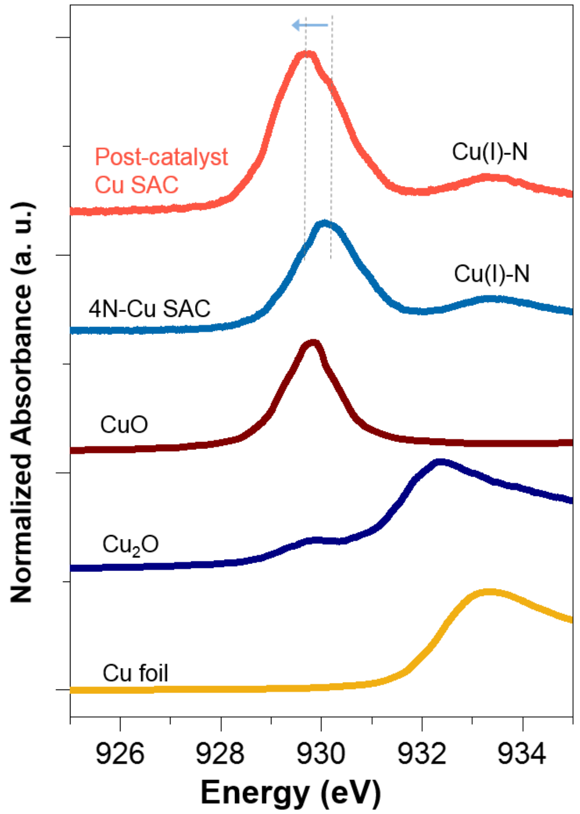

Figure S31. Comparisons between Cu L-edge XANES spectra. Cu *L*-edge XANES spectra of N-Cu SAC, relative references and corresponding post-reaction Cu SAC after electrocatalysis at -1.1 V for one hour.

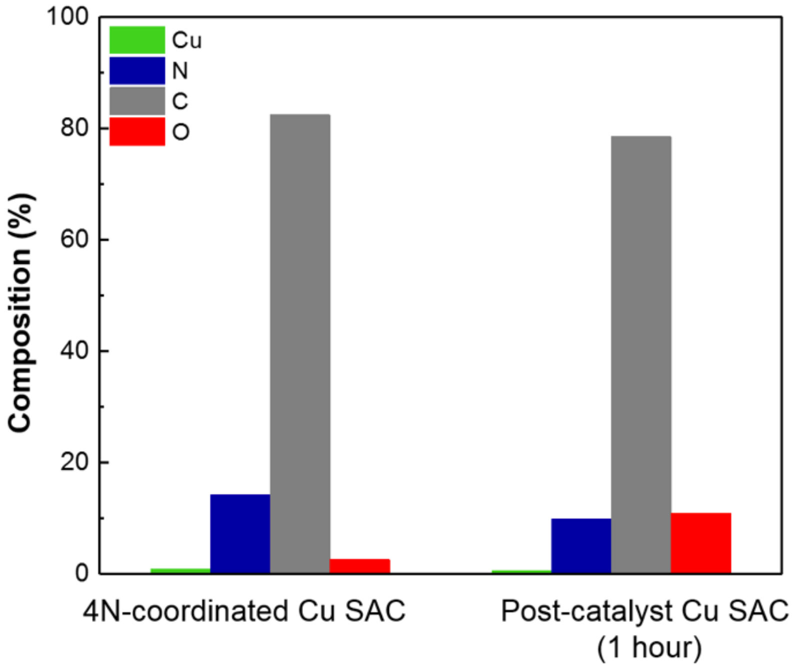

Figure S32. Compositional comparisons based on XPS analysis. Elemental analysis based on XPS for as-prepared N-Cu SAC and corresponding post-reaction Cu SAC after electrocatalysis at -1.1 V for one hour.

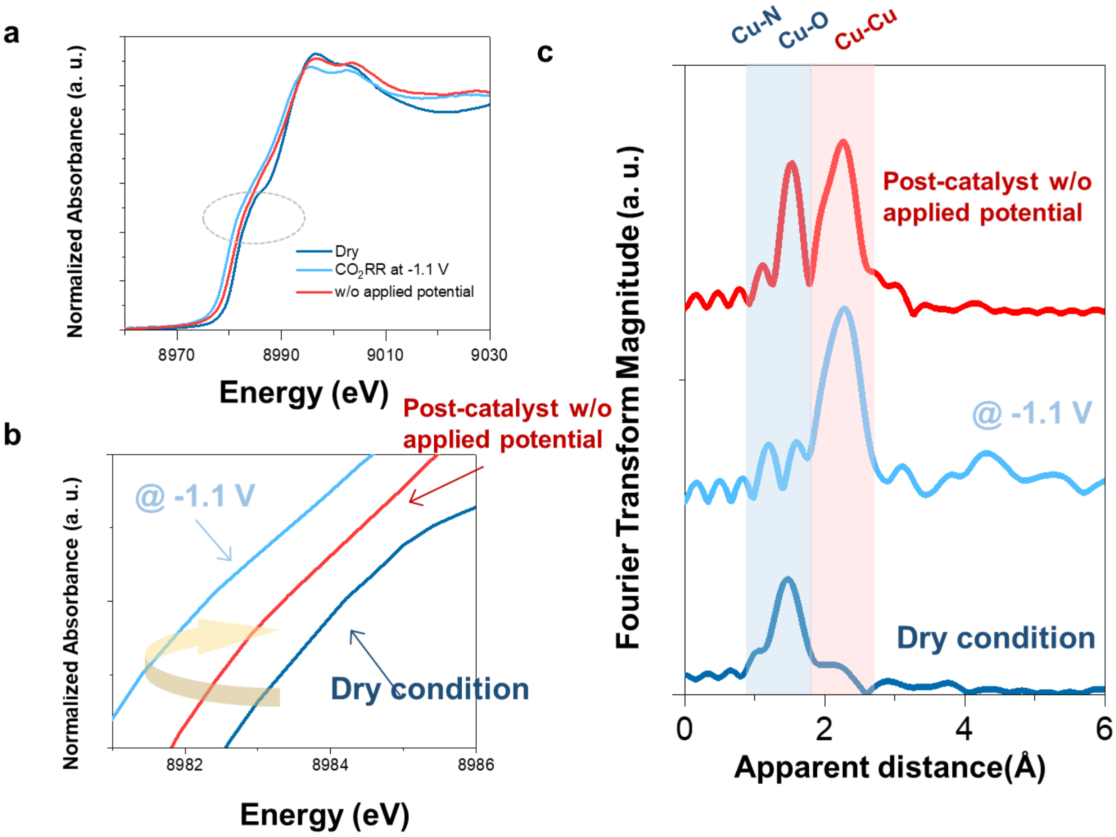

Figure S33. Dynamic atomic coordination environment of N-Cu SAC. (a) In situ Cu K-edge XANES spectra of dry N-Cu SAC (blue), catalyst at -1.1 V vs RHE (light blue), and post-test catalyst without applying potentials (red) in CO_2-saturated 0.1M $KHCO_3$ solution. (b)The 0.5 height of XANES spectra in (a). (c) Corresponding R-space Cu K-edge EXAFS spectra.

Note: We performed in situ analysis of Cu *K*-edge XAS on N-Cu SAC at -1.1 V versus RHE during CO_2RR. Furthermore, the XAS spectrum was continuously collected in the electrochemical cell without applying potentials after the same catalyst remained immersed in the electrolyte for 30 min (post-test catalyst). As shown in XANES spectra, it can be seen an edge shift to higher energies on the post-test catalyst with respect to that of the same catalyst catalyzed at -1.1 V, which indicates that the oxidation state of Cu sites partially reverses after electrocatalysis. In EXAFS spectrum of the post-test catalyst, note that the Cu-Cu scattering still remains, suggesting that in situ formed Cu clusters on the N-Cu-SAC during CO_2RR could not reversibly transform back to N-coordinated Cu SAC after electrocatalysis. Intriguingly, a remarkable peak assigned to Cu-N/O scattering at the first coordination shell was observed. Such phenomenon can be ascribed to a fact that the breaking of Cu-N bond was partially reversible when the

applied potential was released, and the low N-coordinated Cu atoms could easily adsorb the hydroxide after reactions, which contributed to the Cu-O bonding.

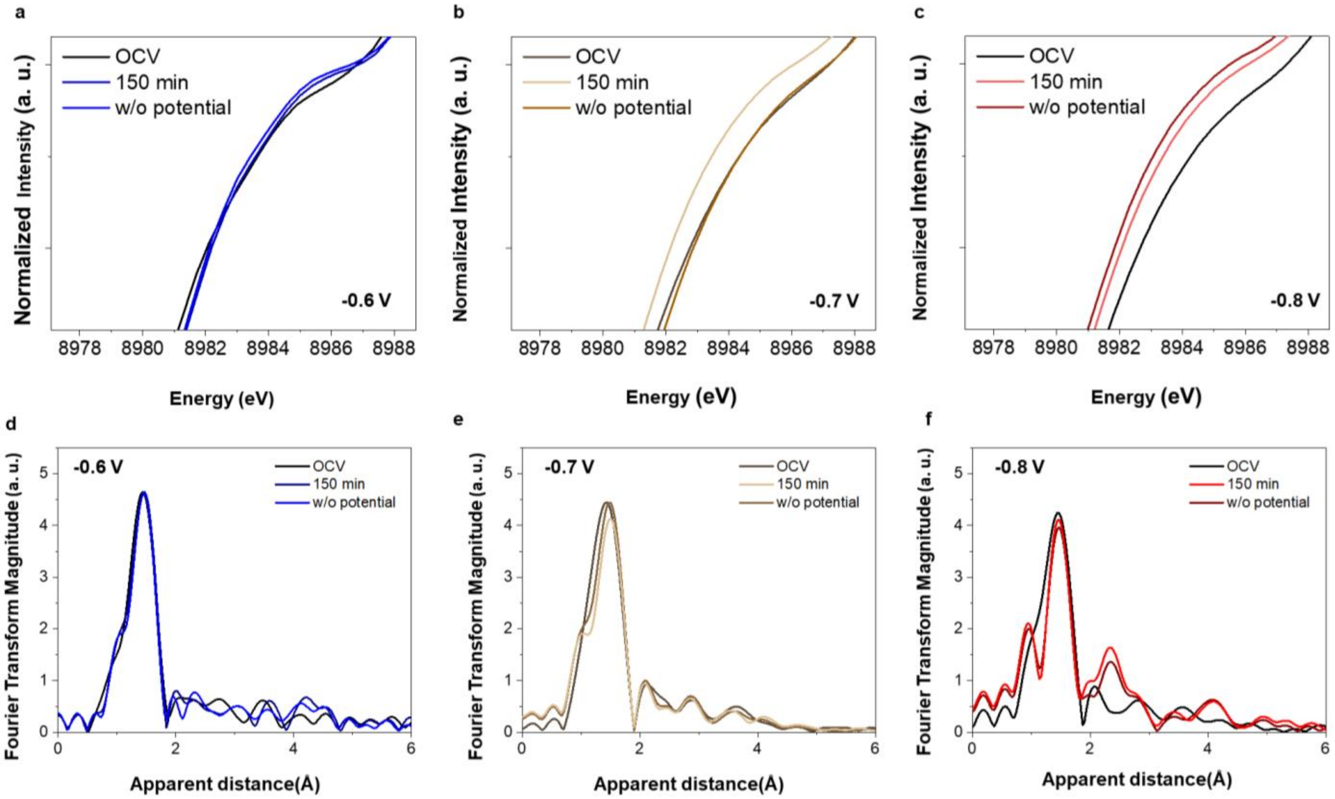

Figure S34. In situ XANES and EXAFS spectra under different conditions. In situ Cu *K*-edge XANES spectra of N-Cu SAC measured at OCV, after 150 min CO_2RR and without applied potentials for the electrocatalysis at (a) -0.6 V, (b) -0.7 V and (c) -0.8V vs RHE. Corresponding Cu *K*-edge EXAFS spectra of N-Cu SAC for the electrocatalysis at (d) -0.6 V, (e) -0.7 V and (f) -0.8V vs RHE.

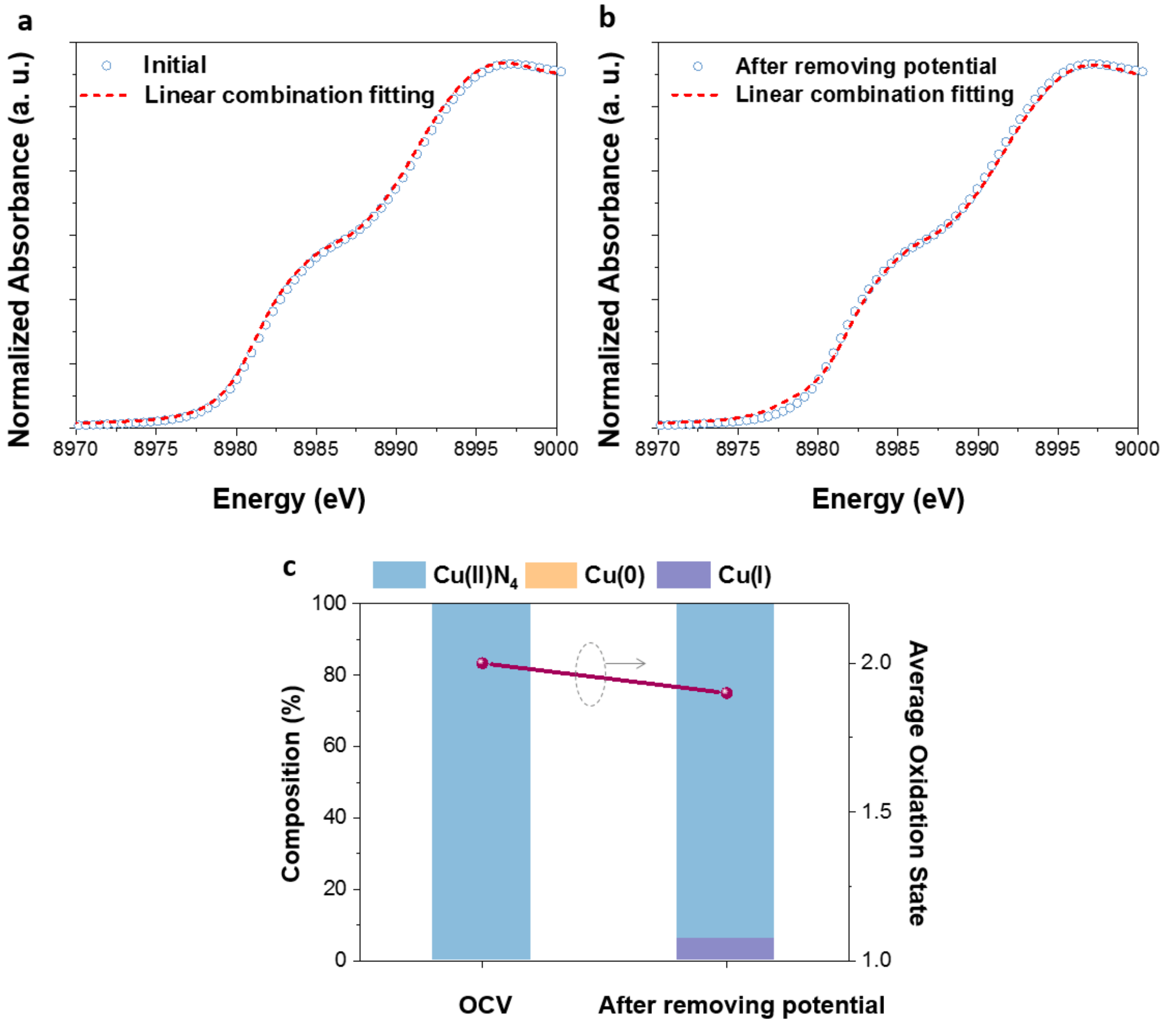

Figure S35. Linear combination analysis of XANES spectra. Linear combination result of Cu *K*-edge XANES spectra of Cu SAC measured (a) at OCV and (b) without potentials after the electrocatalysis at -0.7 V vs RHE. (c) Extracted composition and oxidation state from the linear combination analysis of *K*-edge XANES spectra.

Note: Figure S35 presented the linear combination fitting results of XANES spectra for initial and post-test N-Cu SAC samples. It revealed that the initial N-Cu SAC consisted of almost ~100% Cu(II)N_4, resulting in an average oxidation state of +2.0. After releasing the potential, the post-test sample was composed of 92% Cu(II)N_4 and 8% $Cu(I)_2O$ with the high oxidation state of about 1.9.

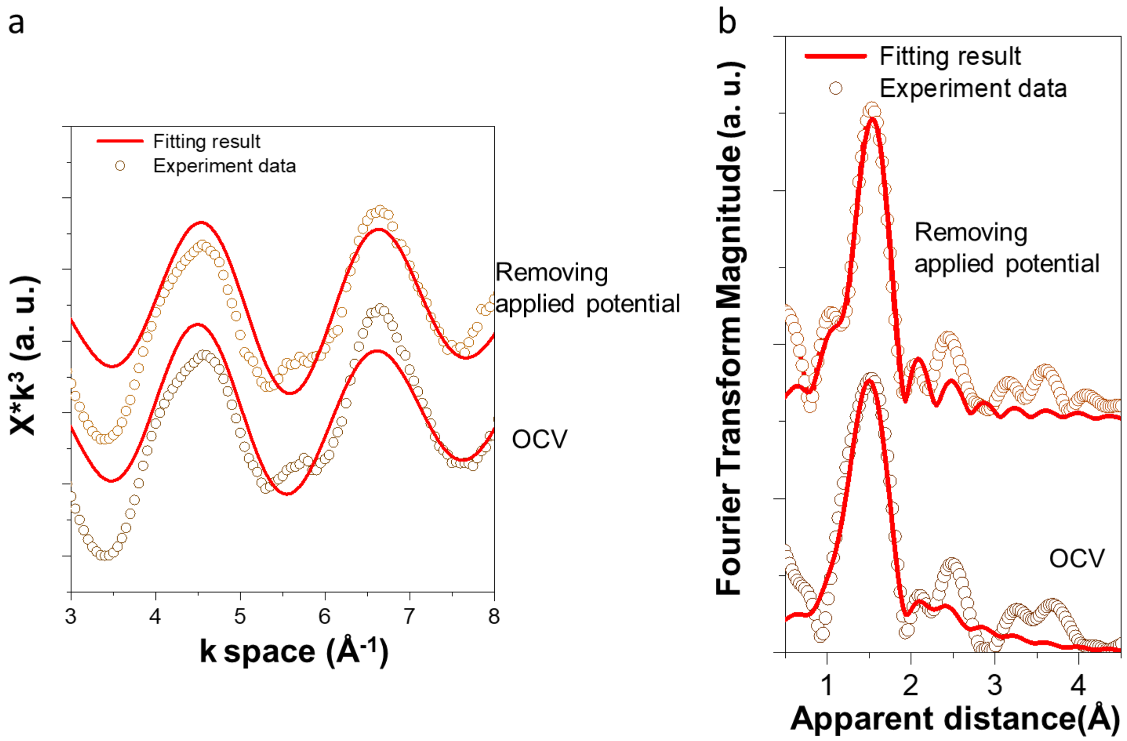

Figure S36. EXAFS fitting in k-space and R-space. Fitting results of (a) *k*-space and (b) R-space of Cu *K*-edge EXAFS spectra of N-Cu SAC measured at OCV and without potentials for the electrocatalysis at -0.7 V vs RHE.

Note: Figure S36 exhibited more informative features of the dynamic atomic configuration changes of N-Cu SAC after the CO_2RR. Specifically, for initial sample, the Cu-N in the first-shell scattering with coordination number of 3.8 was observed, indicating the 4N-coordinated configuration of N-Cu SAC. After the reactions, the fitting curves indicated that the Cu atom was coordinated by ~3 N atoms and ~1 O atom (Table S6). Based on these observations, we carefully proposed that the dynamic breaking of Cu-N bond was partially reversible when the applied potential was released, and the low N-coordinated Cu atoms could easily adsorb the hydroxide after reactions, which contributed to the Cu-O bonding.

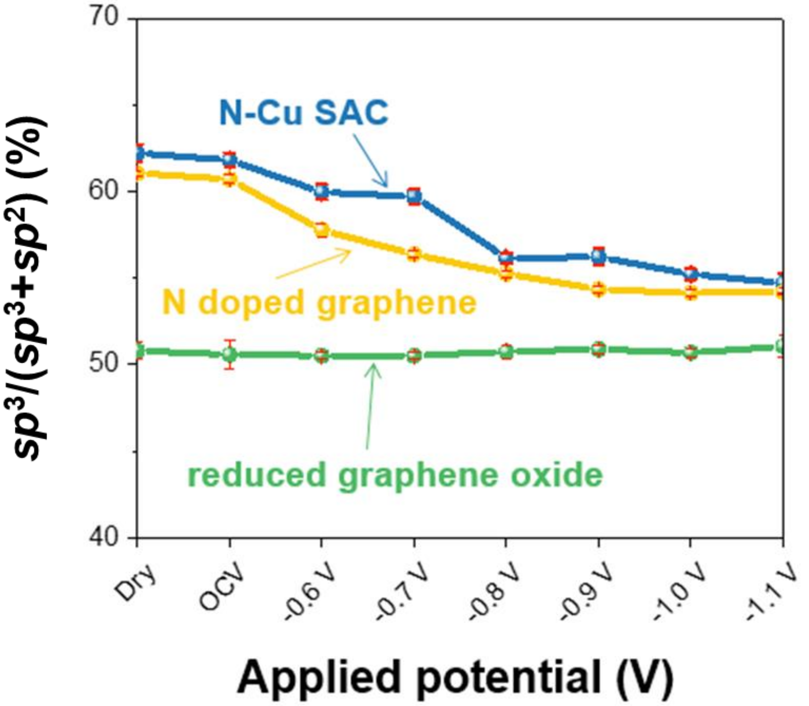

Figure S37. Analysis on *sp*^{3} contribution based on in situ Raman. Ratio of *sp*^{3} contribution in carbon for N-Cu SAC, N doped g raphene and reduced graphene oxide at various applied potentials during CO_2 reduction.

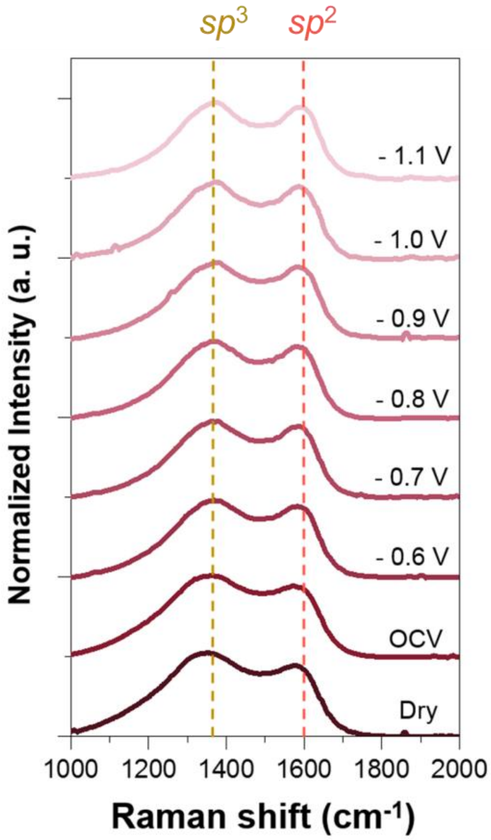

Figure S38. In situ Raman spectra. In situ Raman spectra of Cu SAC at various applied potentials in CO_2-saturated 0.1M $KHCO_3$ solution.

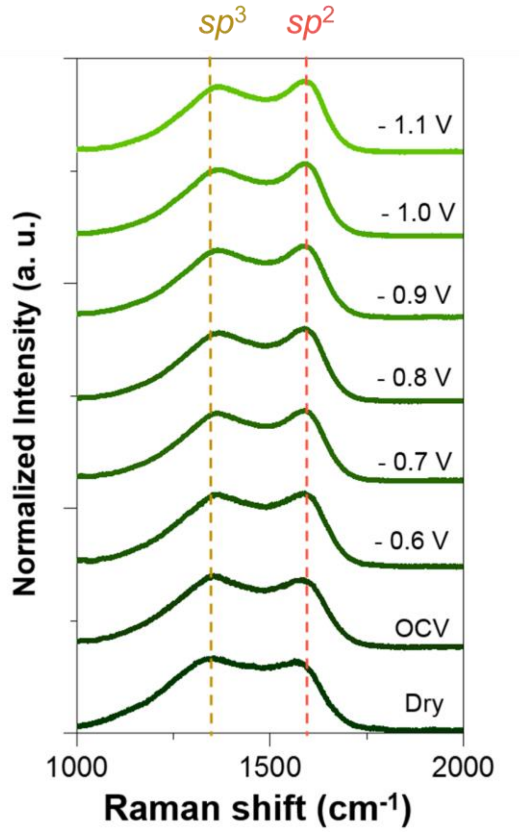

Figure S39. In situ Raman spectra. In situ Raman spectra of nitrogen-doped graphene at various applied potentials in CO_2-saturated 0.1M $KHCO_3$ solution.

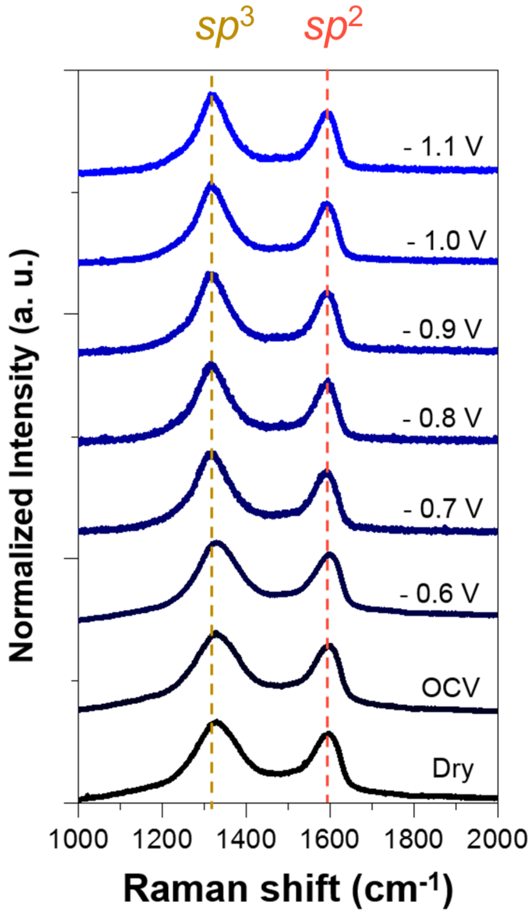

Figure S40. **In situ Raman spectra.** In situ Raman spectra of reduced graphene oxide at various applied potentials in CO_2-saturated 0.1M $KHCO_3$ solution.

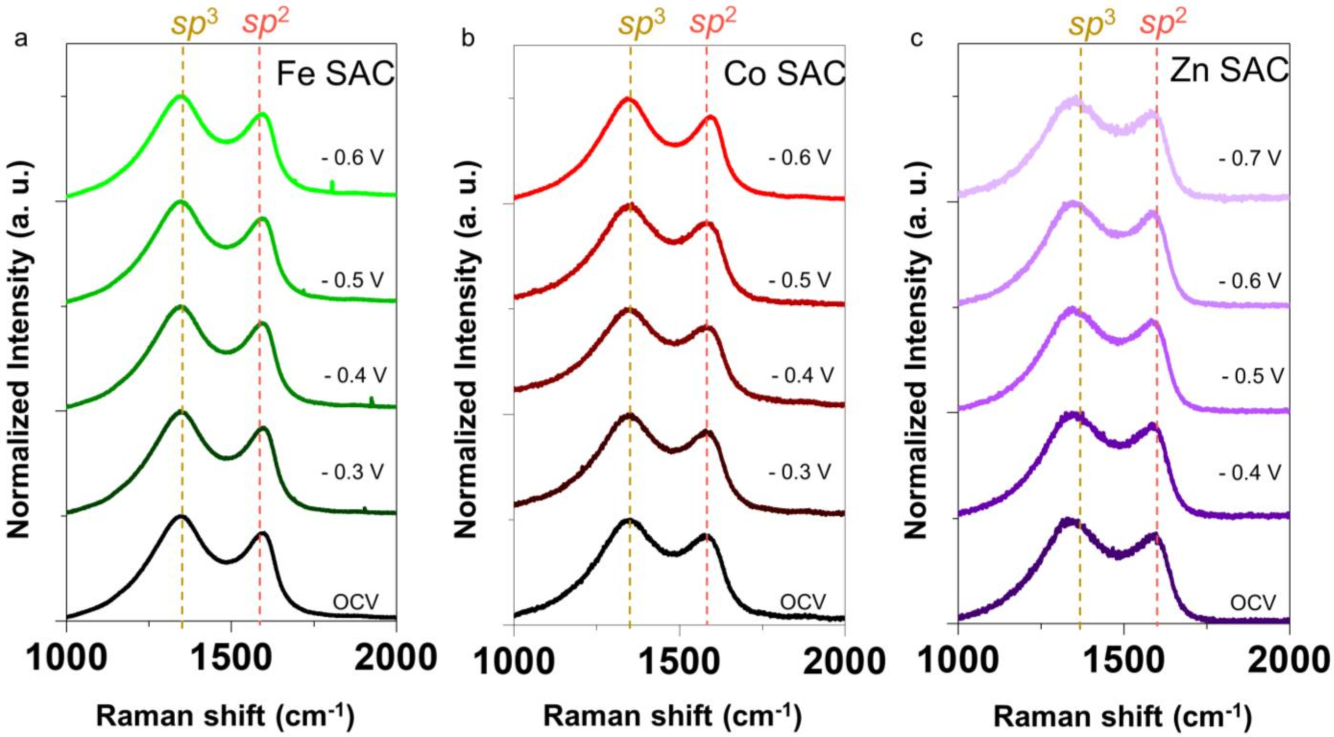

Figure S41. In situ Raman spectra. In situ Raman spectra of (a) Fe SAC, (b) Co SAC and (c) Zn SAC at various applied potentials under working CO_2RR conditions.

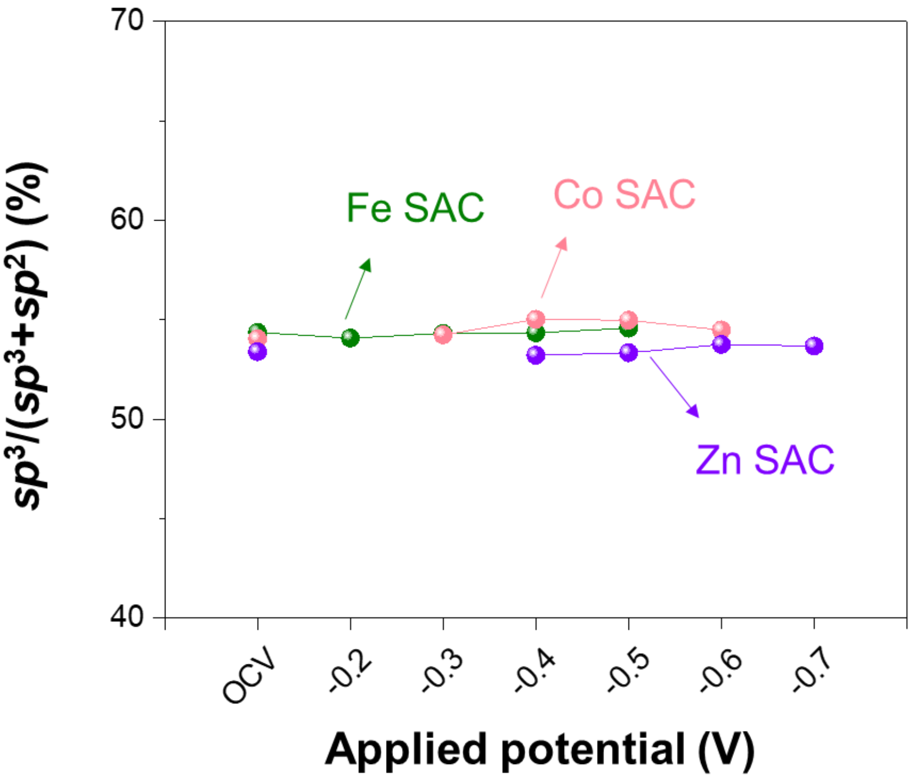

Figure S42. Analysis on *sp*^{3} contribution based on in situ Raman. Ratio of *sp*^{3} contribution in carbon for Fe, Co and Zn SACs at various applied potentials during CO_2 reduction reaction.

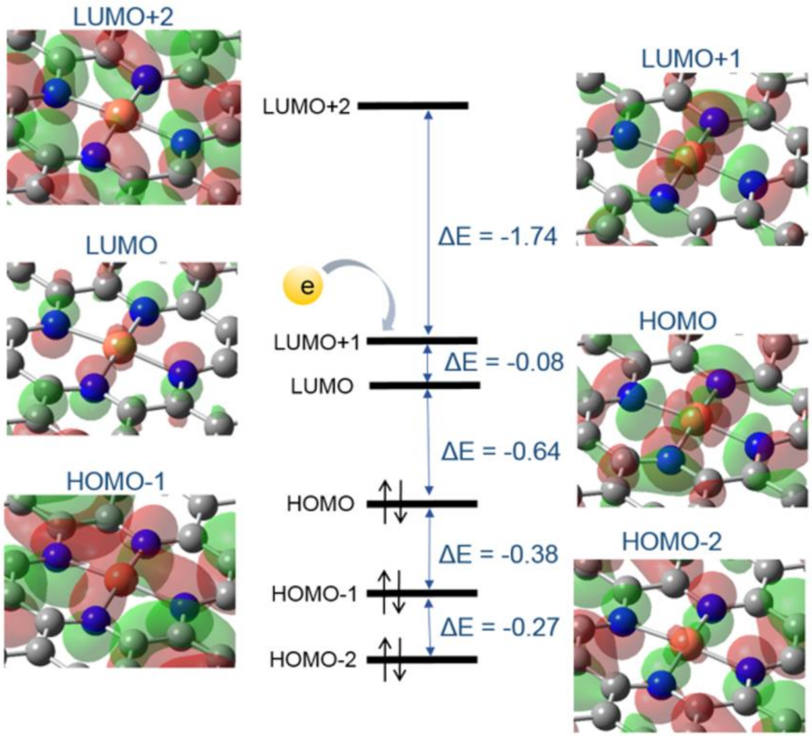

Figure S43. Calculation analysis. Frontier molecular orbitals of N-Cu SAC.

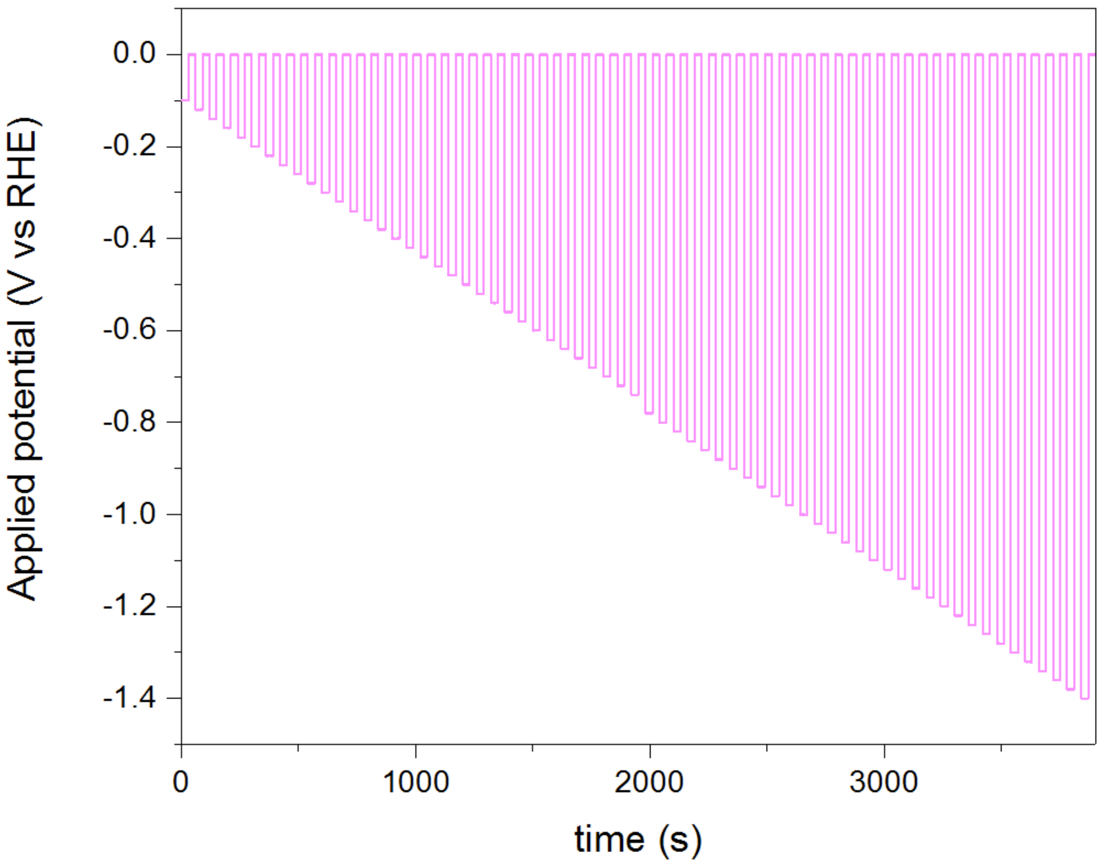

Figure S44. Pulse voltammetry protocol used for surface charge analysis. Pulse voltammetry protocol on metal SAC between 0 V and -0.1 V to -1.4 V potentials (non-iR corrected).

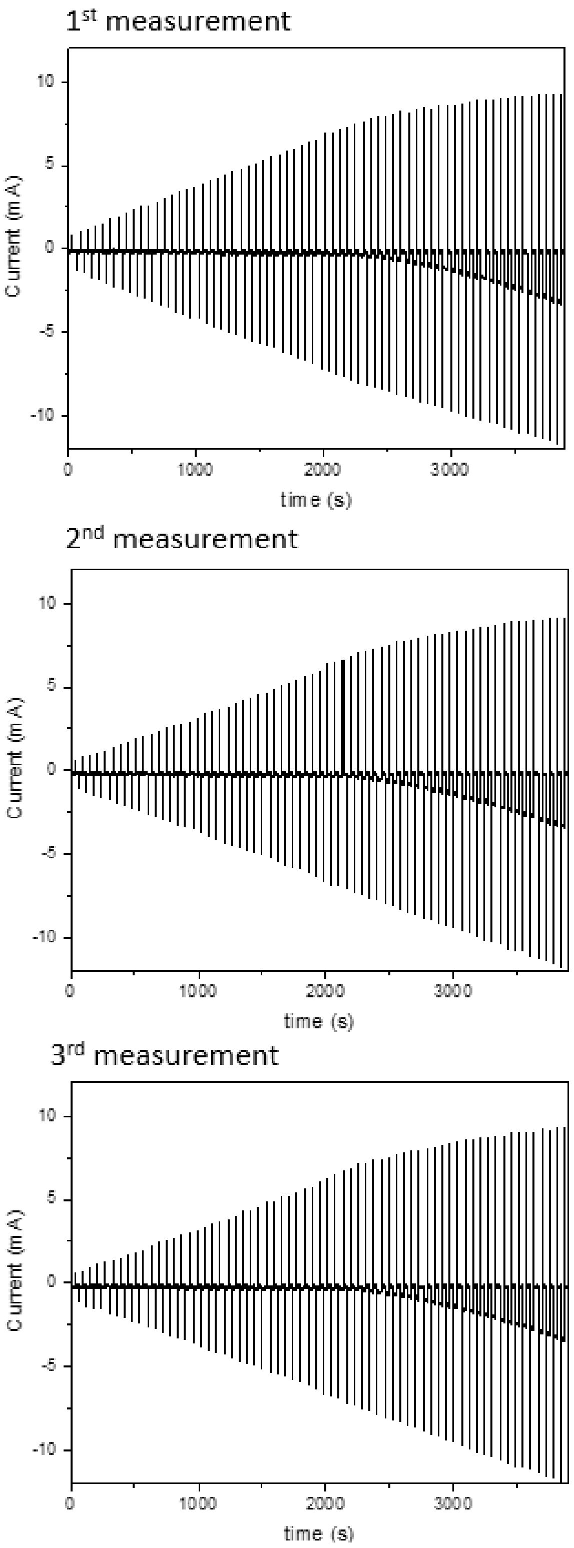

Figure S45. Current response to the voltage pulses. Current responses of N-C substrate from oxidative and reductive pulse potential with three different measurement times.

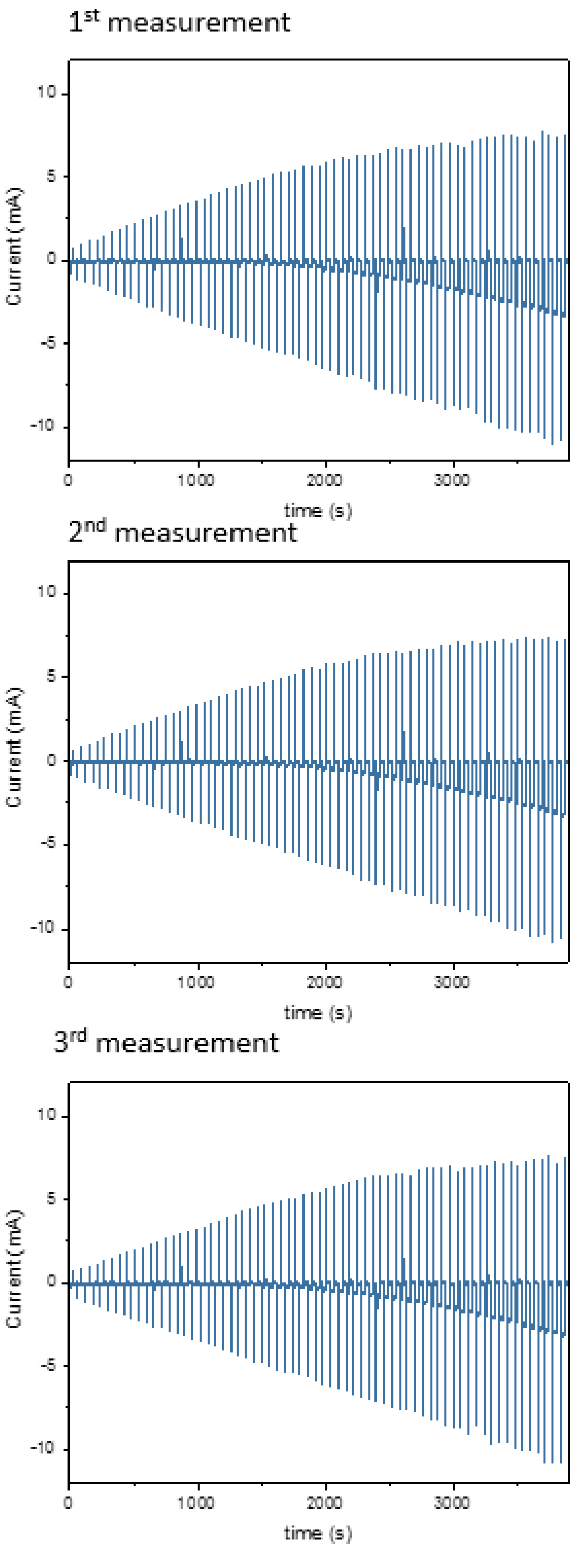

Figure S46. Current response to the voltage pulses. Current responses of N-Cu SAC from oxidative and reductive pulse potential with three different measurement times.

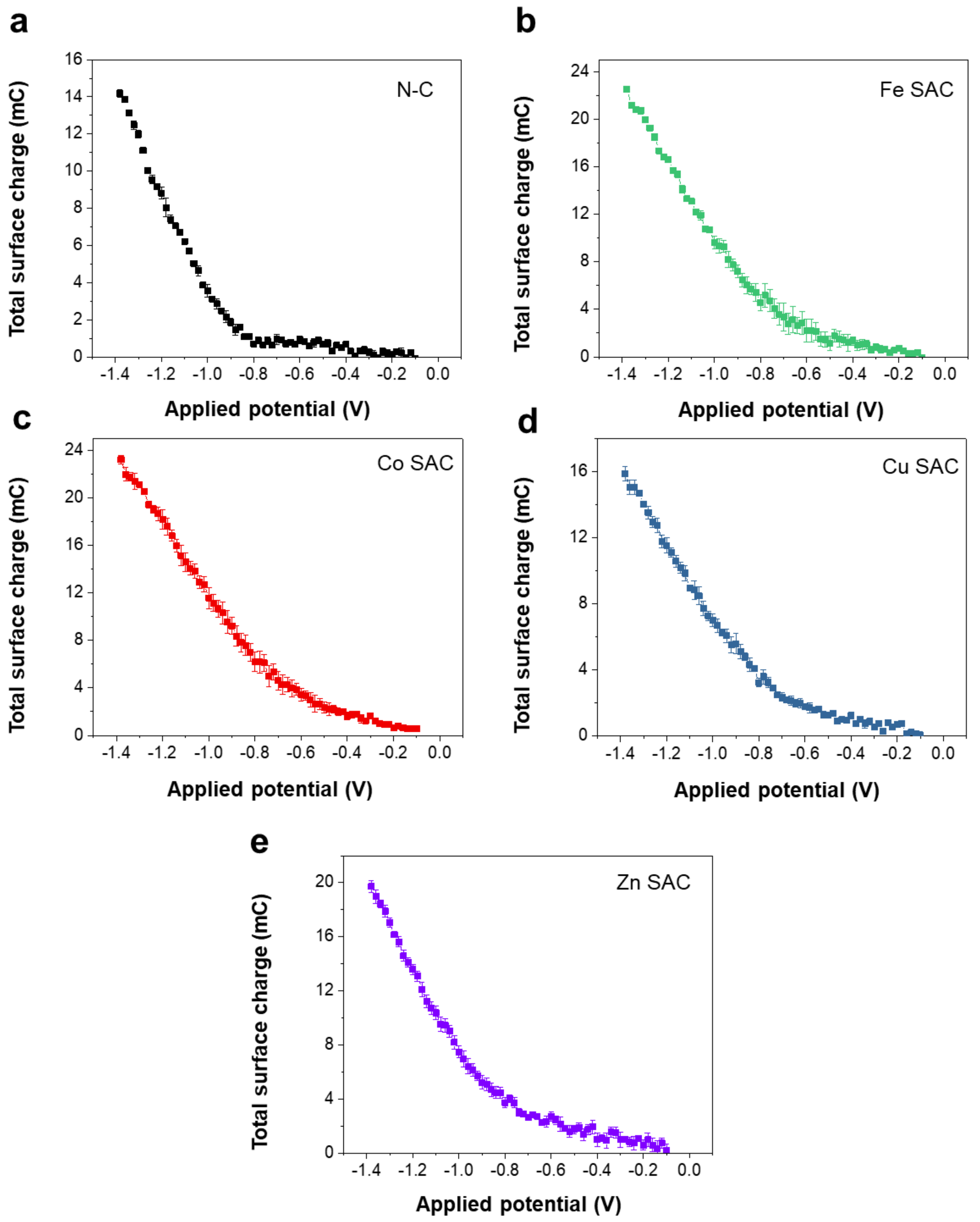

Figure S47. Calculated total surface charge. Total surface charge as a function of applied potentials from the pulse voltammetry of (a) N-C substrate, (b) Fe SAC, (c) Co SAC, (d) Cu SAC and (e) Zn SAC. Error bars represent the standard deviation of three independent measurements.

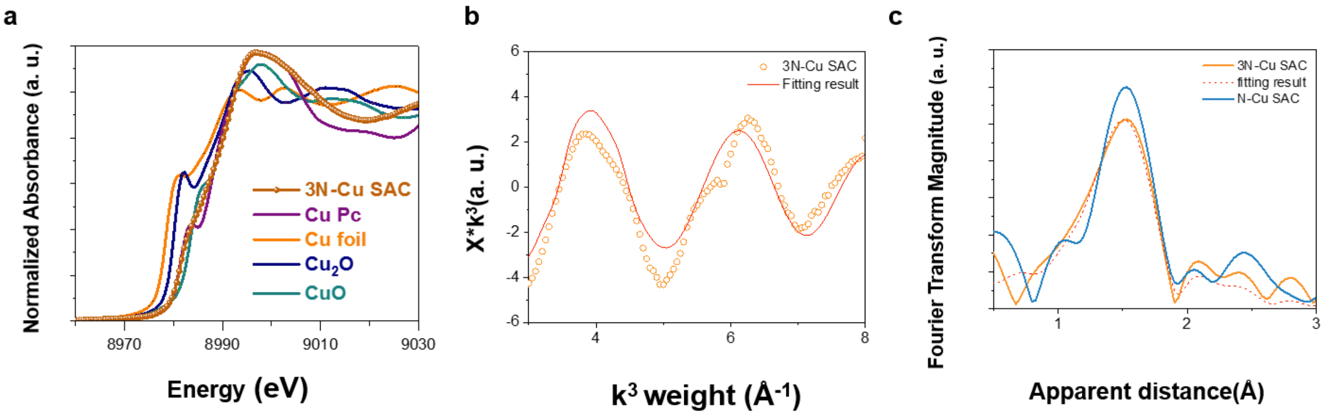

Figure S48. XAS analysis of 3N-Cu SAC. (a) XANES spectra of 3N-Cu SAC with various references.(b) Fitting results of (b) *k*-space and (c) R-space of Cu *K*-edge EXAFS spectra of 3N-Cu SAC as compared with N-CuSAC.

Note: EXAFS spectra suggest the single-atom nature of the as-prepared 3N-Cu SAC, and corresponding fitting results validate that the Cu ions are coordinated by three N, indicating the low-coordinated configuration of 3N-Cu SAC.

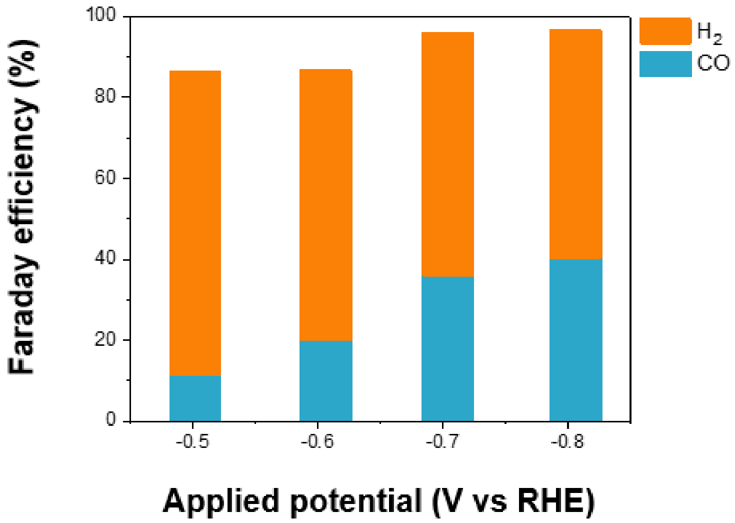

Figure S49. Electrochemical CO_2RR performance of 3N-Cu SAC. Potential-dependent product profile on the 3N-Cu SAC for CO_2RR.

Note: It was observed a higher CO selectivity on 3N-Cu SAC relative to that of N-Cu SAC at low cathodic potentials, which confirms that the low-coordinated configuration is more reactive for the CO_2-to-CO conversion.

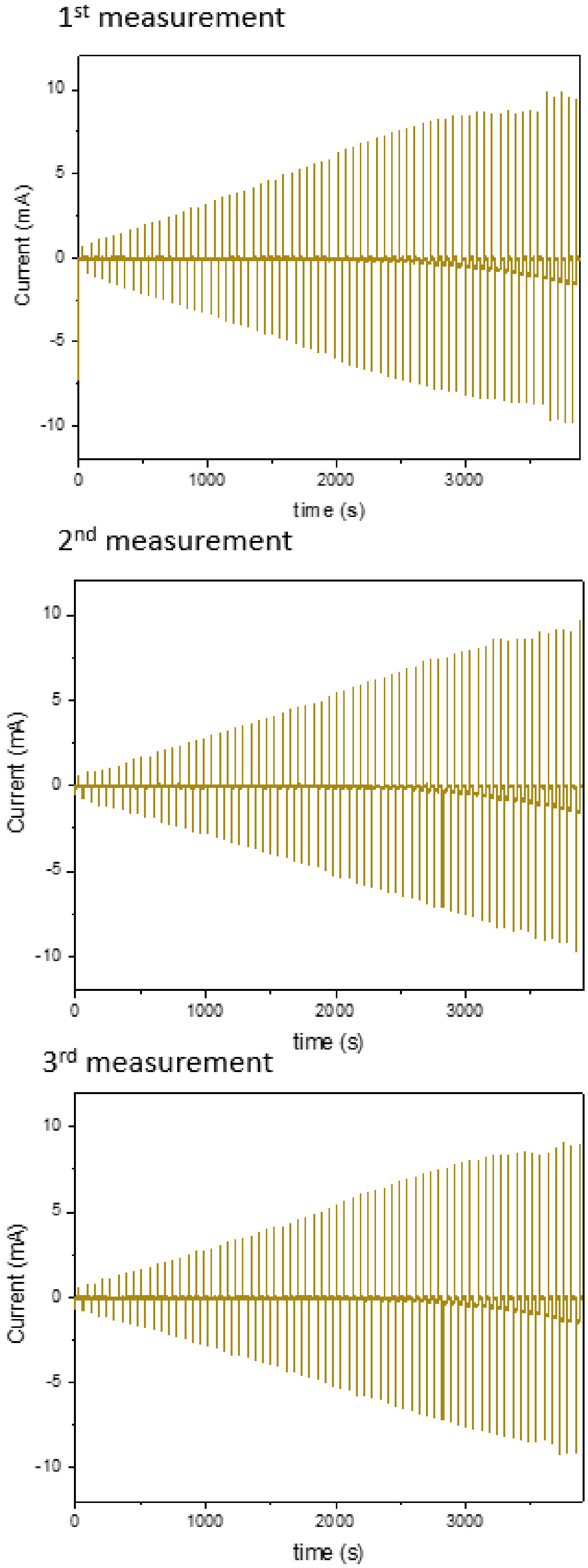

Figure S50. Current response to the voltage pulses. Current responses of 3N-Cu SAC from oxidative and reductive pulse potential with three different measurement times.

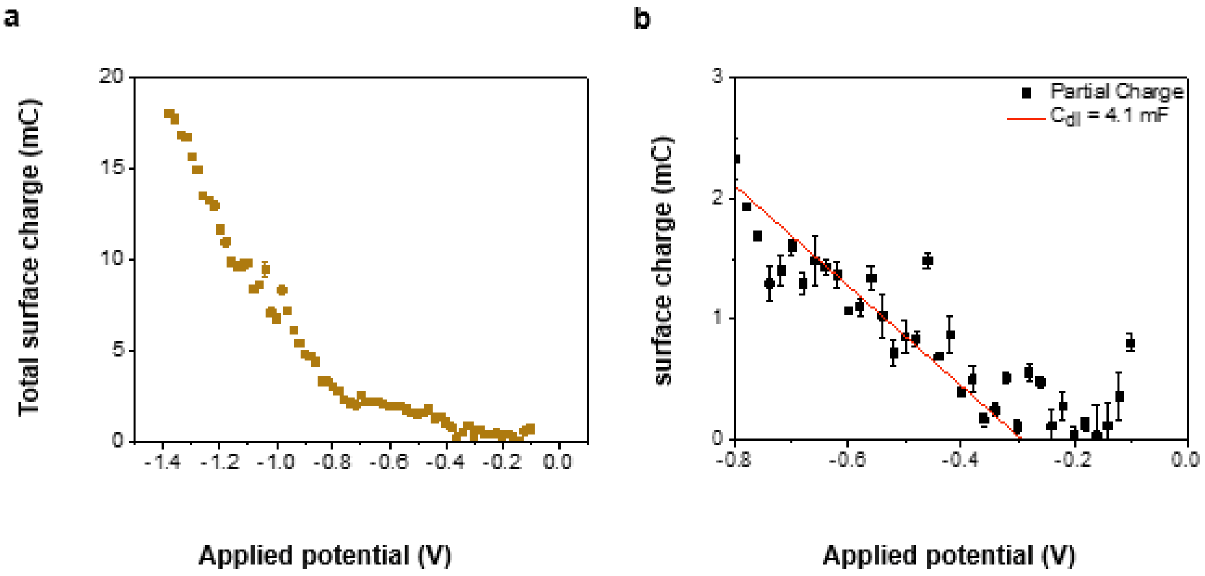

Figure S51. Surface charge analysis of 3N-Cu SAC. Total surface charge (a) and partial surface charge (b) as a function of applied potentials from the pulse voltammetry of 3N-Cu SAC. Error bars represent the standard deviation of three independent measurements.

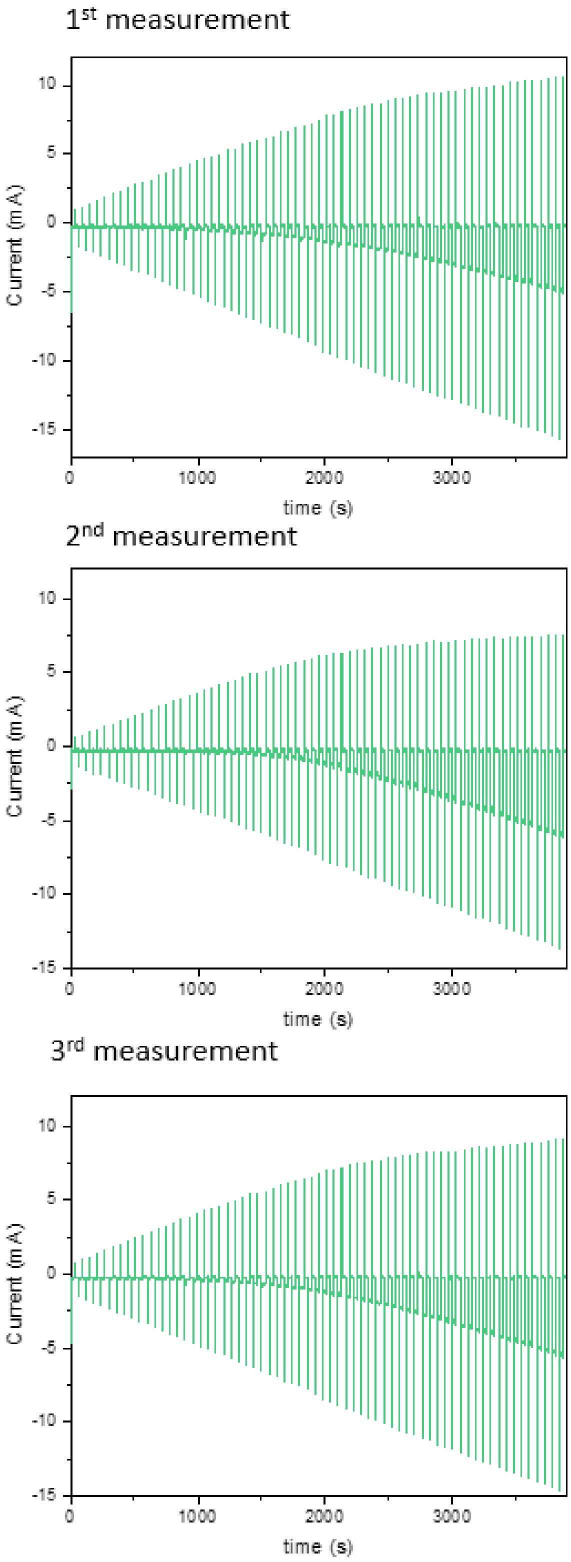

Figure S52. Current response to the voltage pulses. Current responses of Fe SAC from oxidative and reductive pulse potential with three different measurement times.

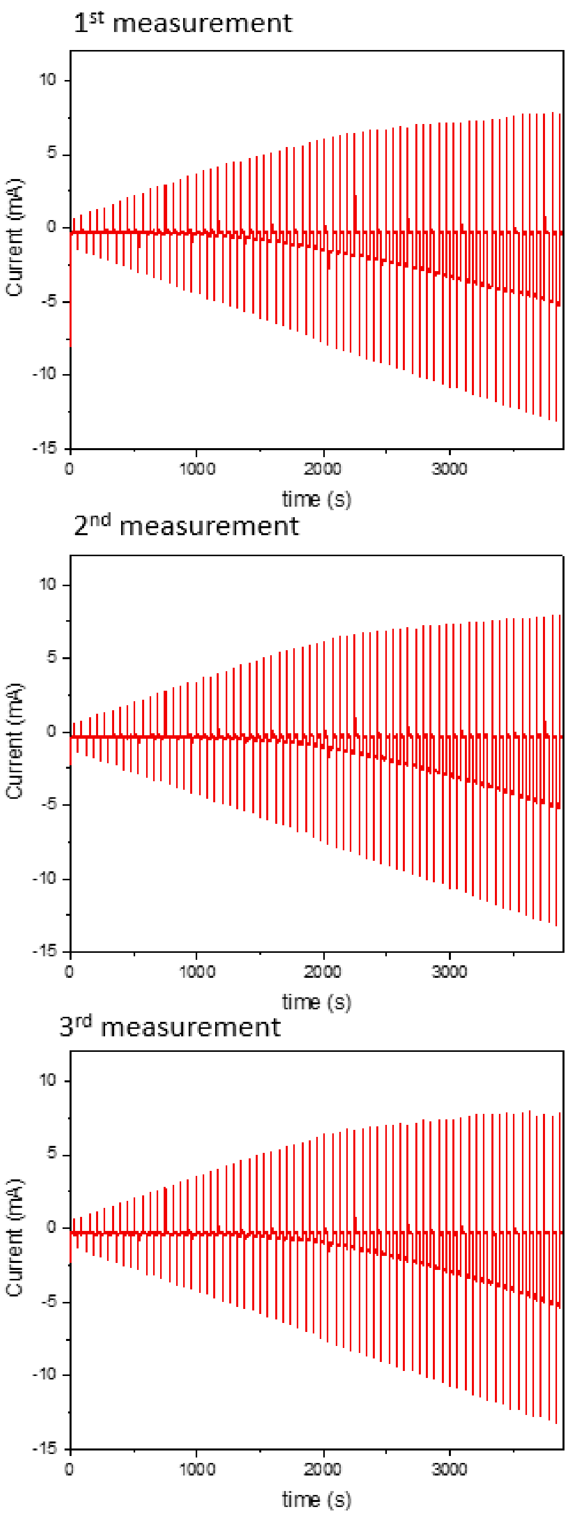

Figure S53. Current response to the voltage pulses. Current responses of Co SAC from oxidative and reductive pulse potential with three different measurement times.

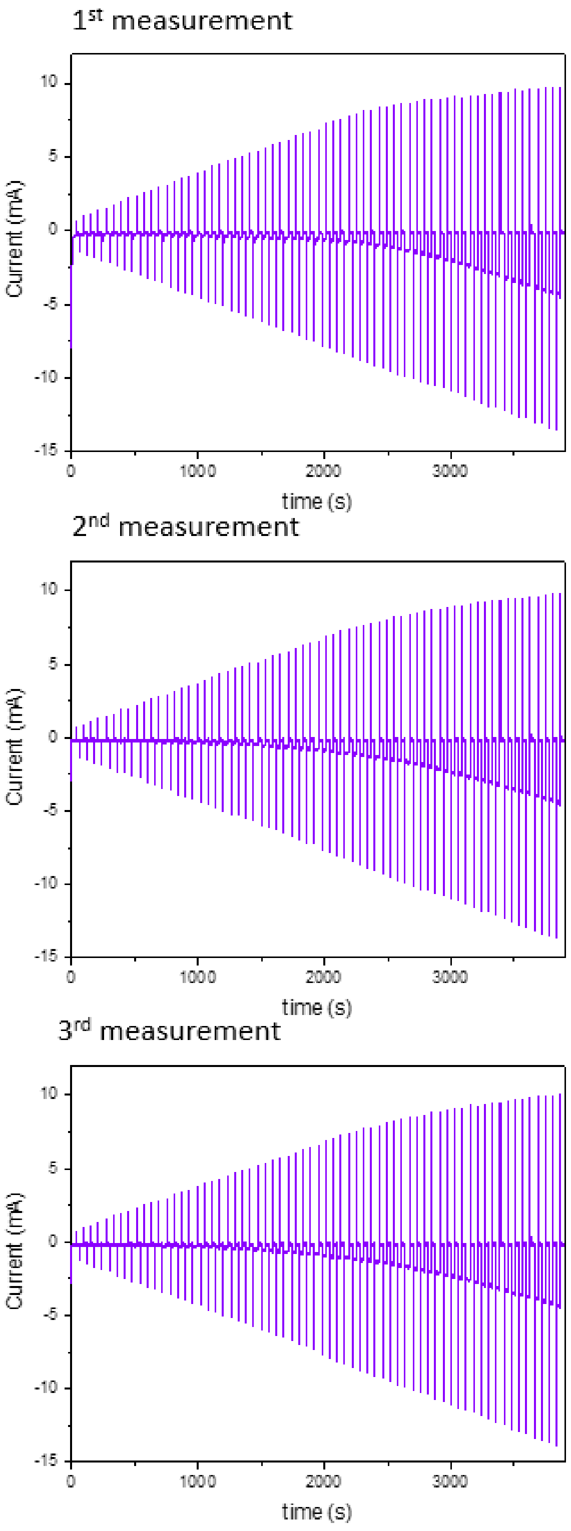

Figure S54. Current response to the voltage pulses. Current responses of Zn SAC from oxidative and reductive pulse potential with three different measurement times.

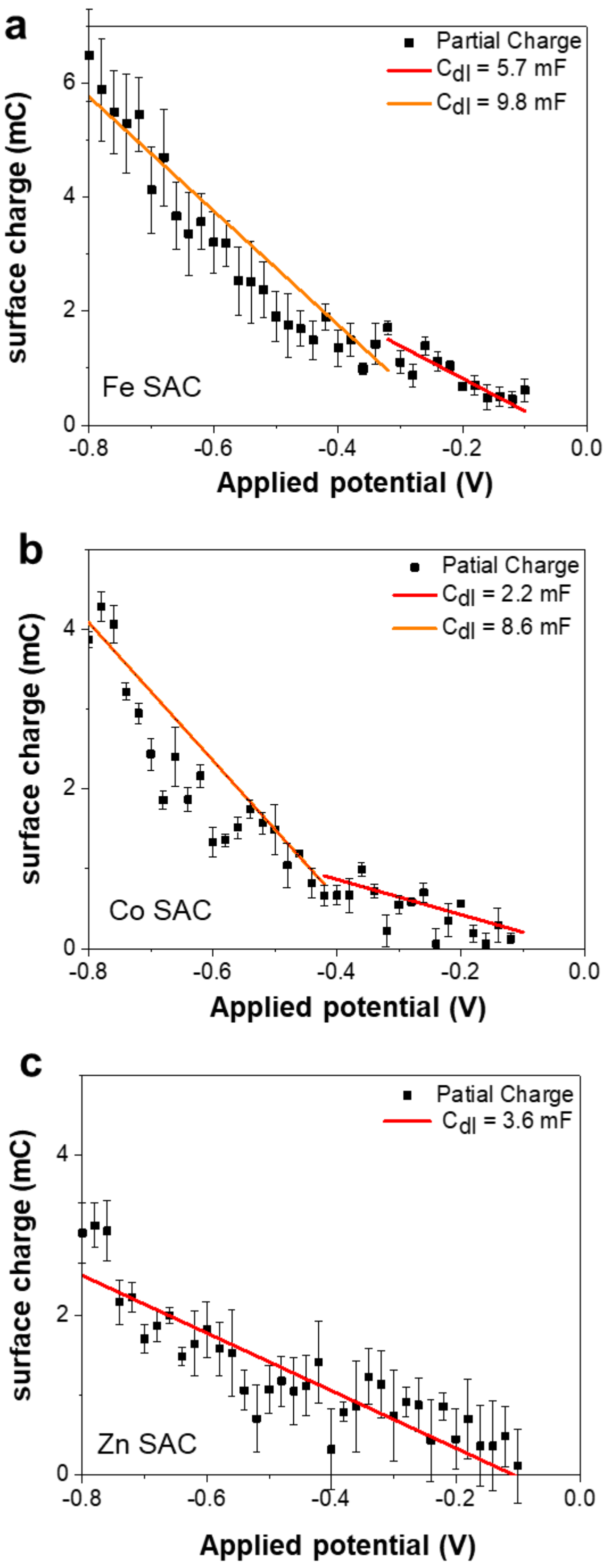

Figure S55. Surface charge analysis on different SACs. Partial charge as a function of applied potentials from the pulse voltammetry of (a) Fe SAC, (b) Co SAC, and (c) Zn SAC. Error bars represent the standard deviation of three independent measurements.

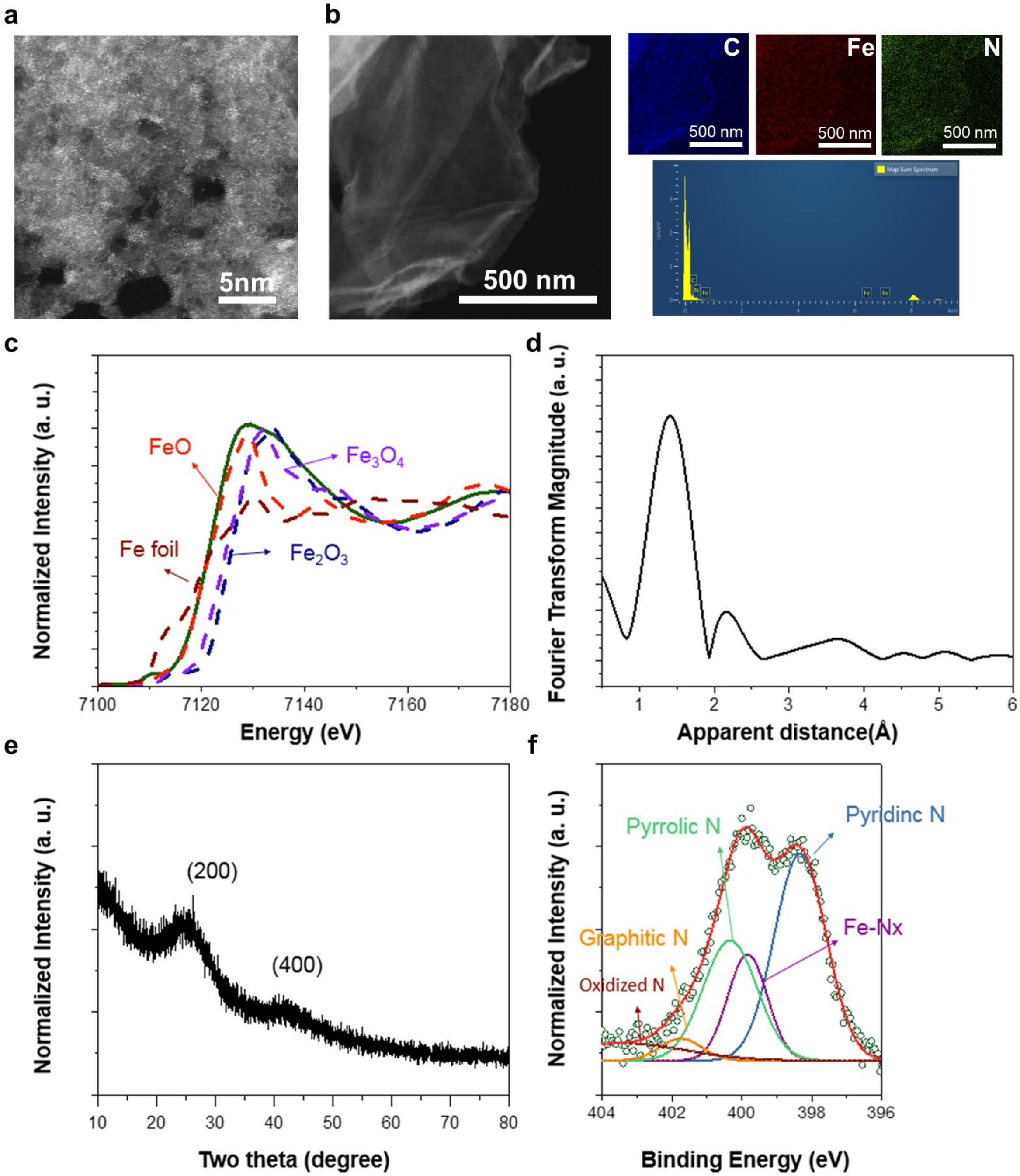

Figure S56. Physical characterizations for Fe SAC. (a) Aberration-corrected HAADF-STEM image. (b) STEM image, corresponding EDX mapping images and energy distribution. (c) XANES spectra with various references. (d) EXAFS spectrum. (e) XRD pattern. (f) N 1*s* XPS spectrum.

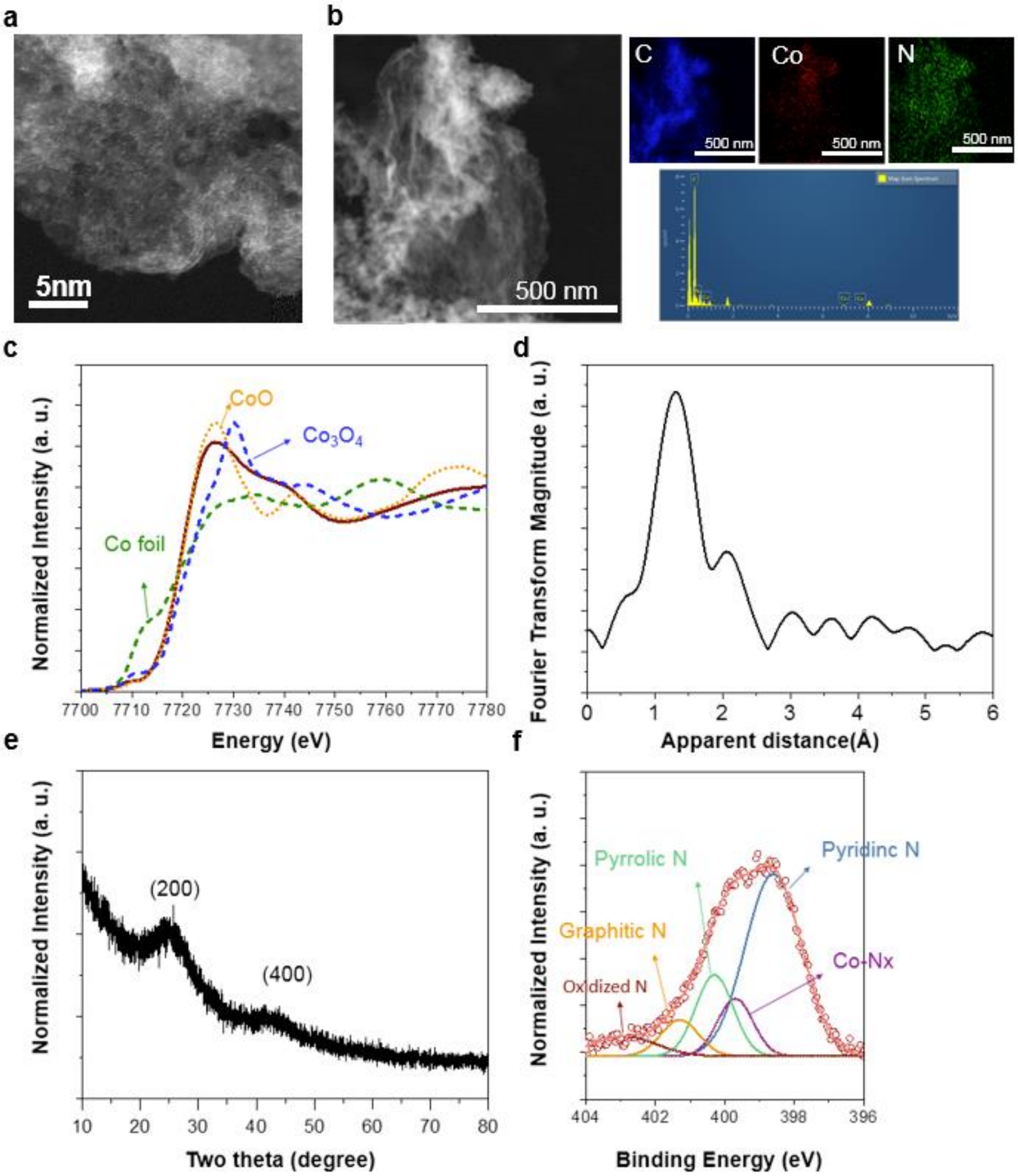

Figure S57. Physical characterizations for Co SAC. (a) Aberration-corrected HAADF-STEM image. (b) STEM image, corresponding EDX mapping images and energy distribution. (c) XANES spectra with various references. (d) EXAFS spectrum. (e) XRD pattern. (f) N 1*s* XPS spectrum.

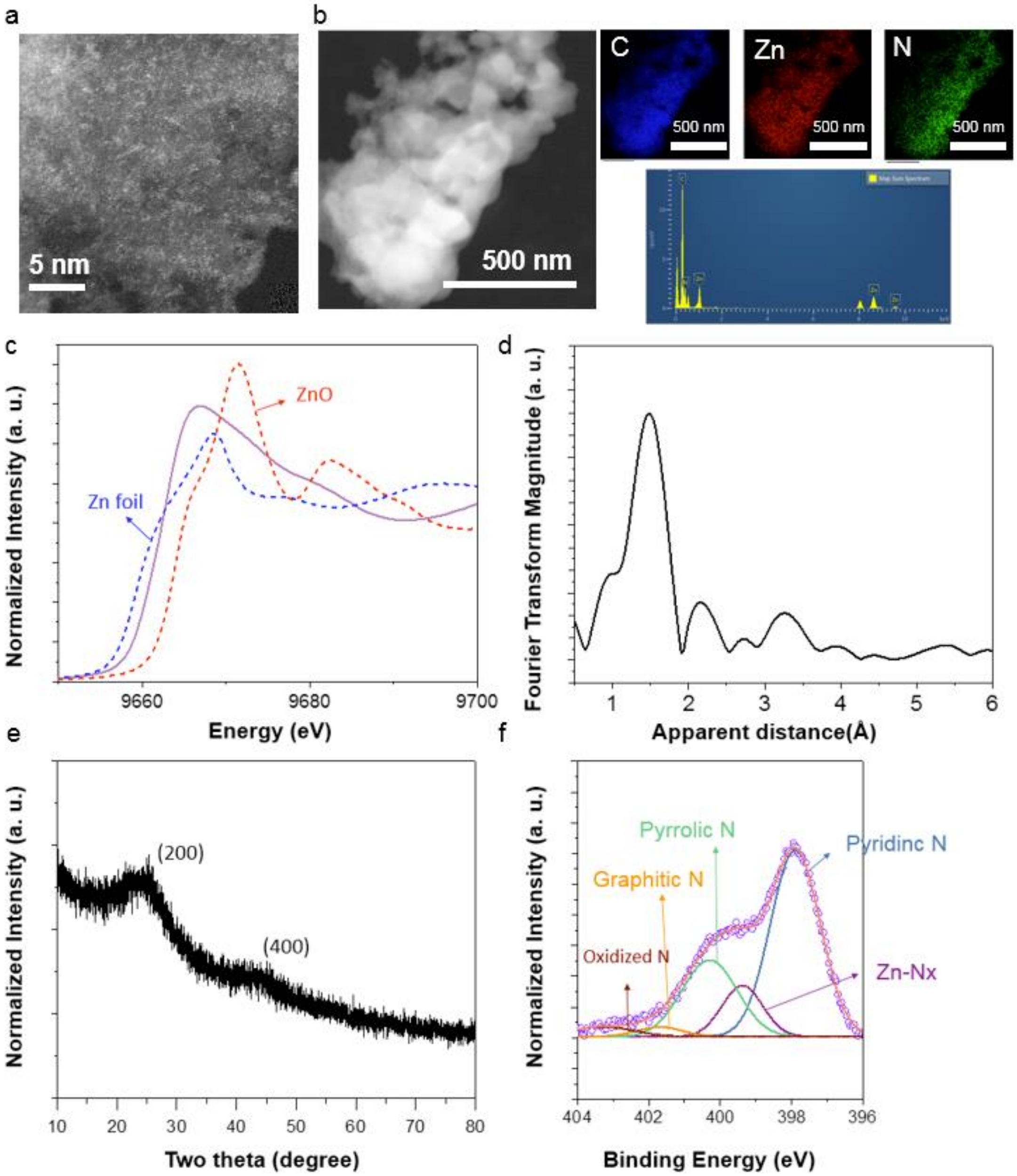

Figure S58. Physical characterizations for Zn SAC. (a) Aberration-corrected HAADF-STEM image. (b) STEM image, corresponding EDX mapping images and energy distribution. (c) XANES spectra with various references. (d) EXAFS spectrum. (e) XRD pattern. (f) N 1*s* XPS spectrum.

Note: Aberration-corrected HAADF-STEM images of all SACs showed many bright dots with the size of about 1 Å, which represented the existence of atomically dispersed metal atoms on the carbon support. The EDX mapping demonstrated the homogeneous distribution of Fe, Co and Zn. Powder XRD patterns only exhibited two broad reflection peaks typical for the nanometric graphitic carbon, excluding the presence of metal-containing nanoparticles in the samples. EXAFS spectra exhibited a main first-shell

scattering of metal-N at 1.4 Å and a small second-shell scattering of metal-C at 2.1 Å (without phase correction), moreover, the metal-metal scattering paths were not detected, further validating the single atom nature of SACs. N 1s XPS spectra demonstrated that the metal ions were coordinated to N. All these results prove the successful synthesis of atomically dispersed metal SACs, which could be used as ideal platforms to investigate the dynamic configuration-selectivity correlations.

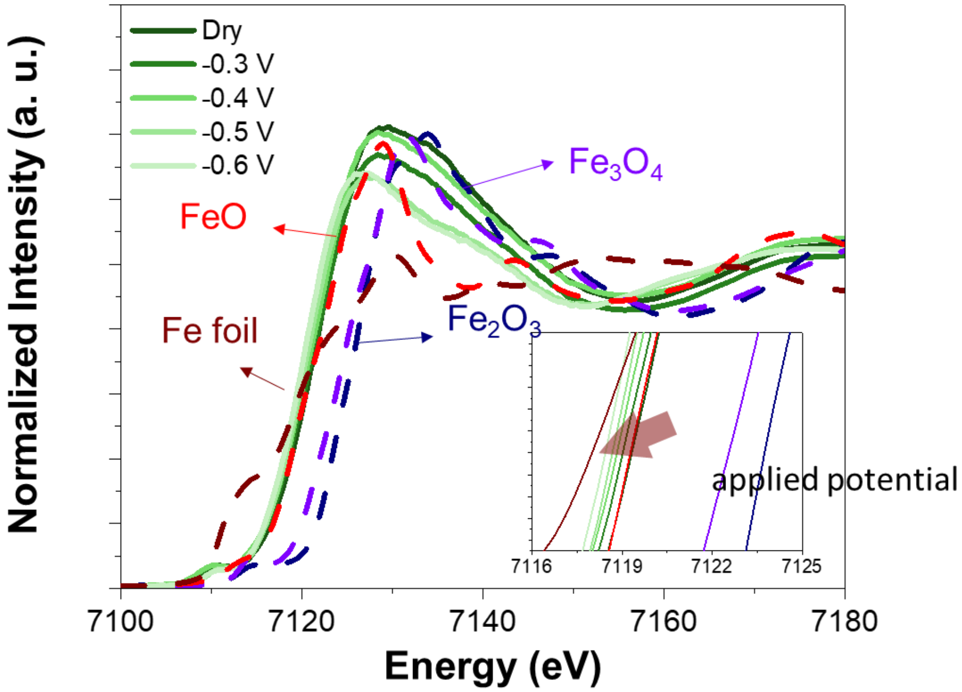

Figure S59. In situ XANES analysis on Fe SAC. In situ Fe K-edge XANES spectra of Fe SAC as dry powder and loaded on glassy carbon electrodes at -0.3~-0.6 V vs RHE, with the spectra of Fe foil, FeO, Fe_2O_3 and Fe_3O_4 as references. The insert showed the enlarged Fe K-edge XANES spectra.

Note: For the dry Fe SAC, the edge energy was close to that of FeO reference, indicating that the initial Fe ions were in the +2 oxidation state. When the applied potential was shifted from -0.3 to -0.6 V vs RHE, Fe *K*-edge underwent dynamic evolutions and shifted to lower energies, suggesting the gradual reduction of Fe^{2+} sites during the CO_2RR process. At -0.6 V vs RHE, the final Fe oxidation state was reduced to 0.4.

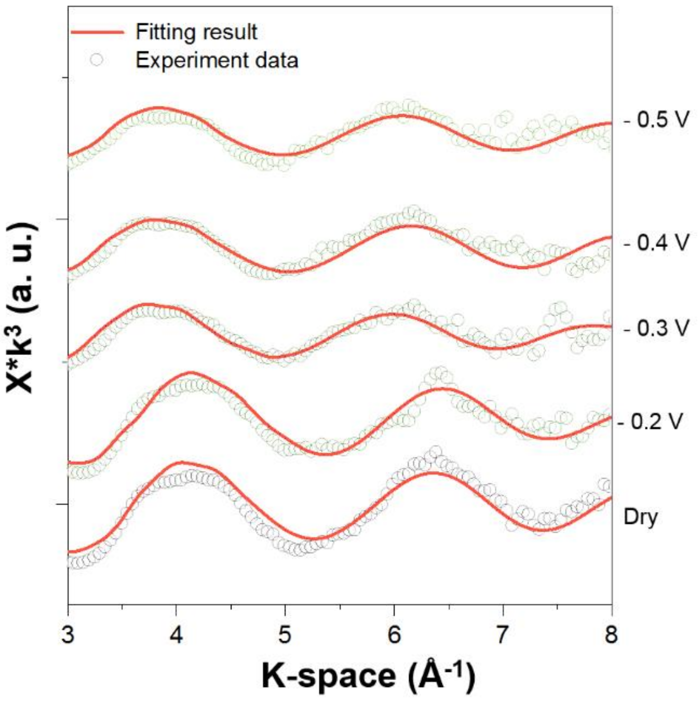

Figure S60. EXAFS fitting in k-space. Fitting results of in situ k-space Fe K-edge EXAFS spectra of Fe SAC at various applied potentials in 0.1 M CO_2-saturated $KHCO_3$. Fitting structural parameters are gathered in Table S7.

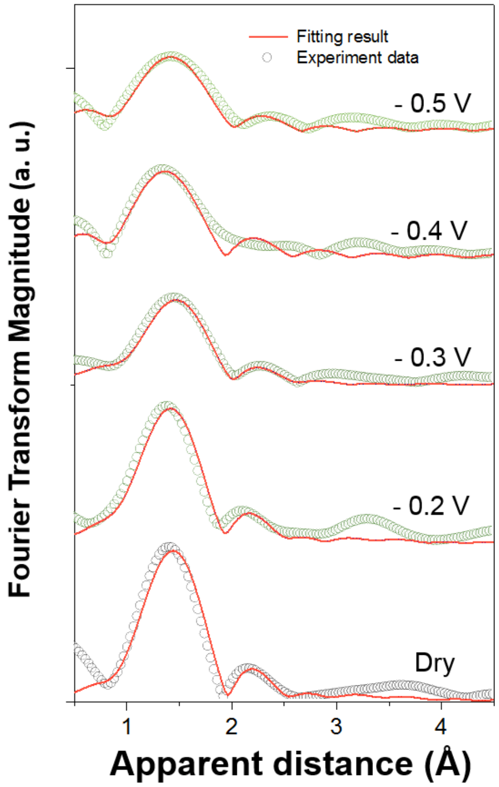

Figure S61. EXAFS fitting in R-space. Fitting results of in situ R-space Fe K-edge EXAFS spectra of Fe SAC at various applied potentials in 0.1 M CO_2-saturated $KHCO_3$. Fitting structural parameters are gathered in Table S7.

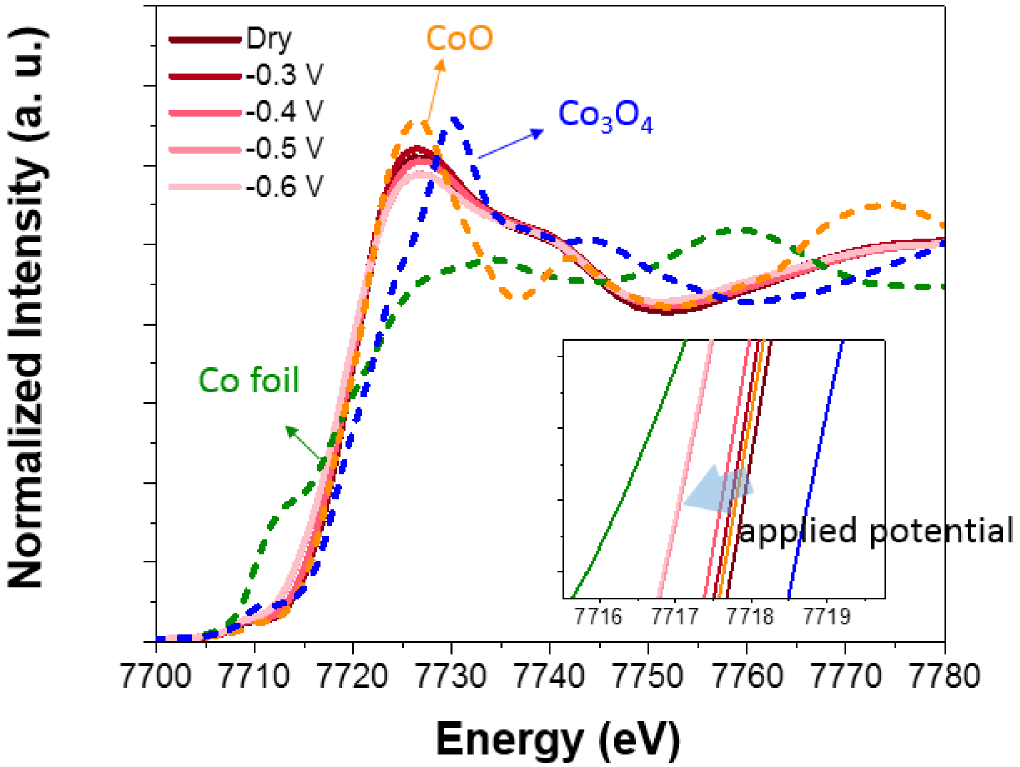

Figure S62. In situ XANES analysis on Co SAC. In situ Co K-edge XANES spectra of Co SAC as dry powder and loaded on glassy carbon electrodes at -0.3~-0.6 V vs RHE, with the spectra of Co foil, CoO and Co_3O_4 as references. The insert showed the enlarged Co K-edge XANES spectra.

Note: For the dry Co SAC, the edge energy was close to that of CoO reference, indicating that the initial Co ions were in the +2 oxidation state. When the applied potential was shifted from -0.3 to -0.6 V vs RHE, Co *K*-edge shifted to lower energies, suggesting the gradual reduction of Co^{2+} sites during the CO_2RR process. At -0.6 V vs RHE, the final Co oxidation state was reduced to 0.8.

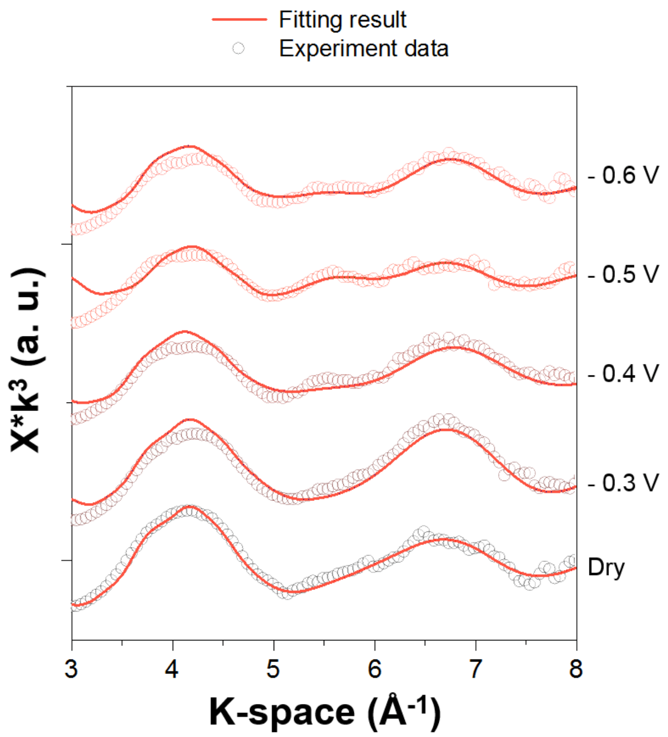

Figure S63. EXAFS fitting in k-space. Fitting results of in situ k-space Co K-edge EXAFS spectra of Co SAC at various applied potentials in 0.1 M CO_2-saturated $KHCO_3$. Fitting structural parameters are gathered in Table S8.

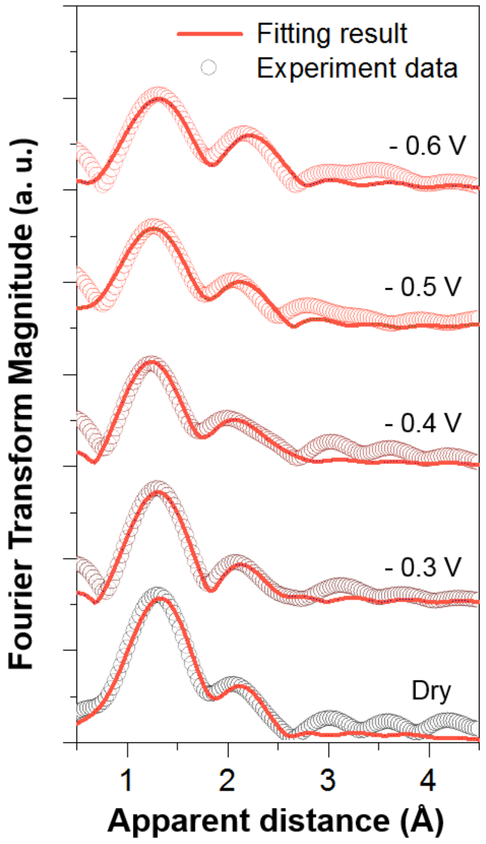

Figure S64. EXAFS fitting in R-space. Fitting results of in situ R-space Co K-edge EXAFS spectra of Co SAC at various applied potentials in CO_2-saturated 0.1M $KHCO_3$. Fitting structural parameters are gathered in Table S8.

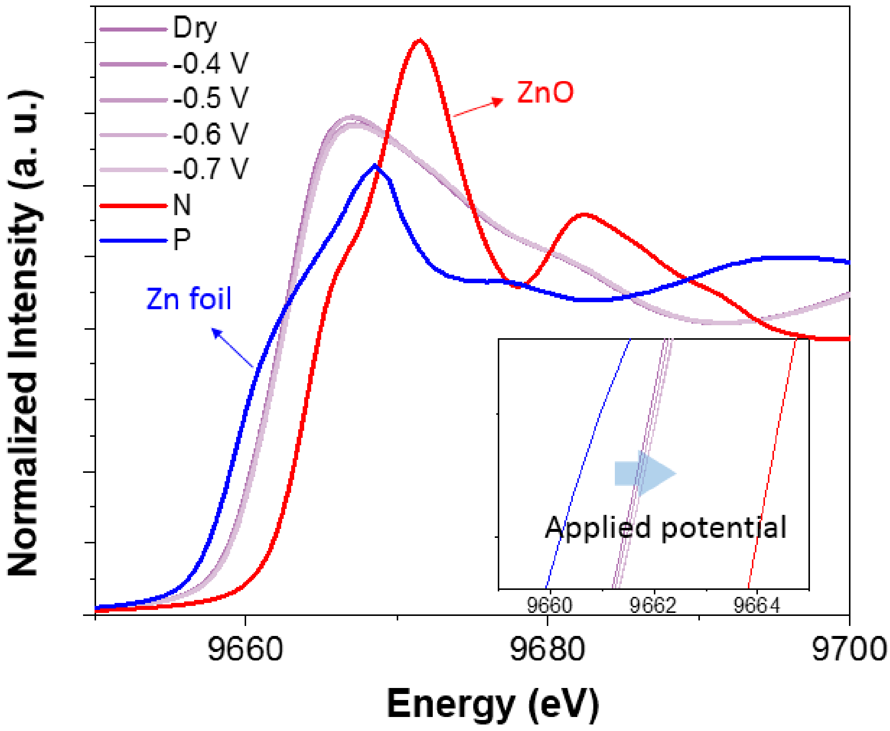

Figure S65. In situ XANES analysis on Zn SAC. In situ Zn K-edge XANES spectra of Zn SAC as dry powder and loaded on glassy carbon electrodes at -0.3~-0.6 V vs RHE, with the spectra of Zn foil and ZnO as references. The insert showed the enlarged Zn K-edge XANES spectra.

Note: For the dry Zn SAC, the edge energy was located between Zn foil and ZnO, indicating that the oxidation state of initial Zn ions was between 0 and +2. When the applied potential was shifted from -0.3 to -0.6 V vs RHE, Zn *K*-edge showed negligible changes during the CO_2RR process.

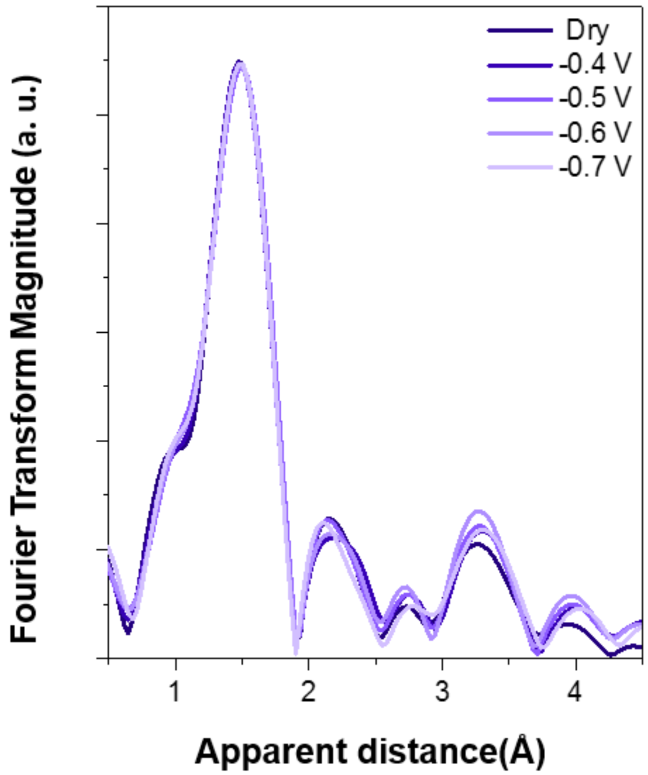

Figure S66. In situ EXAFS spectra. In situ Zn K-edge EXAFS spectra of Zn SAC at various applied potentials during CO_2 reduction.

Note: For Fe, Co and Zn SACs, all EXAFS spectra exhibited a main metal-N scattering at 1.4 Å (without phase correction), and moreover, the metal-metal scattering paths were not detected, which validates the single atom nature of SACs. Once the potentials were applied, coordination environment of metal sites in Fe and Co SACs underwent dynamic changes. Specifically, for the dry Fe SAC, Fe ions are initially coordinated by 4N at the first shell. Upon -0.3 V and more cathodic potentials, the coordination number of Fe-N scattering reduced to 2, forming Fe-N_2 moieties during CO_2RR. For the dry Co SAC, Co ions are initially coordinated by 4N at the first shell. Upon -0.3 V, the coordination number of Co-N scattering reduced to 3. With further increasing the potential (< -0.4 V), Co ions were observed to be coordinated by 2N. As compared with Fe and Co SACs that showed the decline in coordination number of Fe/Co-N path, Zn SAC did not undergo dynamic changes in the coordination environment, which remained Zn-4N configuration over the course of CO_2RR. Note that the decline in coordination number of Fe/Co-N path in Fe and Co SACs is expected to be closely correlated to the CO FE.

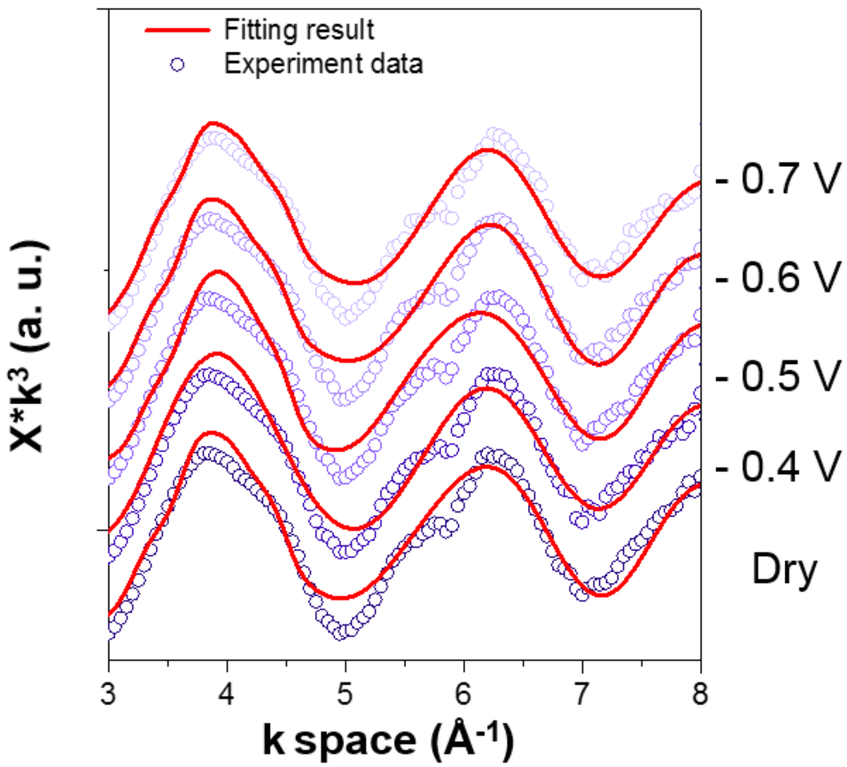

Figure S67. EXAFS fitting in k-space. Fitting results of in situ k-space Zn K-edge EXAFS spectra of Zn SAC at various applied potentials in 0.1 M CO_2-saturated $KHCO_3$. Fitting structural parameters are gathered in Table S9.

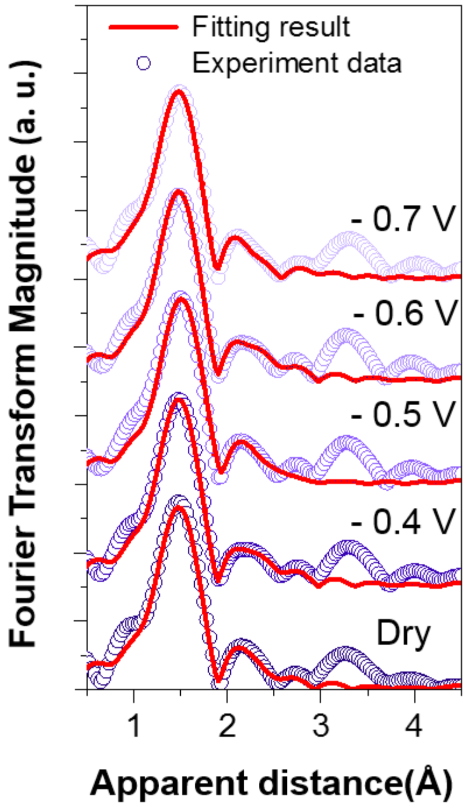

Figure S68. EXAFS fitting in R-space. Fitting results of in situ R-space Zn K-edge EXAFS spectra of Zn SAC at various applied potentials in CO_2-saturated 0.1M $KHCO_3$. Fitting structural parameters are gathered in Table S9.

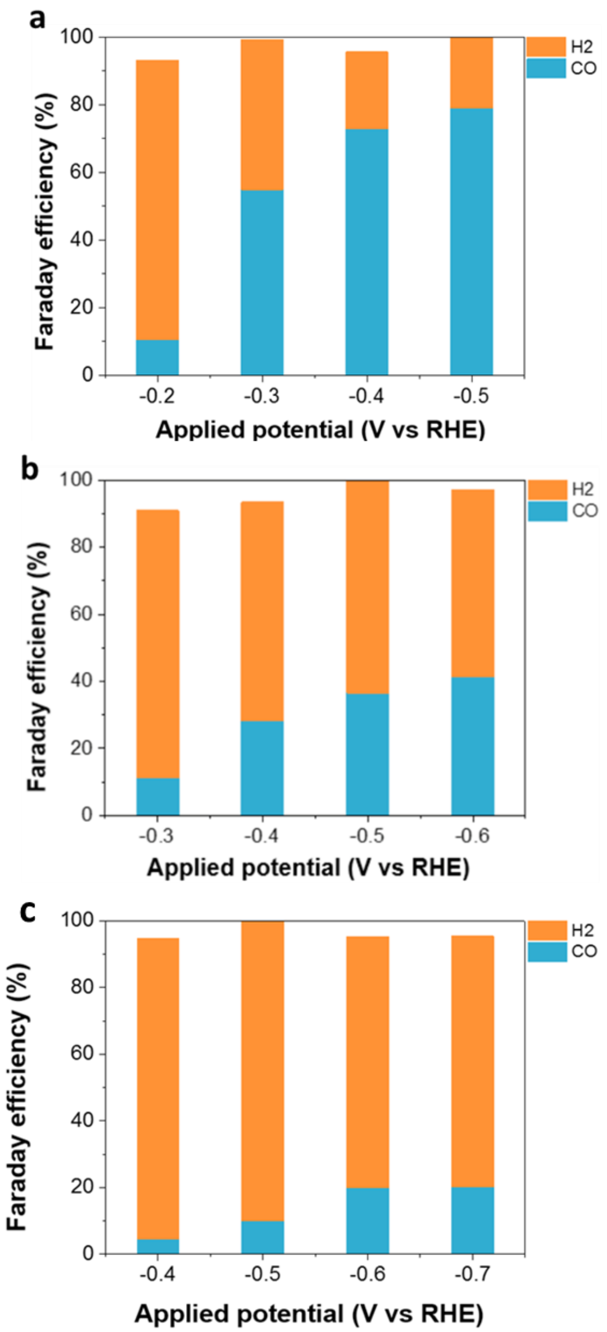

Figure S69. Electrochemical CO_2RR performance of different SACs. Potential-dependent product profile on (a) Fe SAC, (b) Co SAC and (c) Zn SAC for CO_2RR.

Note: Over the applied potential range, for Fe and Co SACs, CO and H_2 were identified as the major reaction products, and moreover, FE of CO was observed to increase with increasing the applied potentials, while H_2 FE accordingly decreased. It was further revealed that the most significant enhancement of CO FE in the Fe and Co SACs was observed at -0.3 V and -0.4 V vs RHE, respectively. By combining in situ EXAFS results (Figure 4e-g), such CO FE enhancement was found to coincide well with the formation of

low-coordinated configuration of SACs. By contrast, Zn SAC exhibited the considerably low selectivity toward CO with unchanged atomic configuration.

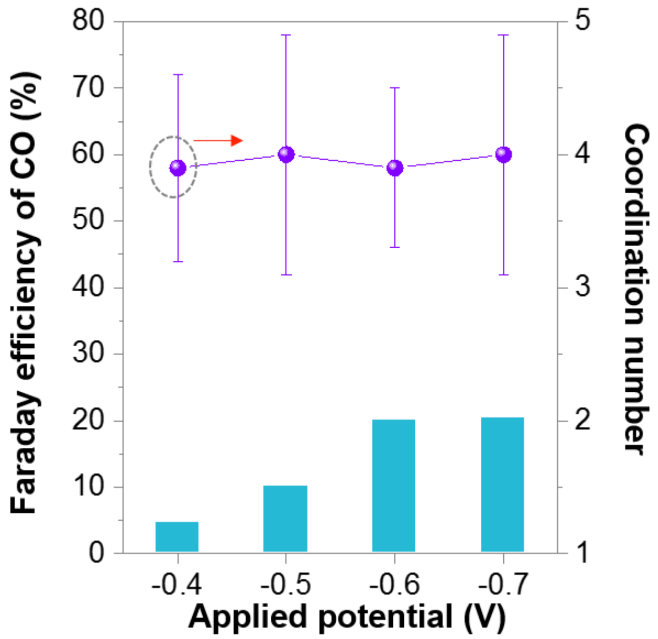

Figure S70. Correlation between CO selectivity and coordination number for Zn SAC. Potential-dependent CO_2RR product profile and corresponding coordination number extracted from in situ *K*-edge EXAFS fitting for Zn SAC. Error bars represent the standard deviation of three independent measurements.

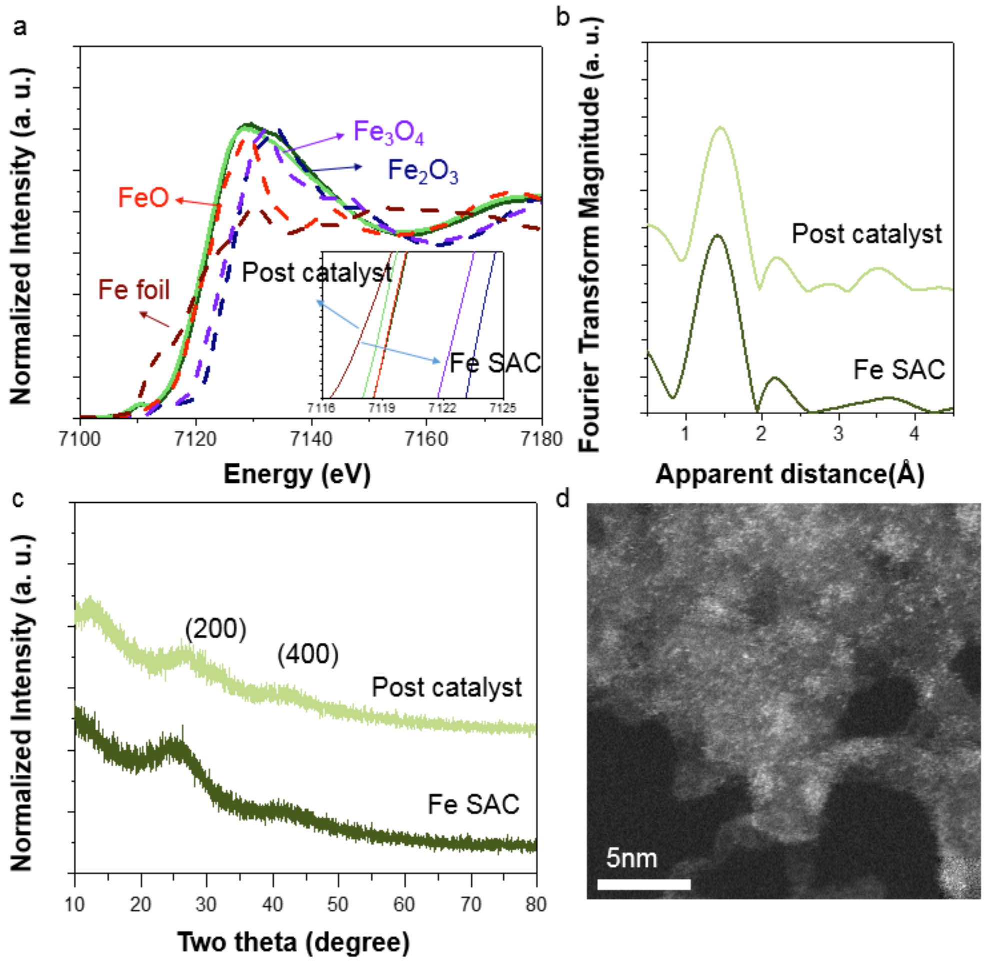

Figure S71. Physical characterizations for as-prepared and post-test sample of Fe SAC. (a) XANES spectra with various references. (b) Corresponding EXAFS spectra. (c) XRD patterns. (d) Aberration-corrected HAADF-STEM images of post-test sample.

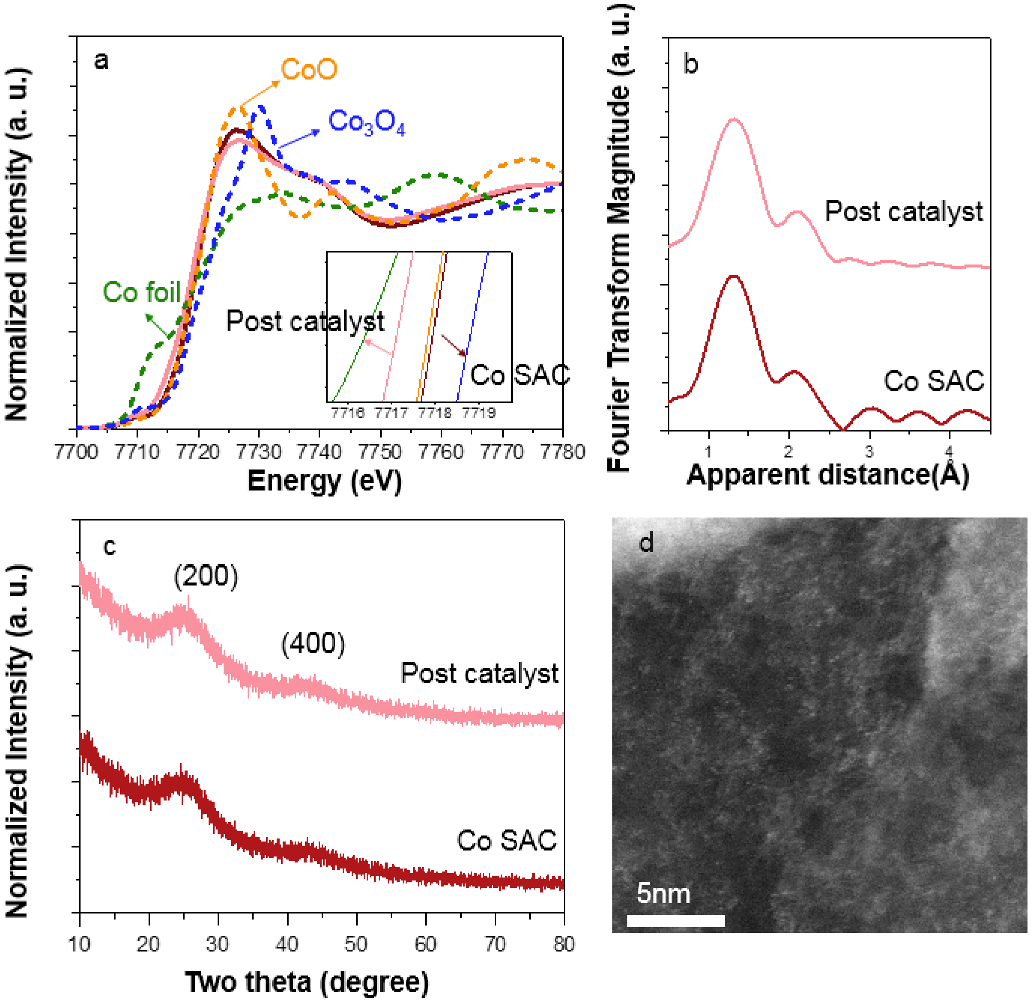

Figure S72. Physical characterizations for as-prepared and post-test sample of Co SAC. (a) XANES spectra with various references. (b) Corresponding EXAFS spectra. (c) XRD pattern. (d) Aberration-corrected HAADF-STEM images of post-test Co SAC.

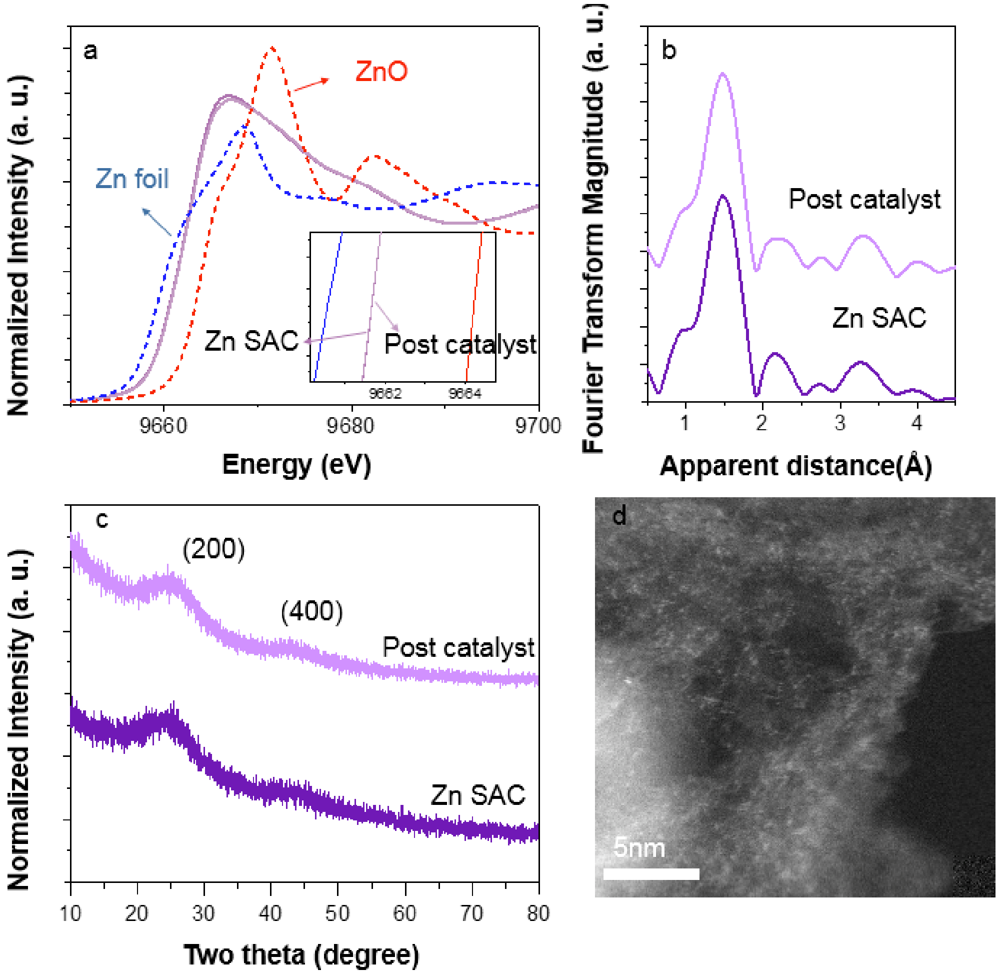

Figure S73. Physical characterizations for as-prepared and post-test sample of Zn SAC. (a) XANES spectra with various references. (b) Corresponding EXAFS spectra. (c) XRD pattern. (d) Aberration-corrected HAADF-STEM images of post-test Zn SAC.

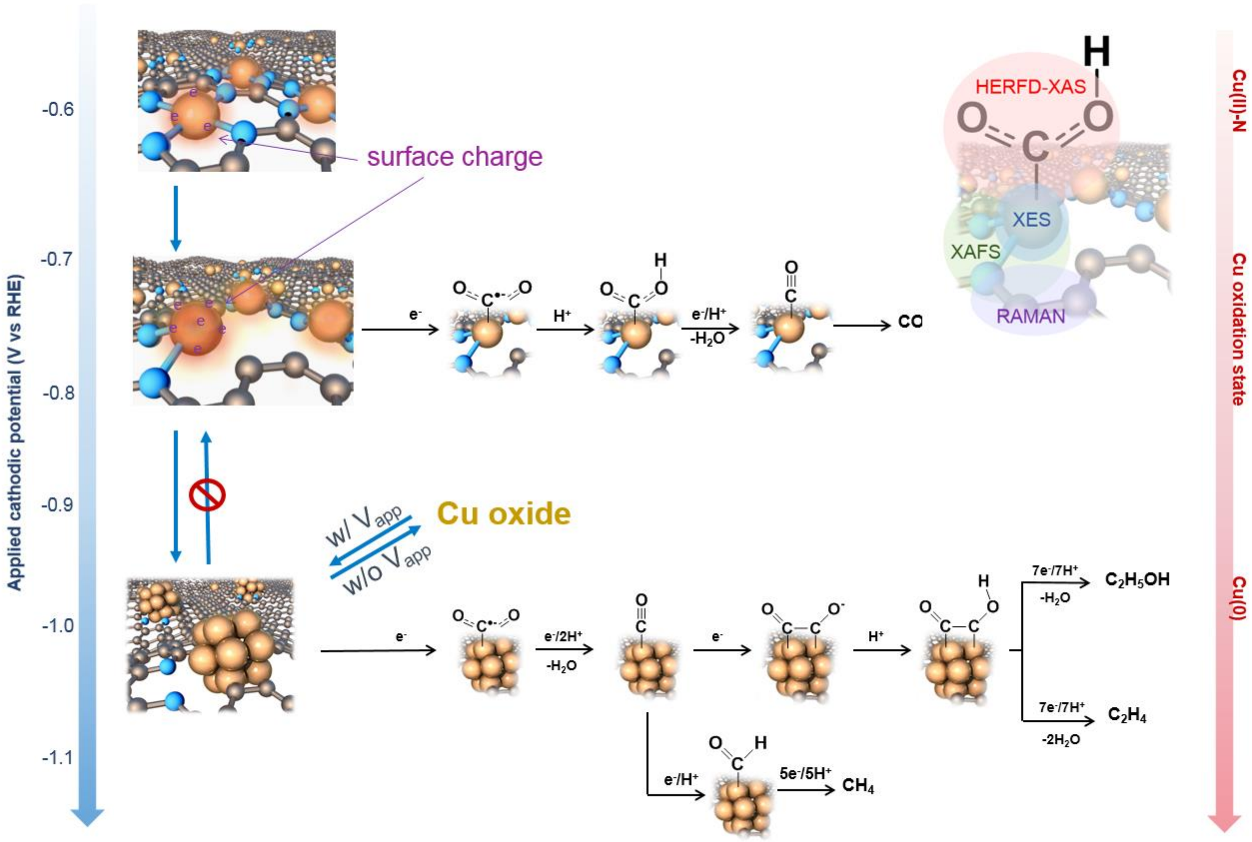

Figure S74. Mechanistic understanding of CO_2RR on Cu SAC. Schematic of dynamic restructuring process in N-Cu SAC at applied cathodic potentials with proposed CO_2RR pathways toward CO, C2 products and methane.

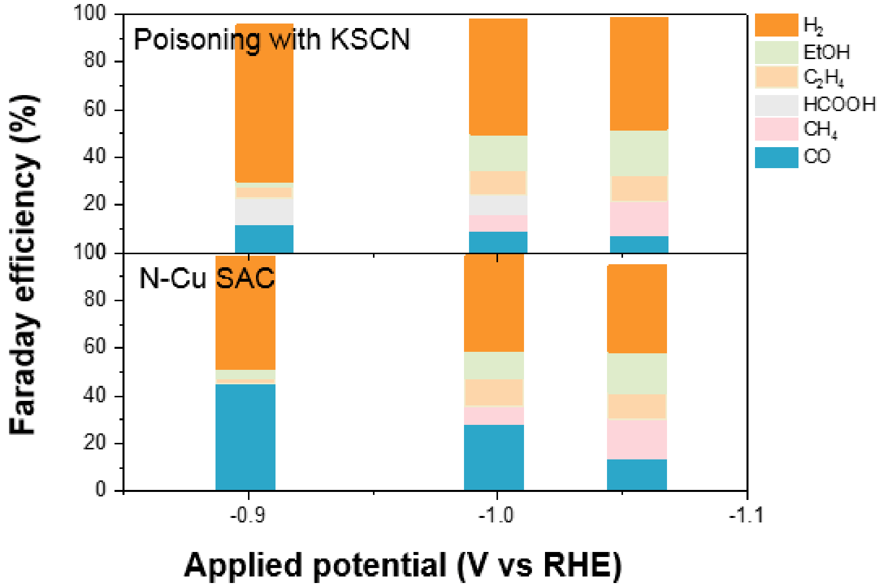

Figure S75. KSCN poisoning analysis. Potential-dependent product profile on the N-Cu SAC toward CO_2RR with and without KSCN poisoning.

Note: It can be clearly seen that with the presence of KSCN the CO selectivity of N-Cu SAC dramatically decreases from 45% to 11% at -0.9 V. In contrast, the CO selectivity exhibits a medium decrease from 28% to 9% at -1.0 V, and displays a negligible attenuation at -1.1 V. Considering that the SCN− groups can effectively block Cu single atoms and lead to significant performance decay, one can conclude that the amount of Cu single-atom sites increasingly decreased (Cu clusters gradually formed) with increasing applied potentials. Such KSCN poisoning experiments provide another evidence on the potential-driven restructuring of N-Cu SAC during CO_2RR.

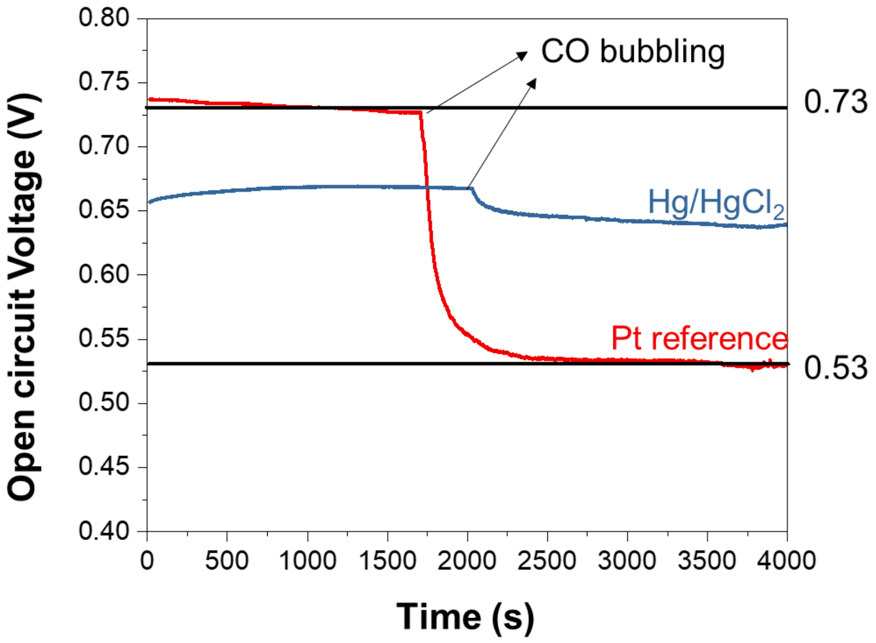

Figure S76. The calibration of Pt quasi-reference electrode. Variations of the open-circuit potential with time measured in 0.1M $KHCO_3$ solution saturated with hydrogen and continuous CO bubbling.

Note: Given that the primary product of CO_2RR on SACs is CO, the CO poisoning effect on the open-circuit potential drift during reactions must be taken into account since CO is a strong poison for Pt surfaces. To correctly calibrate Pt quasi-reference electrode to its RHE, we used "HydroFlex Standard Hydrogen Reference Electrode" as reference electrode for the calibration process. Specifically, in a typical three electrode system, one Pt wire was used as the working electrode, HydroFlex electrode as the reference electrode and another Pt wire as the counter electrode. To detect the variations of the open-circuit potential with time, the experiments were simulated in the following two environments: i) 0.1M $KHCO_3$ solution saturated with hydrogen only; ii) the above $KHCO_3$ solution injected with continuous CO bubbling. As illustrated in Figure, for Pt reference, it revealed that the open-circuit potential could remain 0.73 V in the H_2-saturated electrolyte. Once CO injection was started, we observed that the open-circuit potential evidently decreased to 0.53 V. In this experiment, it was found that the potential drift occurred in the period of 1700 s $\leq$ t $\leq$ 2500 s with the time duration up to 800 s. For in situ TEM measurements, there was no CO production in the chip space at beginning, thus the open-circuit potential was 0.73 V. As the CO_2RR proceeded at applied potentials, the poisoning effect of the produced CO significantly induced the open-circuit potential drift. Especially, the structural transformation into clusters can be captured by TEM within 200 s (Figure 1e), which is quite shorter than the time duration (800 s) required by the drifting potential for achieving a steady state. Thus, the linear fitting for the changes in

open-circuit potential (vs Pt reference) within 200 s as CO injection was started should be conducted to realize a true calibration vs an actual RHE. Through the calculation, the linear fitting curve was shown as equation, $E_{RHE} = E_{pt\ ref} - 0.000304 \times t(s) + 0.68V$ (Fig. 1h and i).

In the case of TEM measurement for N-Cu SAC at -1.1 V vs RHE, we applied -1.83 V vs Pt reference considering the open-circuit potential of 0.73 V vs Pt reference. As time goes on, in fact, the potentials vs an actual RHE would increase due to the declined open-circuit potential with time, as shown in Fig. 1i. The truly calibrated potentials vs an actual RHE at 0 s, 40 s, 80 s, 120 s and 160 s where we collected TEM images were determined to be -1.1 V, -1.16 V, -1.17 V, -1.19 V and -1.20 V, respectively. Such truly calibrated potentials vs RHE during TEM measurement with time were marked in the images, as can be seen in Fig. 1e.

To validate the formation of Cu clusters originating from potential-driven restructuring rather than electron beam damage, we complemented in situ TEM measurement for N-Cu SAC during 1 h electrocatalysis at potentials more positive than -0.8 V vs RHE. Based on the discussions stated above, to realize the truly calibrated potential vs RHE no more negative than -0.8 V vs RHE, we applied -1.13 V vs Pt reference to achieve the calibrated potential of -0.4 V vs RHE at beginning. After 1 h, the truly calibrated potential vs RHE was -0.6 V as the CO poisoning reached a steady state with the open-circuit potential of 0.53 V vs Pt reference. Note that, such calibration vs RHE was only employed in liquid electrochemical TEM measurement with Pt as the quasi-reference electrode due to the significant CO poisoning effect on the potential drift.

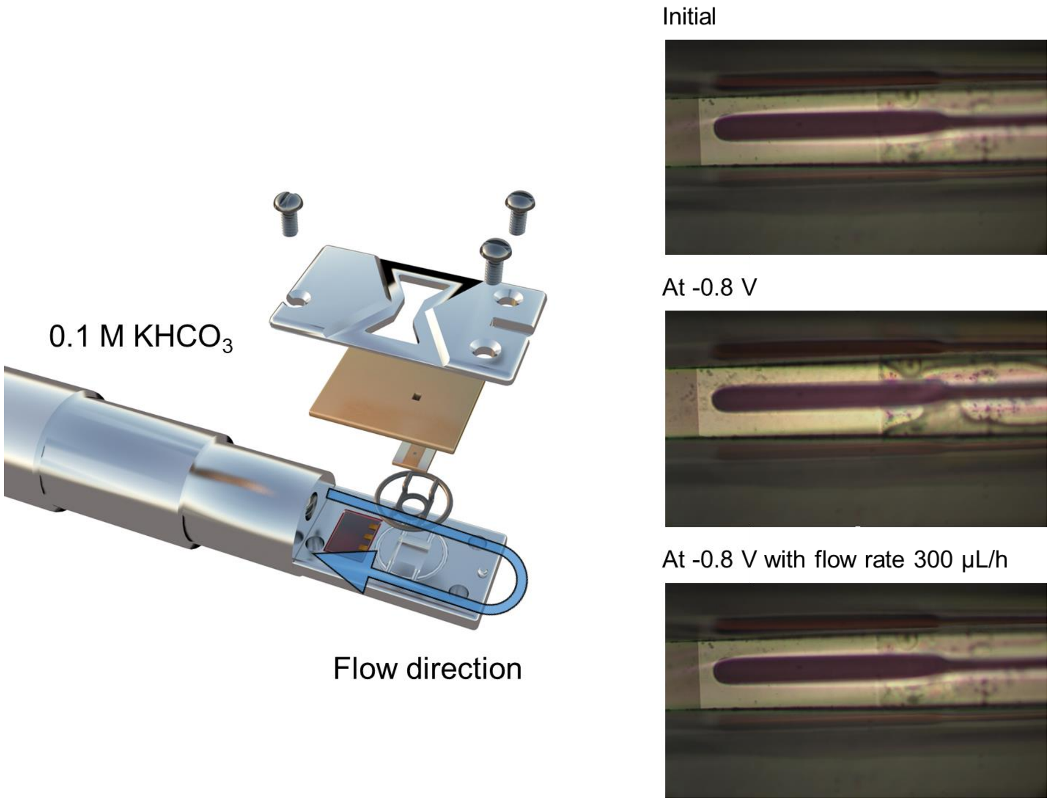

Figure S77. The effect of electrolyte flow on the bubbles. The schematic of the flow direction within the Poseidon select holder and the images from the SiN window under various conditions

Note: A clear demonstration of the influence of electrolyte flow on bubble movement was also provided as showcased in Movie S6. In the initial state, in the absence of external potentials and electrolyte flow, stationary electrolyte can be observed within the observation window. However, upon the application of potentials (-0.8 V vs RHE), bubbles become visible within the window. Remarkably, after one minute of introducing electrolyte injection at a flow rate of 300 μL/h, the size of the bubbles gradually decreases, ultimately vanishing within a few seconds. These findings convincingly illustrate that the presence of electrolyte flow effectively mitigates bubble formation during reactions.

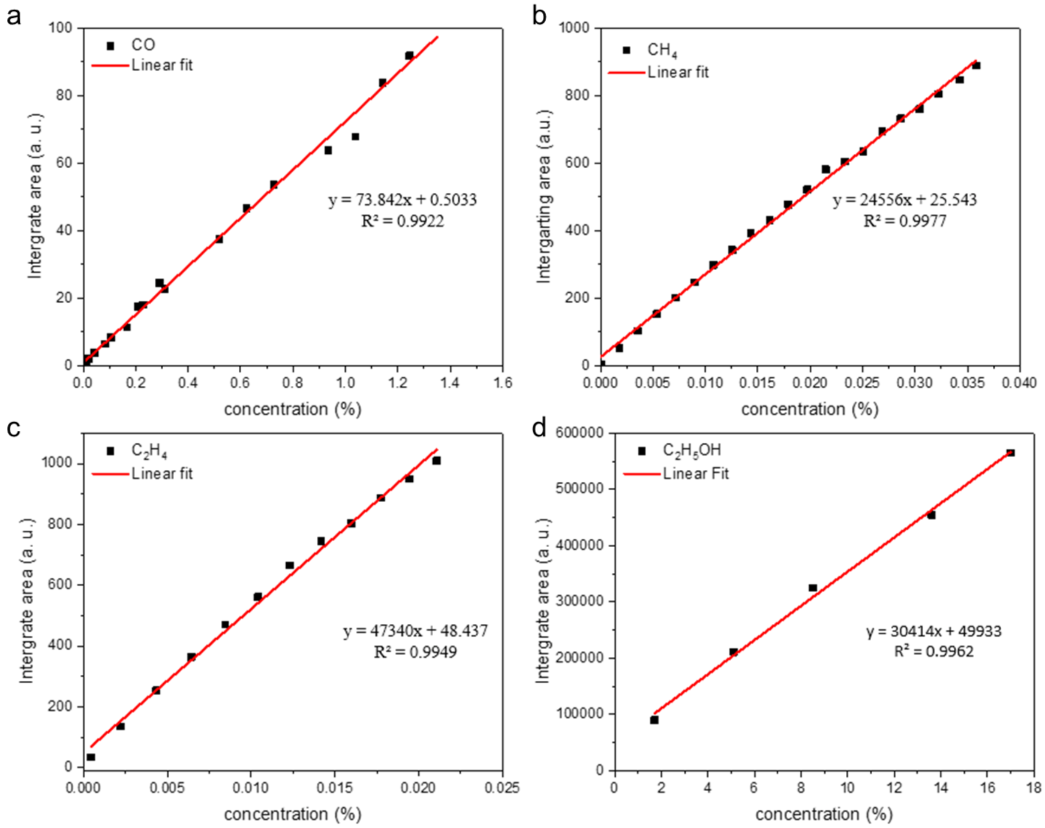

Figure S78. CO_2 reduction product analysis. Calibration curves of each CO_2 reduction product, (a) CO, (b) CH_4, (c) C_2H_4 and (d) C_2H_5OH.

Table S1. Structural parameters of N-Cu SAC extracted from Cu K-edge EXAFS refinement.

sample	path	R	N	dE	DW	R-factor
Cu SAC	Cu-N	1.93(3)	3.8(4)	-0.7(5)	0.0102(4)	3.862

Table S2. Structural parameters of N-Cu SAC extracted from in situ Cu K-edge EXAFS refinement at various applied potentials in 0.1 M CO_2-saturated $KHCO_3$.

sample	path	R	N	dE	DW	R-factor
Dry	Cu-N	1.93(3)	3.8(4)	-0.7(5)	0.0102(4)	3.862
	Cu-O	-	-	-	-	
	Cu-Cu	-	-	-	-	
OCV	Cu-N	1.93(6)	3.8(5)	-0.6(8)	0.0102(3)	4.148
	Cu-O	-	-	-	-	
	Cu-Cu	-	-	-	-	
-0.6V	Cu-N	1.93(3)	3.8(7)	-1.0(4)	0.0101(3)	2.087
	Cu-O	-	-	-	-	
	Cu-Cu	-	-	-	-	
-0.7V	Cu-N	1.93(3)	2.7(4)	-0.1(6)	0.0082(3)	4.443
	Cu-O	-	-	-	-	
	Cu-Cu	-	-	-	-	
-0.8V	Cu-N	1.94(4)	1.9(9)	4.4(4)	0.0041(4)	5.131
	Cu-O	2.10(4)	0.9(5)	9.8(8)	0.0087(6)	
	Cu-Cu	2.83(4)	0.6(6)	-9.4(9)	0.0088(4)	
-0.9V	Cu-N	2.14(3)	1.8(4)	5.1(4)	0.0050(4)	0.994
	Cu-O	1.93(6)	1.9(3)	12.5(3)	0.0050(9)	
	Cu-Cu	2.57(3)	1.2(4)	10.5(4)	0.0064(3)	
-1.0V	Cu-N	2.17(3)	0.9(2)	14.1(8)	0.0080(3)	1.747
	Cu-O	1.92(2)	1.3(8)	7.7(5)	0.0080(9)	
	Cu-Cu	2.55(4)	4.8(4)	3.7(3)	0.0082(2)	
-1.1V	Cu-N	2.17(5)	0.8(4)	9.1(3)	0.0077(6)	7.006
	Cu-O	1.92(4)	0.7(8)	7.7(6)	0.0077(4)	

Cu-Cu	2.56(2)	5.1(7)	4.3(7)	0.0078(2)

Note: For the correlation issue, one has to pay attention to the maximum freedom of the fitting parameter (N_{idp}). In present study, to fulfill the degree of freedom for achieving a reasonable fitting, we conducted the fitting for the first- and second-shell individually. For constrained fitting of the EXAFS data, according to a study of Charnock,[17] we assumed that the Debye-Waller factor would be identical in the same coordinated shell. That is, in the first shell fitting, the Debye-Waller factors of Cu-N and Cu-O paths were constrained to be an identical value, while the coordination number, atomic distance and energy shift were available for fitting. After obtaining the structural parameters of the first-shell, we set all parameters of first-shell according to the fitting results and then conducted the sequentially first-metallic-shell fitting for the Cu-Cu.

Table S3. Atomic coordinates of N-coordinated Cu SAC by Gaussian calculation.

C	4.781416	3.11E-06	0.021891
C	3.338168	-2.7E-06	-0.03181
C	2.685768	1.290449	-0.03977
C	2.685755	-1.29045	-0.0399
C	4.913281	2.440494	0.052755
C	3.481103	2.500683	0.018591
C	5.687849	3.626147	0.070746
C	2.864638	3.783822	0.002929
C	5.066841	4.858856	0.062555
H	6.770347	3.544531	0.080609
C	3.671934	4.933675	0.031106
H	5.654093	5.771209	0.076579
H	3.20781	5.912145	0.027222
C	4.913248	-2.44048	0.053144
C	3.481081	-2.50068	0.018655
C	5.687811	-3.62614	0.071428
C	2.864629	-3.78383	0.002823
C	5.066814	-4.85885	0.063146
H	6.770307	-3.54452	0.081575
C	3.67191	-4.93368	0.03128
H	5.654062	-5.7712	0.077406
H	3.207796	-5.91215	0.02731
C	0.71807	2.642002	-0.08008
C	-0.71807	2.642002	-0.08008
C	1.418883	3.858453	-0.03343
C	-1.41888	3.858453	-0.03343
C	0.687195	5.068772	-0.01324
C	-0.68719	5.068772	-0.01324
H	1.206898	6.018216	0.023732

H	-1.2069	6.018217	0.023733
C	0.718064	-2.64202	-0.08064
C	1.418878	-3.85843	-0.03391
C	-0.71806	-2.64202	-0.08064
C	0.687182	-5.06879	-0.01391
C	-1.41888	-3.85843	-0.03391
C	-0.68718	-5.06879	-0.01391
H	1.206912	-6.01822	0.022902
H	-1.20691	-6.01822	0.0229
C	-2.68576	-1.29045	-0.0399
C	-3.33817	-2.5E-06	-0.03181
C	-2.68577	1.290449	-0.03977
C	-3.4811	2.500683	0.018592
C	-2.86464	3.783822	0.002931
C	-4.91328	2.440494	0.052757
C	-3.67193	4.933675	0.031109
C	-5.68785	3.626148	0.07075
C	-5.06684	4.858857	0.06256
H	-3.20781	5.912146	0.027227
H	-6.77035	3.544531	0.080614
H	-5.65409	5.771209	0.076586
C	-3.48108	-2.50068	0.018654
C	-4.91325	-2.44048	0.053142
C	-2.86463	-3.78383	0.002821
C	-5.68781	-3.62614	0.071424
C	-3.67191	-4.93368	0.031276
C	-5.06681	-4.85885	0.063142
H	-6.77031	-3.54452	0.08157
H	-3.2078	-5.91215	0.027306
H	-5.65406	-5.7712	0.0774

C	-5.52626	-1.18746	0.058045
C	-4.78142	3.35E-06	0.021891
C	-5.52627	1.187464	0.057794
H	-6.60973	-1.12538	0.095309
H	-6.60975	1.125371	0.094992
C	5.526258	-1.18747	0.058047
C	5.526272	1.187463	0.057792
H	6.609732	-1.12538	0.09531
H	6.609748	1.125371	0.09499
Cu	-8.1E-07	-2.2E-05	-0.02482
N	-1.34223	-1.42013	-0.12519
N	-1.34221	1.420164	-0.12467
N	1.342211	1.420163	-0.12466
N	1.342228	-1.42013	-0.1252

Table S4. The energy and the configuration of MO by Gaussian calculation.

	MO	Energy (eV)	Cu(s)	Cu(p_x)	Cu(p_y)	Cu(p_z)	Cu(dx^2-y^2)	Cu(dz^2)	Cu(dxy)	Cu(dxz)	Cu(dyz)	N	C
HOMO	-19	-11.183	0	0.71	0	0	0	0	0	45.18	0	17.91	36.2
	-18	-11.029	0	0	0	0	0.08	0	0	0	0	3.59	96.33
	-17	-10.929	0	0	0.09	0	0	0	0	0	58.01	5.92	35.97
	-16	-10.842	0	2.06	0	0	0	0	0	26.43	0	33.12	38.39
	-15	-10.577	0.97	0	0	0.54	0	8.04	0.86	0	0	6.48	83.11
	-14	-10.405	10	0	0	0.07	0	65.87	0.12	0	0	7.97	15.97
	-13	-10.386	0	0	1.96	0	0	0	0	0	3.18	39.58	55.28
	-12	-10.232	0	0	0.25	0	0	0	0	0	6.38	6.88	86.49
	-11	-10.216	0	0.02	0	0	0	0	0	7.89	0	3.35	88.75
	-10	-10.075	0	0	0.02	0	0	0	0	0	8.1	2.52	89.36
	-9	-9.851	0	0	0	0	0.01	0	0	0	0	16.42	83.57
	-8	-9.413	0	0.01	0	0	0	0	0	5.8	0	16.49	77.69
	-7	-9.411	0	0	0	0.08	0	0	0.01	0	0	0.26	99.64
	-6	-9.345	0	0	0	0	0	0	0	2	0	4.77	93.23
	-5	-9.14	0	0	0	0.02	0	0	0.01	0	0	0.45	99.52
	-4	-8.879	0	0	0	0	0.03	0	0	0	0	1.3	98.66
	-3	-8.597	0	0	0.01	0	0	0	0	0	0.81	7.69	91.49
	-2	-7.358	0	0	0	0	0	0	0	1.08	0	13.08	85.84
	-1	-7.08	0.1	0	0	3.08	0	0.06	0	0	0	25.81	70.95

	0	-6.695	0	0	0	0	25.37	0	0	0	0	25.59	49.04
LUMO	0	-6.047	0	0	0.01	0	0	0	0	0	0.5	6.52	92.98
	1	-5.971	0	0	0	0	19.86	0	0	0	0	20.34	59.8
	2	-4.224	0	0	0	0	0.41	0	0	0	0	8.86	90.74
	3	-3.88	0.03	0	0	1.38	0	0	0	0	0	4.95	93.64
	4	-3.803	0	0	0.04	0	0	0	0	0	0.65	19.5	79.81
	5	-3.463	0	0	0.03	0	0	0	0	0	0.46	4.53	94.98
	6	-3.223	0	0.02	0	0	0	0	0	0.02	0	0.12	99.85
	7	-3.041	0.03	0	0	0.43	0	0	0	0	0	0.08	99.46
	8	-2.909	0	0.07	0	0	0	0	0	0.08	0	1.56	98.29
	9	-2.275	0	0	0	0	0.02	0	0	0	0	2.78	97.2
	10	-2.239	0.42	0	0	7.05	0	0	0.01	0	0	1.42	91.1
	11	-2.025	0	0.04	0	0	0	0	0	0.15	0	2.96	96.85
	12	-1.994	0	0	0	0	0.01	0	0	0	0	1.44	98.55
	13	-1.333	17.37	0	0	72.4	0	0.25	0	0	0	1.4	8.58
	14	-1.268	0	0	0.79	0	0	0	0	0	0.2	4.28	94.72
	15	-1.105	0	65.77	0	0	0	0	0	0.04	0	2.35	31.84
	16	-1.065	0	0	108.03	0	0	0	0	0	0.02	-0.52	-7.53
	17	-1.028	0	42.94	0	0	0	0	0	0.17	0	4.09	52.8
	18	-0.717	84.71	0	0	18.82	0	0.73	0	0	0	-2.49	-1.77
	19	-0.665	0	0	0	0	0.06	0	0	0	0	11.6	88.34

Table S5. Atomic coordinates of low N-coordinated Cu SAC by Gaussian calculation.

C	-2.76495	-3.42775	-0.00045
C	-4.95645	-2.25565	-0.00025
C	-3.5191	-2.21648	-0.00018
C	-5.65711	-1.03539	-8.8E-05
C	-2.83546	-0.97916	4.61E-05
C	-4.99307	0.205658	0.000112
H	-6.74345	-1.05077	-0.00012
C	-3.55258	0.240971	0.000176
C	-0.73373	-2.21833	3.62E-05
C	-1.41208	-0.9742	0.000207
C	0.733783	-2.21833	0.000396
C	-0.71387	0.266972	0.00013
C	1.412101	-0.97418	0.000184
C	0.713882	0.266973	0.000208
C	2.764982	-3.42769	0.000557
C	3.519151	-2.21639	0.000396
C	4.956485	-2.25555	0.000426
C	2.83547	-0.97912	0.000227
C	5.657126	-1.03531	0.000158
C	3.552575	0.241047	2.31E-06
C	4.993047	0.205758	-3.3E-05
H	6.743464	-1.05067	8.49E-05
C	5.600151	-3.51868	0.000729
H	6.684662	-3.56018	0.000847
C	-5.60008	-3.51875	-0.00055
H	-6.68459	-3.5603	-0.00055
Cu	7.75E-05	-4.83302	-0.0004
N	1.390172	-3.39398	0.000588
N	-1.39015	-3.39399	-0.00035

C	4.849649	-4.6848	0.00096
C	3.450723	-4.65207	0.000849
C	-3.45066	-4.65216	-0.00091
C	-4.84954	-4.68489	-0.00091
H	5.354578	-5.64581	0.001221
H	2.875391	-5.5742	0.000834
H	-2.87524	-5.57424	-0.00126
H	-5.35452	-5.64587	-0.00116
C	-5.68767	1.428232	0.000221
C	-2.85782	1.472239	0.000221
C	-1.42354	1.482472	0.000289
C	1.423516	1.482497	-4.2E-05
C	2.857799	1.472286	-4.6E-05
C	5.687625	1.428331	-0.00029
C	-5.01834	2.661238	0.000349
C	-3.57966	2.695438	0.000313
C	3.579609	2.695508	-0.0003
C	5.018277	2.661342	-0.00044
H	-6.77438	1.419488	0.000197
H	6.77434	1.419578	-0.00041
C	2.895178	3.944912	-0.0004
C	-2.89526	3.944866	0.000369
C	3.658378	5.116434	-0.00071
C	0.715273	2.731238	3.58E-05
C	1.432533	3.956491	-0.00021
C	-0.71533	2.73123	0.000132
C	-1.43261	3.956471	0.000169
C	-5.73825	3.885339	0.000499
C	-5.06356	5.086955	0.000661
H	-5.61362	6.022307	0.000813

C	-3.65849	5.116369	0.000606
H	-3.17227	6.084307	0.000788
C	5.738163	3.885455	-0.00072
C	5.06345	5.087059	-0.00087
H	5.613476	6.022428	-0.00112
H	3.172123	6.084356	-0.00087
H	6.823676	3.85785	-0.00085
H	-6.82376	3.857725	0.000489
C	0.69109	5.150298	-0.00026
C	-0.69118	5.150291	-8.5E-05
H	-1.19832	6.107147	-0.00023
H	1.198234	6.107158	-0.00044

Table S6. Structural parameters of N-Cu SAC in electrolyte condition and after removal of applied -0.7V in CO_2-saturated 0.1M $KHCO_3$ solution.

sample	path	R	N	dE	DW	R-factor
Dry	Cu-N	1.91(3)	3.8(4)	-1.8(5)	0.0099(9)	3.938
	Cu-O	-	-	-	-	
OCV	Cu-N	2.0(4)	3.3(3)	-1.1(8)	0.0097(5)	4.833
	Cu-O	1.9(6)	0.9(7)	7.6(3)	0.0060(4)	

Table S7. Structural parameters of Fe SAC extracted from in situ Fe K-edge EXAFS refinement at various applied potentials in 0.1 M CO_2-saturated $KHCO_3$.

sample	path	R	N	dE	DW	R-factor
Dry	Fe-N	1.98(4)	4.0(3)	-4.1(3)	0.0095(7)	2.475
		2.51(5)	3.9(2)	8.9(4)	0.0097(3)	
-0.2V	Fe-N	2.00(3)	3.9(3)	-5.3(3)	0.0113(6)	2.310
		2.50(5)	3.9(3)	8.3(5)	0.0091(3)	
-0.3V	Fe-N	2.05(6)	1.9(4)	-11.1(8)	0.0102(4)	6.341
		2.56(9)	3.9(6)	8.2(4)	0.0121(3)	
-0.4V	Fe-N	1.99(5)	1.9(5)	-19.3(4)	0.0090(5)	4.799
		2.52(6)	3.8(8)	-3.4(4)	0.0108(6)	
-0.5V	Fe-N	2.02(6)	1.9(4)	-15.7(7)	0.0101(7)	7.926
		2.53(5)	3.8(6)	5.7(3)	0.0114(4)	

Table S8. Structural parameters of Co SAC extracted from in situ Co K-edge EXAFS refinement at various applied potentials in 0.1 M CO_2-saturated $KHCO_3$.

sample	path	R	N	dE	DW	R-factor
Dry	Co-N	1.87(7)	3.8(4)	-2.1(4)	0.0120(6)	4.769
	Co-C	2.76(9)	4.1(4)	-5.9(7)	0.0139(6)	
-0.3V	Co-N	1.84(5)	3.2(3)	-15.9(5)	0.0100(9)	1.219
	Co-C	2.77(5)	3.9(9)	-5.2(7)	0.0134(7)	
-0.4V	Co-N	1.82(6)	2.0(4)	-2.2(4)	0.0108(4)	4.130
	Co-C	2.75(5)	3.9(7)	-7.7(6)	0.0131(4)	
-0.5V	Co-N	1.88(9)	1.8(3)	-8.4(6)	0.0122(4)	2.034
	Co-C	2.86(3)	3.9(9)	2.5(9)	0.0122(5)	
-0.6V	Co-N	1.85(8)	1.8(5)	-18.1(5)	0.0108(7)	4.914
	Co-C	2.83(5)	3.9(7)	-2.4(6)	0.0122(3)	

Table S9. Structural parameters of Zn SAC extracted from in situ Zn K-edge EXAFS refinement at various applied potentials in 0.1 M CO_2-saturated $KHCO_3$.

sample	path	R	N	dE	DW	R-factor
Dry	Zn-N	1.98(5)	3.9(7)	-7.9(8)	0.0091(7)	4.489
	Zn-C	2.83(6)	3.9(5)	-6.7(5)	0.0036(5)	
-0.4V	Zn-N	1.98(7)	4.0(9)	-7.3(6)	0.0091(7)	4.014
	Zn-C	2.88(6)	4.0(7)	-7.6(9)	0.0029(8)	
-0.5V	Zn-N	1.99(8)	3.9(6)	-7.1(8)	0.0087(9)	3.077
	Zn-C	2.84(8)	3.9(9)	-1.4(9)	0.0089(7)	
-0.6V	Zn-N	1.98(7)	4.0(9)	-7.3(6)	0.0091(5)	3.709
	Zn-C	2.88(6)	4.1(8)	-6.2(7)	0.0041(6)	
-0.7V	Zn-N	1.98(5)	4.0(9)	-6.9(8)	0.0090(8)	2.977
	Zn-C	2.80(5)	4.1(8)	-6.9(5)	0.0061(6)	

Supplementary References

1. Baran, E. J. Structural data and vibrational spectra of the Copper(II) complex of L-selenomethionine. *Z. Naturforschung B* **60**, 663–666 (2005).
2. Bodappa, N. *et al.* Early stages of electrochemical oxidation of Cu(111) and polycrystalline Cu surfaces revealed by in situ Raman spectroscopy. *J. Am. Chem. Soc.* **141**, 12192–12196 (2019).
3. Yang, P.-P. *et al.* Protecting copper oxidation state via intermediate confinement for selective CO_2 electroreduction to C2+ Fuels. *J. Am. Chem. Soc.* **142**, 6400–6408 (2020).
4. He, J. *et al.* Fast synthesis of hierarchical cuprous oxide for nonenzymatic glucose biosensors with enhanced sensitivity. *J. Mater. Sci.* **51**, 9696–9704 (2016).
5. Niaura, G. Surface-enhanced Raman spectroscopic observation of two kinds of adsorbed OH^- ions at copper electrode. *Electrochim. Acta.* **45**, 3507–3519 (2000).
6. Chen, X. *et al.* Controlling speciation during CO_2 reduction on Cu-alloy electrodes. *ACS. Catal.* **10**, 672–682 (2020).
7. Sander, T. *et al.* Correlation of intrinsic point defects and the Raman modes of cuprous oxide. *Phys. Rev. B* **90**, 045203 (2014).
8. Powell, D.*et al.* Raman-scattering study of ion-implantation-produced damage in Cu_2O. *Phys. Rev. B* **12**, 20–25 (1975).
9. Singhal, A. *et al.* Copper(I) oxide nanocrystals – one step synthesis, characterization, formation mechanism, and photocatalytic properties. *Eur. J. Inorg. Chem.* **2013**, 2640–2651 (2013).
10. Deng, Y. *et al.* In situ Raman spectroscopy of copper and copper oxide surfaces during electrochemical oxygen evolution reaction: Identification of Cu^{III} oxides as catalytically active species. *ACS Catal.* **6**, 2473–2481 (2016).
11. Coenen, K.,*et al.* An in-situ IR study on the adsorption of CO_2 and H_2O on hydrotalcites. *J. CO2 Util.* **24**, 228–239 (2018).
12. Ho, W. C. J. *et al.* Photocatalytic and adsorption performances of faceted cuprous oxide (Cu_2O) particles for the removal of methyl orange (MO) from aqueous media. *Molecules* **22**, 677 (2017).
13. Kau, L. S. *et al.* X-ray absorption edge determination of the oxidation state and coordination number of copper. Application to the type 3 site in Rhus vernicifera laccase and its reaction with oxygen. *J. Am. Chem. Soc.* **109**, 6433–6442 (1987).
14. Fei, H. *et al.* Atomic cobalt on nitrogen-doped graphene for hydrogen generation. *Nat. Commun* **6**, 1–8 (2015).

15. Zhang, L.-S. *et al.* Identification of the nitrogen species on N-doped graphene layers and Pt/NG composite catalyst for direct methanol fuel cell. *Phys. Chem. Chem. Phys.* **12**, 12055–5 (2010).
16. Bulushev, D. A. *et al.* Copper on carbon materials: stabilization by nitrogen doping. *J. Mater. Chem A* **5**, 10574–10583 (2017).
17. Charnock, J. Biological applications of EXAFS spectroscopy. *Radiation Physics and Chemistry* **45**, 385-391 (1995).
