# Supplementary material for: Activating dynamic atomic-configuration for single-site electrocatalyst in electrochemical CO2 reduction
Source: Nat Commun. 2023 Aug 28;14:5245. doi: 10.1038/s41467-023-40970-y (PMC10462635; doi:10.1038/s41467-023-40970-y)
Supplement: Supplementary file 2 — Description of Additional Supplementary Files [file 41467_2023_40970_MOESM2_ESM.pdf]

Description of Additional Supplementary Files

Supplementary Movie 1: Dynamic restructuring of N-Cu SAC at -1.1 V vs RHE in electrolyte.

Supplementary Movie 2: N-Cu SAC at -1.1 V vs RHE in electrolyte for longer duration.

Supplementary Movie 3: N-Cu SAC at -0.6 V vs RHE in electrolyte.

Supplementary Movie 4: Control experiment without applying cathodic potentials in electrolyte.

Supplementary Movie 5: Control experiment without introducing electrolyte.

Supplementary Movie 6: Effect of electrolyte flow on the bubble movement during reaction.
